# Supplementary material for: YTHDC1 promotes postnatal brown adipose tissue development and thermogenesis by stabilizing PPARγ
Source: EMBO J. 2025 May 12;44(12):3360–80. doi: 10.1038/s44318-025-00460-x (PMC12170836; doi:10.1038/s44318-025-00460-x)
Supplement: Supplementary file 1 — Appendix [file 44318_2025_460_MOESM1_ESM.pdf]

Appendix for

YTHDC1 promotes postnatal brown adipose tissue development and thermogenesis by stabilizing PPARγ

Table of Contents

Appendix Figure S1……………………………..…………………………………………………2

Appendix Figure S2……………………………..…………………………………………………3

Appendix Figure S3……………………………..…………………………………………………4

Appendix Figure S4……………………………..…………………………………………………6

Appendix Figure S5……………………………..…………………………………………………7

Appendix Figure S6……………………………..…………………………………………………8

Appendix Figure S7……………………………..…………………………………………………9

Appendix Figure S8……………………………..………………………………………………..11

Appendix Table S1………………..………………..………………………………………………..13

A

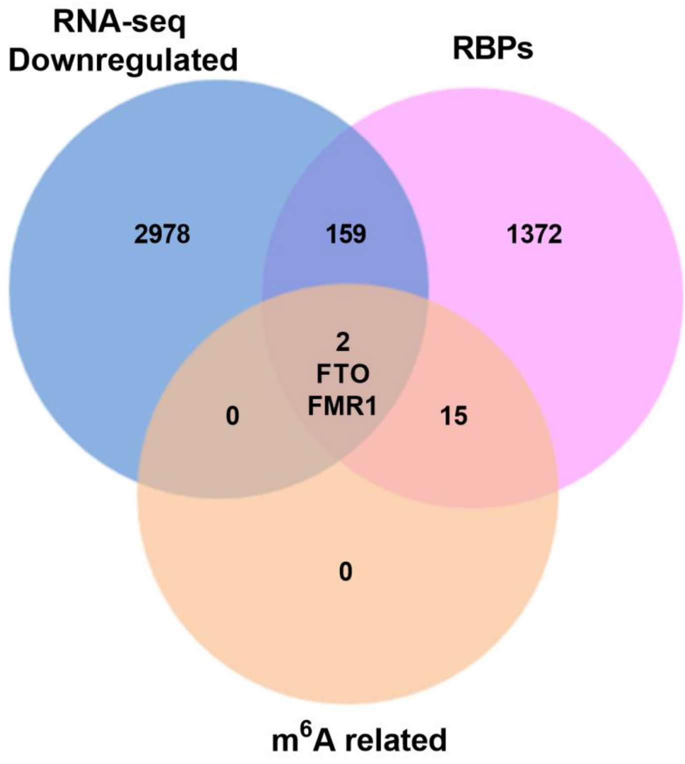

B

GO analysis of upregulated RBPs

GO Biological Process Term	Count	p Value
RNA processing	182	5.73E-149
RNA metabolic process	218	2.56E-143
translation	137	1.02E-140
ribonucleoprotein complex biogenesis	123	2.79E-113
ribosome biogenesis	99	1.14E-94
RNA splicing	65	4.56E-56
regulation of RNA stability	40	1.58E-31

C

YTHDF2	HNRNPA2B1	YTHDC1	METTL14
YTHDF3	YTHDF1	METTL3	RBM15

Appendix Figure S1. Cold exposure changes the expression of RNA-binding proteins in iBAT.

(A) A Venn diagram revealed that showed that 161 RBPs and 2 m^6A related RBPs (FTO and FMR1) in iBAT were downregulated during cold exposure. (B) GO analysis of upregulated RBPs. (C) Upregulated m^6A related RBPs were shown.

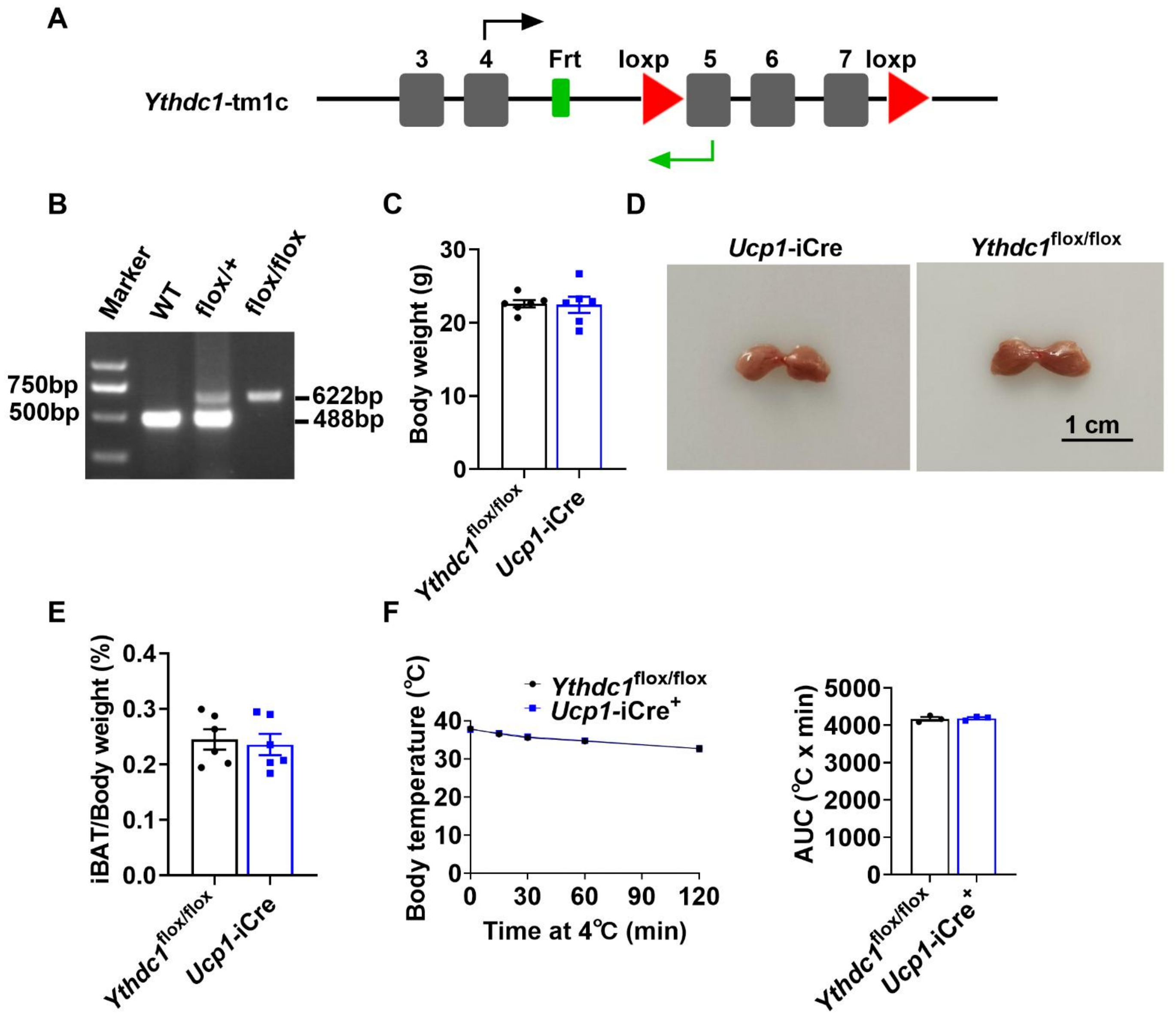

Appendix Figure S2. Generation and phenotyping of *Ythdc1*$^{flox/flox}$, *Ucp1*-iCre, and *Ythdc1*-BKO mice.

(A) The exons 5-7 of the *Ythdc1* gene were flanked by two loxp sites (red triangles). (B) Genotyping of WT, *Ythdc1*$^{flox/+}$ and *Ythdc1*$^{flox/flox}$ mice. (C) Body weight of *Ythdc1*$^{flox/flox}$ and *Ucp1*-iCre mice was measured (n=6 per group). (D) Morphology of iBAT from *Ucp1*-iCre and *Ythdc1*$^{flox/flox}$ mice at 8 weeks old. (E) Relative iBAT weight was measured (n=6 per group). (F) The rectal temperature was determined in 8-week-old *Ucp1*-iCre and *Ythdc1*$^{flox/flox}$ mice during acute cold exposure (4℃). AUC was measured (n=3 per group). Data represent the mean ± SEM. n was the number of biologically independent mice.

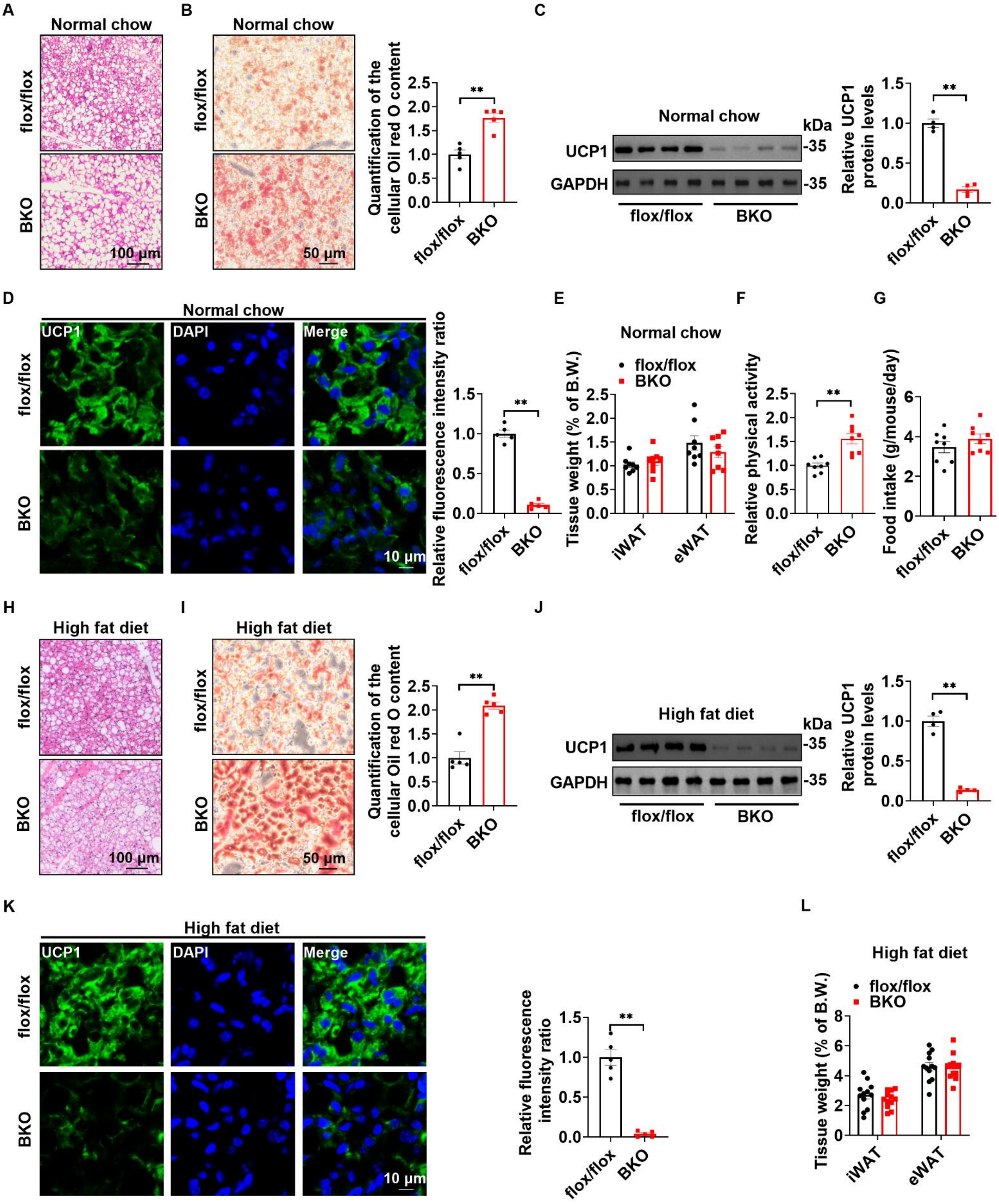
A
Normal chow
flox/flox
BKO
100 μm
B
Normal chow
flox/flox
BKO
50 μm
Quantification of the cellular Oil red O content
flox/flox
BKO
**
C
Normal chow
kDa
UCP1
GAPDH
-35
flox/flox
BKO
Relative UCP1 protein levels
**
D
Normal chow
UCP1
DAPI
Merge
flox/flox
BKO
10 μm
Relative fluorescence intensity ratio
**
E
Normal chow
flox/flox
BKO
Tissue weight (% of B.W.)
iWAT
eWAT
F
Relative physical activity
**
G
Food intake (g/mouse/day)
H
High fat diet
flox/flox
BKO
100 μm
I
High fat diet
50 μm
Quantification of the cellular Oil red O content
**
J
High fat diet
kDa
UCP1
GAPDH
-35
flox/flox
BKO
Relative UCP1 protein levels
**
K
High fat diet
UCP1
DAPI
Merge
10 μm
Relative fluorescence intensity ratio
**
L
High fat diet
flox/flox
BKO
Tissue weight (% of B.W.)
iWAT
eWAT

Appendix Figure S3. BAT-specific deletion of *Ythdc1* leads to decreased UCP1 expression in iBAT without affecting WAT.

(A-B) H&E and Oil Red O staining in iBATs of *Ythdc1*$^{flox/flox}$ and *Ythdc1*-BKO mice fed with an NC diet (n=5 per group; For Oil Red O staining, $P = 0.0079$). (C) UCP1 and GAPDH protein levels in iBATs of *Ythdc1*$^{flox/flox}$ and *Ythdc1*-BKO mice fed with an NC diet (n=4 per group; $P < 0.0001$). (D) UCP1 immunostaining in iBATs of *Ythdc1*$^{flox/flox}$ and *Ythdc1*-BKO mice fed with an NC diet (n=5 per group; $P < 0.0001$). (E) The weights of iWAT and eWAT in *Ythdc1*$^{flox/flox}$ and *Ythdc1*-BKO mice fed with an NC diet were measured (n=8 per group). (F) Relative physical activity was measured in *Ythdc1*$^{flox/flox}$ and *Ythdc1*-BKO mice at 8 weeks old using metabolic cages (n=8 per group; $P = 0.0004$). (G) Food intake was assayed in *Ythdc1*$^{flox/flox}$ and *Ythdc1*-BKO mice at 8 weeks old using metabolic cages (n=8 per group). (H-I) H&E and Oil Red O staining in iBATs of *Ythdc1*$^{flox/flox}$ and *Ythdc1*-BKO mice fed with an HFD (n=5 per group; For Oil Red O staining, $P = 0.0079$). (J) UCP1 and GAPDH protein levels in iBATs of *Ythdc1*$^{flox/flox}$ and *Ythdc1*-BKO mice fed with an HFD (n=4 per group; $P < 0.0001$). (K) UCP1 immunostaining in iBATs of *Ythdc1*$^{flox/flox}$ and *Ythdc1*-BKO mice fed with an HFD (n=5 per group; $P < 0.0001$). (L) The weights of iWAT and eWAT in *Ythdc1*$^{flox/flox}$ and *Ythdc1*-BKO mice fed with an HFD were measured (n=12 per group). Data represent the mean ± SEM. n was the number of biologically independent mice. Differences between two groups were analyzed by unpaired two-tailed Student's *t* tests. *, $P< 0.05$. **, $P< 0.01$.

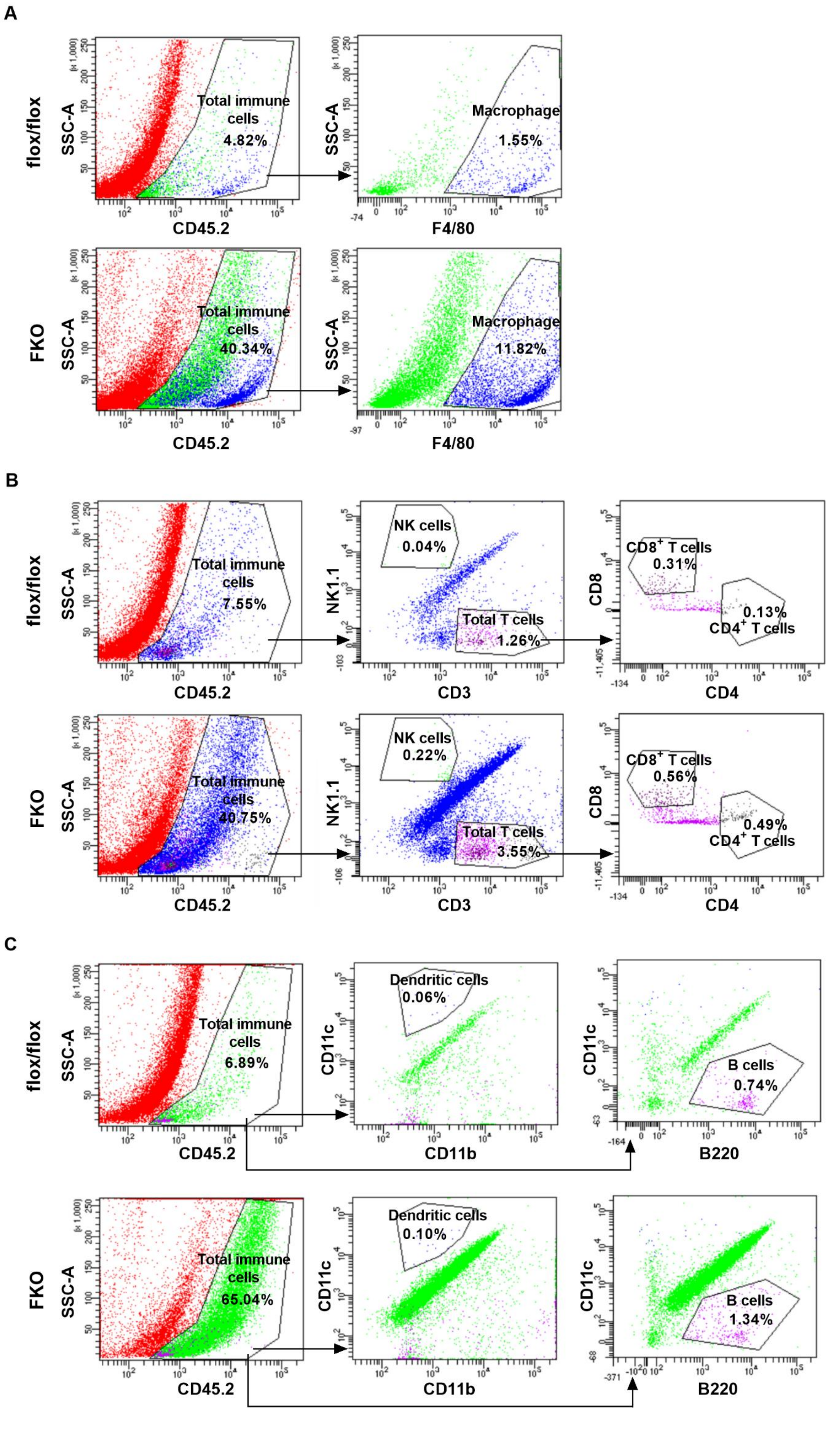
A
flox/flox
SSC-A
Total immune cells
4.82%
CD45.2
Macrophage
1.55%
F4/80
FKO
Total immune cells
40.34%
Macrophage
11.82%
B
NK cells
0.04%
Total T cells
1.26%
NK1.1
CD3
CD8⁺ T cells
0.31%
0.13%
CD4⁺ T cells
CD8
CD4
7.55%
40.75%
0.22%
3.55%
0.56%
0.49%
C
Dendritic cells
0.06%
CD11c
CD11b
B cells
0.74%
B220
6.89%
65.04%
0.10%
1.34%

Appendix Figure S4. Fat-specific deletion of *Ythdc1* increases the immune cells in iBAT.

(A-C) SVFs were isolated from iBATs of *Ythdc1*$^{flox/flox}$ and *Ythdc1*-FKO mice, and FACS analysis was performed. (A) Representative FACS plots of staining for total immune ($CD45.2^{+}$) cells and macrophage ($CD45.2^{+}F4/80^{+}$) cells were presented. (B) Representative FACS plots of staining for NK ($CD45.2^{+}$ $NK1.1^{+}$ $CD3^{-}$) cells, total T ($CD45.2^{+}CD3^{+}$) cells, CD4 T ($CD45.2^{+}CD3^{+}CD4^{+}$) cells, and CD8 T ($CD45.2^{+}$ $CD3^{+}CD8^{+}$) cells were presented. (C) Representative FACS plots of staining for dendritic cells ($CD11c^{+}$ $CD11b^{-}$ cells) and B cells ($B220^{+}$ $CD11c^{-}$) cells were presented.

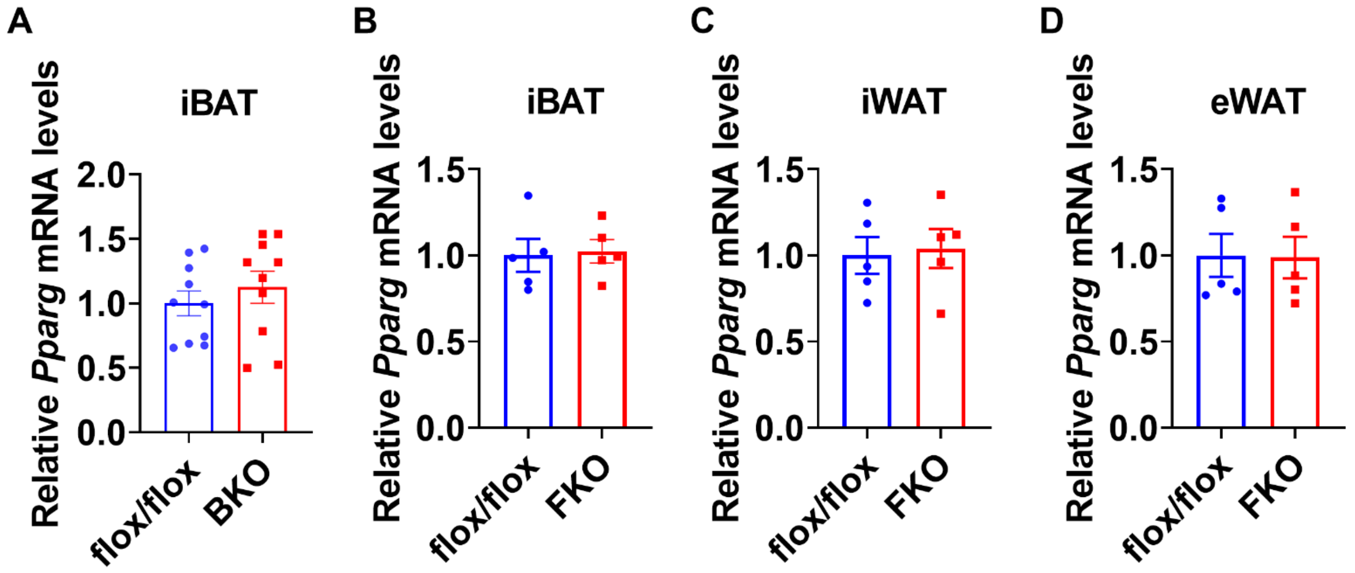

Appendix Figure S5. BAT- or fat-specific deletion of *Ythdc1* does not affect *Pparg* mRNA levels.

(A) *Pparg* mRNA levels were measured by RT-qPCR in iBATs of *Ythdc1*$^{flox/flox}$ and *Ythdc1*-BKO mice at 8 weeks of age (n=10 per group). (B) *Pparg* mRNA levels were measured by RT-qPCR in iBATs of *Ythdc1*$^{flox/flox}$ and *Ythdc1*-FKO mice at 8 weeks of age (n=5 per group). (C) *Pparg* mRNA levels were measured by RT-qPCR in iWATs of *Ythdc1*$^{flox/flox}$ and *Ythdc1*-FKO mice at 8 weeks of age (n=5 per group). (D) *Pparg* mRNA levels were measured by RT-qPCR in eWATs of *Ythdc1*$^{flox/flox}$ and *Ythdc1*-FKO mice at 8 weeks of age (n=5 per group). Data represent the mean ± SEM. n was the number of biologically independent mice.

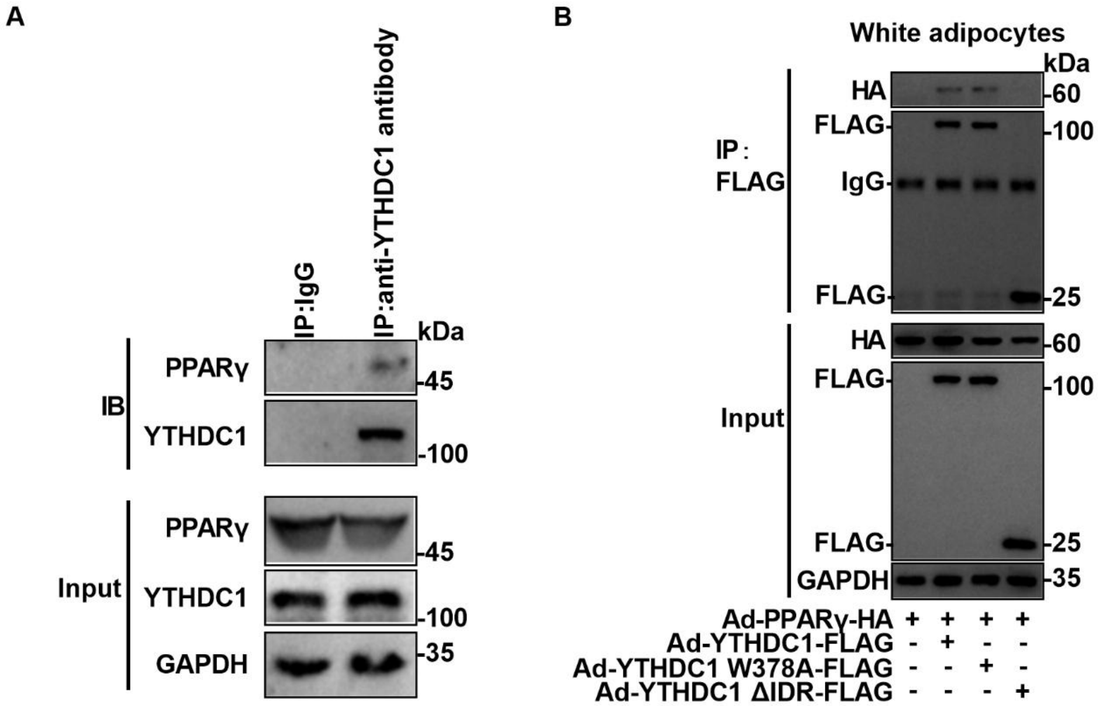

Appendix Figure S6. YTHDC1 interacts with PPARγ in both iBAT and primary white adipocytes.

(A) Tissue lysates were extracted from iBATs of C57BL6 WT mice in RIPA buffer. Tissue lysates were immunoprecipitated with anti-YTHDC1 or IgG antibodies, and then immunoblotted with anti-PPARγ or anti-YTHDC1 antibodies. Input samples were immunoblotted with anti-PPARγ, anti-YTHDC1, or anti-GAPDH antibodies. The samples were derived from the same experiment and the blots were processed in parallel.

(B) Primary white adipocytes were differentiated from white fat stromal-vascular fraction (SVF). Ad-PPARγ-HA adenovirus was co-infected with Ad-βGal, Ad-YTHDC1-FLAG, Ad-YTHDC1W378A-FLAG, or Ad-YTHDC1ΔIDR-FLAG adenovirus for 48 hours. Cell lysates were extracted from these cells in RIPA buffer. These lysates were immunoprecipitated with anti-FLAG beads and then immunoblotted with anti-HA or anti-FLAG antibodies. The samples were derived from the same experiment and the blots were processed in parallel. All the cell culture experiments were repeated three times with similar results.

A

Gene symbol	Gene description	Species	Confidence score	Confidence level
ARIH2	E3 ubiquitin-protein ligase ARIH2	H.sapiens	0.912	HIGH
MDM2	E3 ubiquitin-protein ligase Mdm2	H.sapiens	0.897	HIGH
BARD1	BRCA1-associated RING domain protein 1	H.sapiens	0.894	HIGH
RANBP2	E3 SUMO-protein ligase RanBP2	H.sapiens	0.891	HIGH
MDM4	Protein Mdm4	H.sapiens	0.885	HIGH
STUB1	E3 ubiquitin-protein ligase CHIP	H.sapiens	0.875	HIGH
BRCA1	Breast cancer type 1 susceptibility protein	H.sapiens	0.864	HIGH
SMURF2	E3 ubiquitin-protein ligase SMURF2	H.sapiens	0.863	HIGH
ITCH	E3 ubiquitin-protein ligase Itchy homolog	H.sapiens	0.863	HIGH
RBBP6	E3 ubiquitin-protein ligase RBBP6	H.sapiens	0.853	HIGH
SIAH1	E3 ubiquitin-protein ligase SIAH1	H.sapiens	0.847	HIGH
HECW1	E3 ubiquitin-protein ligase HECW1	H.sapiens	0.845	HIGH
RBX1	E3 ubiquitin-protein ligase RBX1	H.sapiens	0.841	HIGH
SYVN1	E3 ubiquitin-protein ligase synoviolin	H.sapiens	0.841	HIGH
TOPORS	E3 ubiquitin-protein ligase Topors	H.sapiens	0.841	HIGH
TRAF2	TNF receptor-associated factor 2	H.sapiens	0.833	HIGH
UBE3A	Ubiquitin-protein ligase E3A	H.sapiens	0.833	HIGH
MNAT1	CDK-activating kinase assembly factor MAT1	H.sapiens	0.825	HIGH
RLIM	E3 ubiquitin-protein ligase RLIM	H.sapiens	0.825	HIGH
UHRF2	E3 ubiquitin-protein ligase UHRF2	H.sapiens	0.825	HIGH
RCHY1	RING finger and CHY zinc finger domain-containing protein 1	H.sapiens	0.825	HIGH
PML	Protein PML	H.sapiens	0.825	HIGH
ZEB2	Zinc finger E-box-binding homeobox 2	H.sapiens	0.823	HIGH
RNF14	E3 ubiquitin-protein ligase RNF14	H.sapiens	0.818	HIGH
CBL	E3 ubiquitin-protein ligase CBL	H.sapiens	0.814	HIGH
HSPA8	Heat shock cognate 71 kDa protein	H.sapiens	0.814	HIGH
ABTB1	Ankyrin repeat and BTB/POZ domain-containing protein 1	H.sapiens	0.813	HIGH
PRKN	E3 ubiquitin-protein ligase parkin	H.sapiens	0.812	HIGH
RNF31	E3 ubiquitin-protein ligase RNF31	H.sapiens	0.812	HIGH
ZMYND11	Zinc finger MYND domain-containing protein 11	H.sapiens	0.81	HIGH
SPOPL	Speckle-type POZ protein-like	H.sapiens	0.807	HIGH
RAG1	V(D)J recombination-activating protein 1	H.sapiens	0.806	HIGH

B

ARIH2
BARD1
MDM4
STUB1/CHIP
SMURF2
ITCH
RBBP6
SIAH1
RBX1
SYVN1
TOPORS
UBE3A
MNAT1
RLIM
UHRF2

C

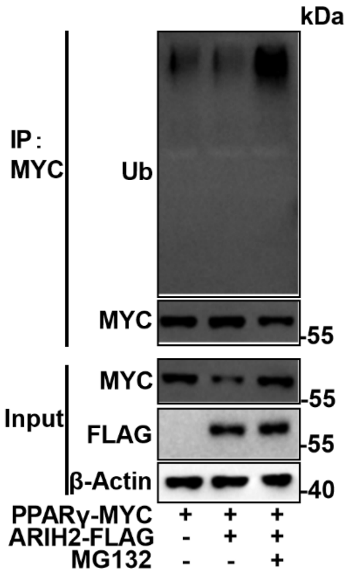

Appendix Figure S7. List of E3 ubiquitin ligases that may regulate PPARγ.

(A) We used UbiBrowser[2.0] to predicted ubiquitin ligases that may regulate PPARγ. The predicted thirty-two E3 ligases were shown. (B) Fifteen E3 ligases located in nuclei and expressed in BAT were listed. (C) PPARγ-MYC was co-transfected with or without ARIH2-FLAG in HEK293T cells for 30 hours. Cells were treated with or without MG132 (20 μM) for 6 hours. Cell lysates were extracted from these cells in RIPA buffer. These lysates were immunoprecipitated with anti-MYC beads and then immunoblotted with anti-Ubiquitin or anti-MYC antibodies. The samples were derived from the same experiment and the blots were processed in parallel. The cell culture experiments were repeated three times with similar results.

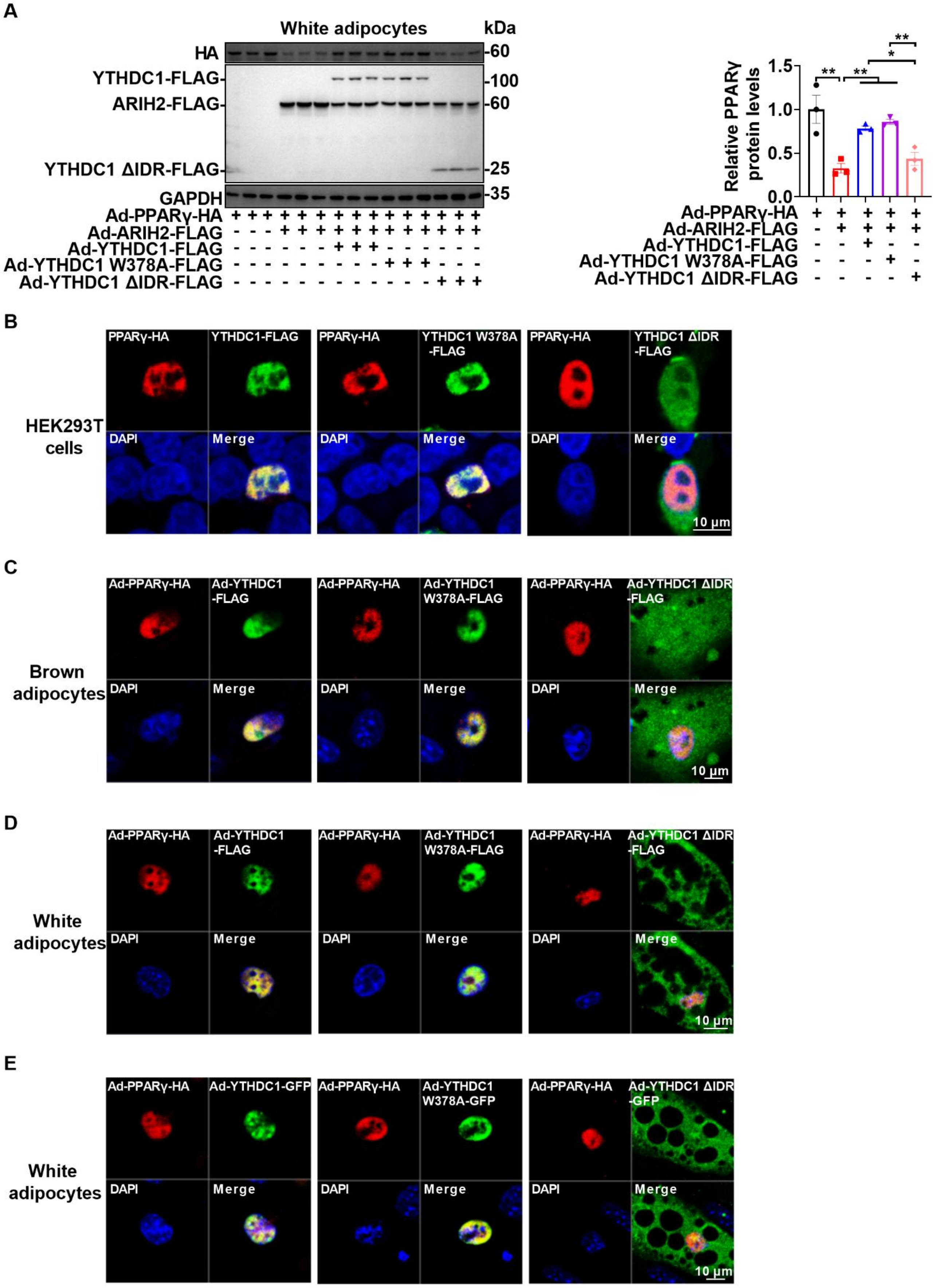
A
White adipocytes
kDa
HA
YTHDC1-FLAG
ARIH2-FLAG
YTHDC1 ΔIDR-FLAG
GAPDH
-60
-100
-60
-25
-35
Ad-PPARγ-HA
Ad-ARIH2-FLAG
Ad-YTHDC1-FLAG
Ad-YTHDC1 W378A-FLAG
Ad-YTHDC1 ΔIDR-FLAG
Relative PPARγ protein levels
1.5
1.0
0.5
0.0
**
**
*
B
HEK293T cells
PPARγ-HA
YTHDC1-FLAG
YTHDC1 W378A -FLAG
YTHDC1 ΔIDR -FLAG
DAPI
Merge
10 μm
C
Brown adipocytes
Ad-PPARγ-HA
Ad-YTHDC1 -FLAG
Ad-YTHDC1 W378A-FLAG
Ad-YTHDC1 ΔIDR -FLAG
D
White adipocytes
E
White adipocytes
Ad-YTHDC1-GFP
Ad-YTHDC1 W378A-GFP
Ad-YTHDC1 ΔIDR -GFP

Appendix Figure S8. IDR is required for YTHDC1 to protect PPARγ degradation by ARIH2.

(A) Primary white adipocytes were differentiated from white fat stromal-vascular fraction (SVF). Ad-PPARγ-HA adenovirus was co-infected with Ad-βGal, Ad-YTHDC1-FLAG, Ad-YTHDC1W378A-FLAG, or Ad-YTHDC1ΔIDR-FLAG adenovirus for 24 hours. Cells were then infected with or without Ad-ARIH2-FLAG adenovirus. Twenty-four hours later, cell lysates were extracted from these cells in RIPA buffer. These lysates were immunoblotted with anti-HA, anti-FLAG, or anti-GAPDH antibodies. The samples were derived from the same experiment and the blots were processed in parallel (n=3 per group). (B) PPARγ-HA expression vector was co-transfected with YTHDC1-FLAG, YTHDC1 W378A-FLAG, or YTHDC1ΔIDR-FLAG expression vectors in HEK293T cells for 30 hours. Cells were co-immunostained with anti-HA and anti-FLAG antibodies. The co-localization of PPARγ and different forms of YTHDC1 was analyzed using confocal microscopy. (C) Primary brown adipocytes were differentiated from brown fat stromal-vascular fraction (SVF). Ad-PPARγ-HA adenovirus was co-infected with Ad-βGal, Ad-YTHDC1-FLAG, Ad-YTHDC1W378A-FLAG, or Ad-YTHDC1ΔIDR-FLAG adenovirus for 48 hours. Cells were co-immunostained with anti-HA and anti-FLAG antibodies. The co-localization of PPARγ and different forms of YTHDC1 was analyzed using confocal microscopy. (D) Primary white adipocytes were differentiated from white fat stromal-vascular fraction (SVF). Ad-PPARγ-HA adenovirus was co-infected with Ad-βGal, Ad-YTHDC1-FLAG, Ad-YTHDC1W378A-FLAG, or Ad-YTHDC1ΔIDR-FLAG adenovirus for 48 hours. Cells were co-immunostained with anti-HA and anti-FLAG antibodies. The co-localization of PPARγ and different forms of YTHDC1 was analyzed using confocal microscopy. (E) Primary white adipocytes were differentiated from white fat SVF. Ad-PPARγ-HA adenovirus was co-infected with Ad-βGal, Ad-YTHDC1-GFP, Ad-YTHDC1W378A-GFP, or Ad-YTHDC1ΔIDR-GFP adenovirus for 48 hours. Cells were immunostained with anti-HA antibody. The co-localization of HA-PPARγ and different forms of YTHDC1-GFP was analyzed using confocal microscopy. All the cell culture experiments were repeated three times with similar results. n

was the number of biologically independent cell samples. Data represent the mean ± SEM. Differences among more than two groups were analyzed by one-factor analysis of variance (ANOVA). *, $P< 0.05$. **, $P< 0.01$.

Appendix Table S1. Primers for qPCR.

**Genes**	**Forward**	**Reverse**
36B4	5'-AAGCGCGTCCTGGCATTGTCT-3'	5'-CCGCAGGGGCAGCAGTGGT-3'
Ythdc1	5'- CGGGAGGAGAAAGATGGGGA-3'	5'- TGTCGCTTGGTGTCAGTAGAC-3'
Pparg	5'-TGGGTGAAACTCTGGGAGATTC-3'	5'- GAGAGGTCCACAGAGCTGATTCC-3'
Fabp4	5'- AAGGAAAGTGGCAGGCATGG -3'	5'-CACGCCCAGTTTGAAGGAAATC-3'
Plin2	5'- CAGCTCTCCTGTTAGGCGT -3'	5'- GCCATCTCACACACGGATCT -3'
CD36	5'- GGAGTGGTGATGTTTGTTGCT -3'	5'- GCACACACCACCATTTCTTCT -3'
Glut4	5'-TCTCCAACTGGACCTGTAAC-3'	5'-TCTGTACTGGGTTTCACCTC-3'
Adipoq	5'- GCACTGGCAAGTTCTACTGCAA-3'	5'- GTAGGTGAAGAGAACGGCCTTGT-3'
Cd14	5'- CTTAAAGCGGCTTACGGTGC-3'	5'- ACGTTGCGGAGGTTCAAGAT-3'
Ccl6	5'- ATGCCACACAGATCCCATGT-3'	5'- CTGAACTCTCCGATCGCTGG -3'
Cx3cl1	5'- GCAAGTTTGAGAAGCGGGTG -3'	5'- CTTGGGAAGTCCCCATGGTC -3'
Csf1	5'- TGGCTTGGCTTGGGATGATT-3'	5'- GTCTGTCCCCATGGTTTGGT-3'
Ccl5	5'-CCACTTCTTCTCTGGGTTGG-3'	5'-GTGCCCACGTCAAGGAGTAT-3'
Ccr2	5'-GCCATCATAAAGGAGCCATACCT-3'	5'- ATGCCGTGGATGAACTGAGG -3'
