# Supplementary figures and images for: YTHDC1 promotes postnatal brown adipose tissue development and thermogenesis by stabilizing PPARγ (part 1 of 3)
Source: EMBO J. 2025 May 12;44(12):3360–80. doi: 10.1038/s44318-025-00460-x (PMC12170836; doi:10.1038/s44318-025-00460-x)

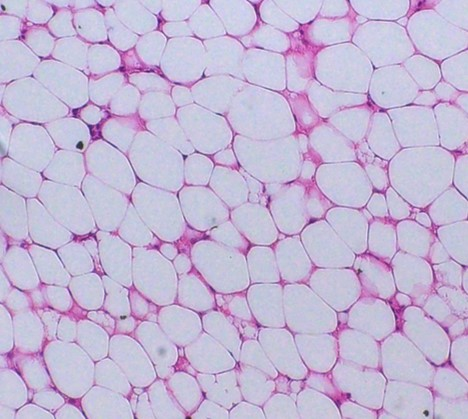

Supplement: Supplementary file 4 — Source data Fig. 1 [file 44318_2025_460_MOESM4_ESM.zip › Figure1/1O/BKO iWAT.tif]

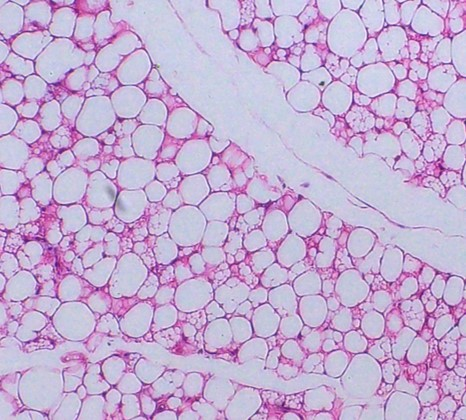

Supplement: Supplementary file 4 — Source data Fig. 1 [file 44318_2025_460_MOESM4_ESM.zip › Figure1/1O/FF iWAT.tif]

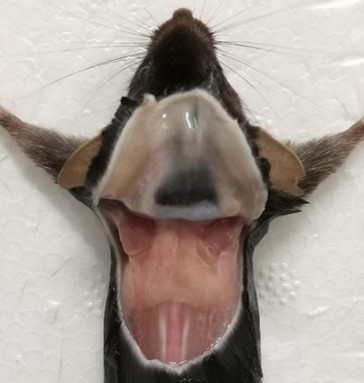

Supplement: Supplementary file 4 — Source data Fig. 1 [file 44318_2025_460_MOESM4_ESM.zip › Figure1/1E/FF Appearance.tif]

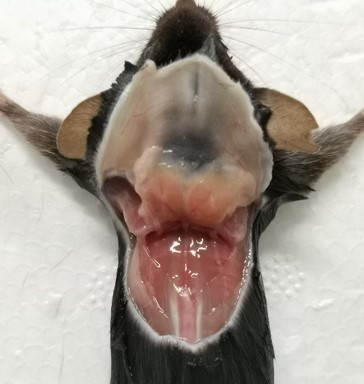

Supplement: Supplementary file 4 — Source data Fig. 1 [file 44318_2025_460_MOESM4_ESM.zip › Figure1/1E/FF Appearance flip.tif]

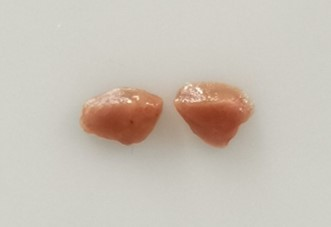

Supplement: Supplementary file 4 — Source data Fig. 1 [file 44318_2025_460_MOESM4_ESM.zip › Figure1/1E/FF iBAT.tif]

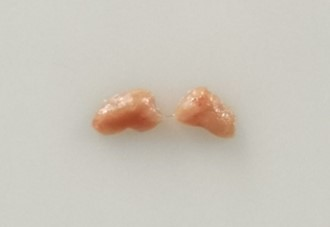

Supplement: Supplementary file 4 — Source data Fig. 1 [file 44318_2025_460_MOESM4_ESM.zip › Figure1/1E/BKO iBAT.tif]

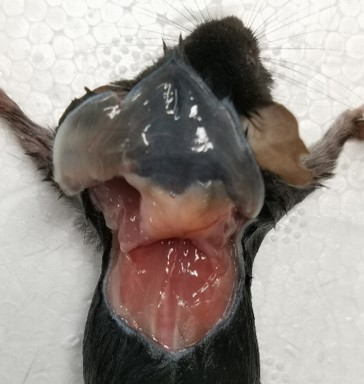

Supplement: Supplementary file 4 — Source data Fig. 1 [file 44318_2025_460_MOESM4_ESM.zip › Figure1/1E/BKO Appearance flip.tif]

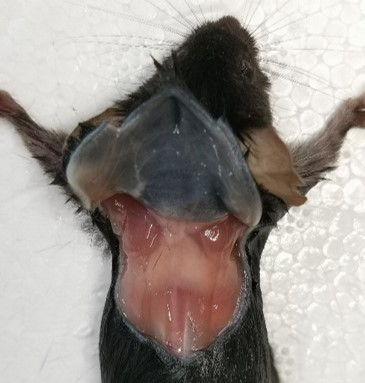

Supplement: Supplementary file 4 — Source data Fig. 1 [file 44318_2025_460_MOESM4_ESM.zip › Figure1/1E/BKO Appearance.tif]

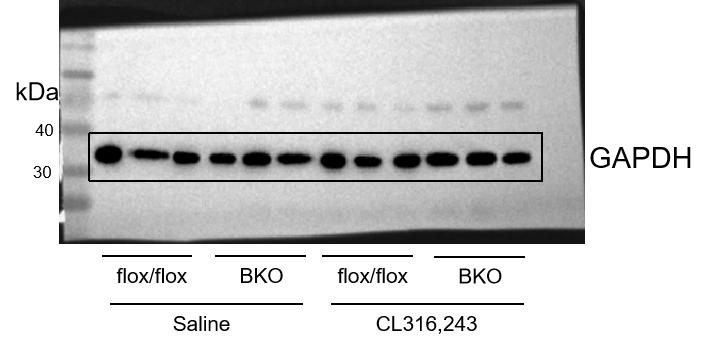

Supplement: Supplementary file 4 — Source data Fig. 1 [file 44318_2025_460_MOESM4_ESM.zip › Figure1/1P/western GAPDH .tif]

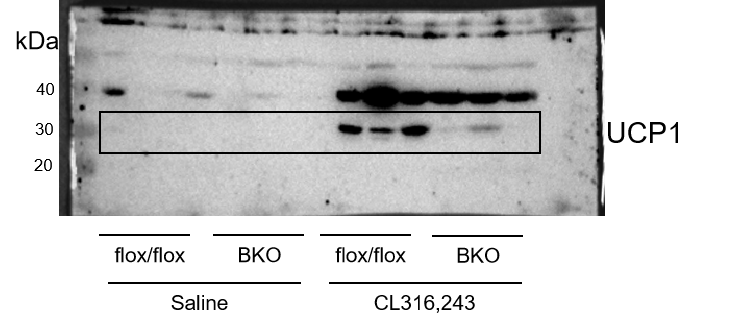

Supplement: Supplementary file 4 — Source data Fig. 1 [file 44318_2025_460_MOESM4_ESM.zip › Figure1/1P/western UCP1 .tif]

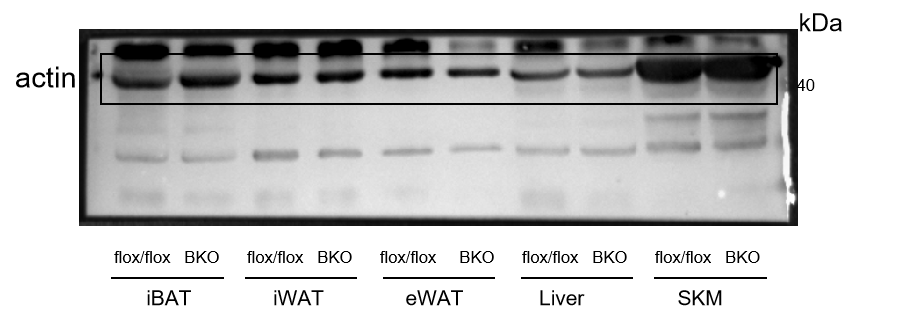

Supplement: Supplementary file 4 — Source data Fig. 1 [file 44318_2025_460_MOESM4_ESM.zip › Figure1/1D/western actin.tif]

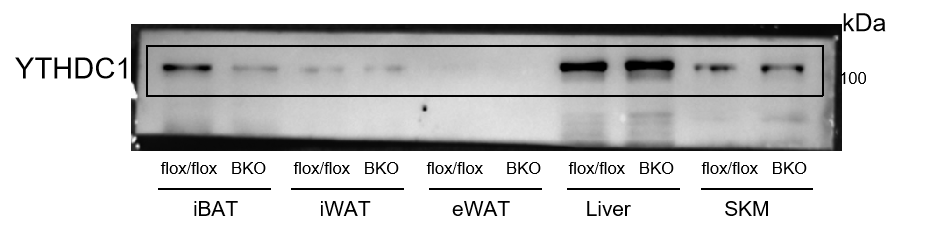

Supplement: Supplementary file 4 — Source data Fig. 1 [file 44318_2025_460_MOESM4_ESM.zip › Figure1/1D/western YTHDC1.tif]

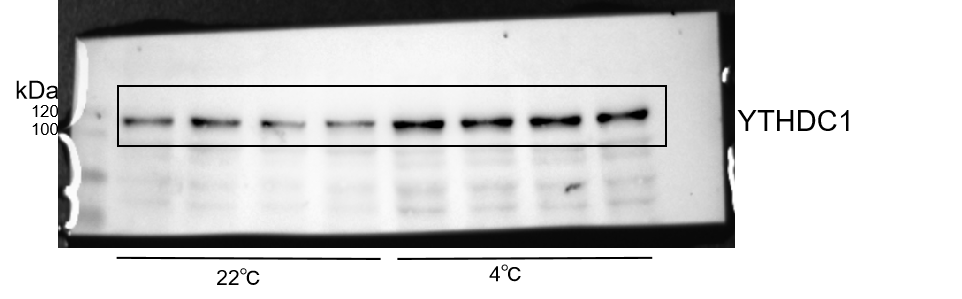

Supplement: Supplementary file 4 — Source data Fig. 1 [file 44318_2025_460_MOESM4_ESM.zip › Figure1/1C/western YTHDC1.tif]

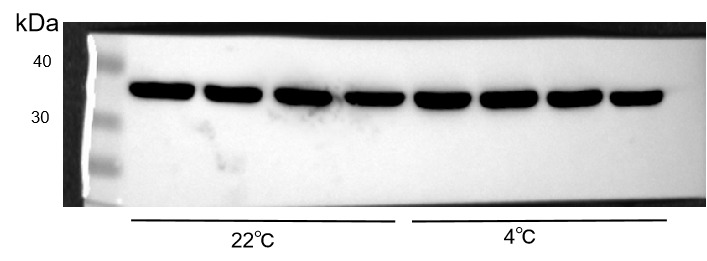

Supplement: Supplementary file 4 — Source data Fig. 1 [file 44318_2025_460_MOESM4_ESM.zip › Figure1/1C/western GAPDH.tif]

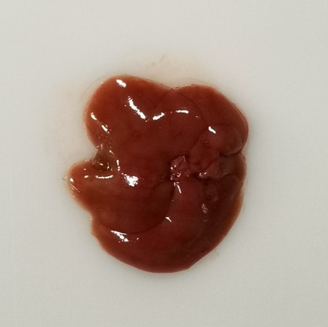

Supplement: Supplementary file 5 — Source data Fig. 2 [file 44318_2025_460_MOESM5_ESM.zip › Figure2/2G/FF LIVER.tif]

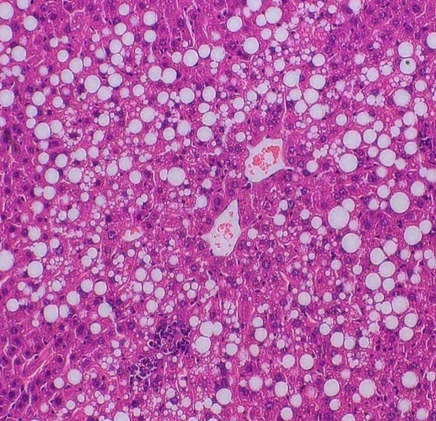

Supplement: Supplementary file 5 — Source data Fig. 2 [file 44318_2025_460_MOESM5_ESM.zip › Figure2/2G/BKO LIVER HE.tif]

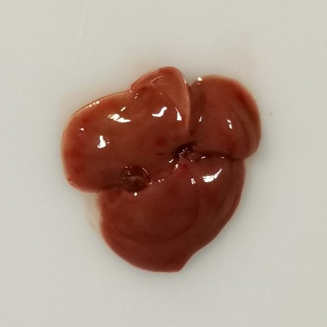

Supplement: Supplementary file 5 — Source data Fig. 2 [file 44318_2025_460_MOESM5_ESM.zip › Figure2/2G/BKO LIVER.tif]

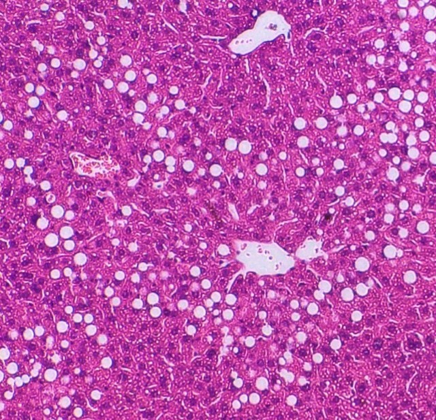

Supplement: Supplementary file 5 — Source data Fig. 2 [file 44318_2025_460_MOESM5_ESM.zip › Figure2/2G/FF LIVER HE.tif]

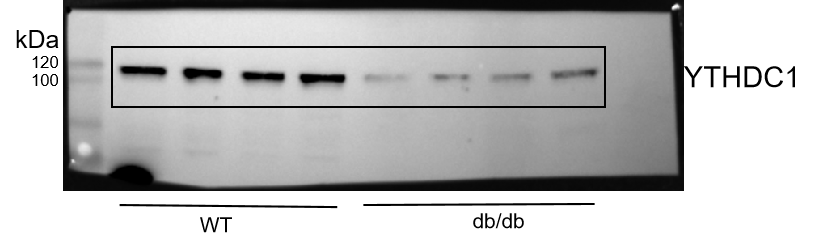

Supplement: Supplementary file 5 — Source data Fig. 2 [file 44318_2025_460_MOESM5_ESM.zip › Figure2/2A/western YTHDC1 db.tif]

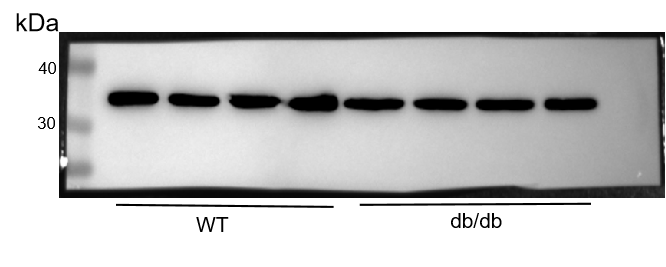

Supplement: Supplementary file 5 — Source data Fig. 2 [file 44318_2025_460_MOESM5_ESM.zip › Figure2/2A/western GAPDH db.tif]

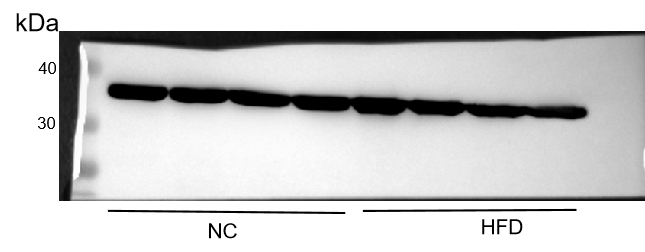

Supplement: Supplementary file 5 — Source data Fig. 2 [file 44318_2025_460_MOESM5_ESM.zip › Figure2/2A/western GAPDH HFD.tif]

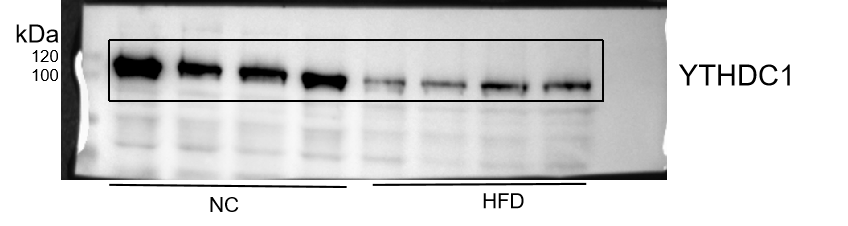

Supplement: Supplementary file 5 — Source data Fig. 2 [file 44318_2025_460_MOESM5_ESM.zip › Figure2/2A/western YTHDC1 HFD.tif]

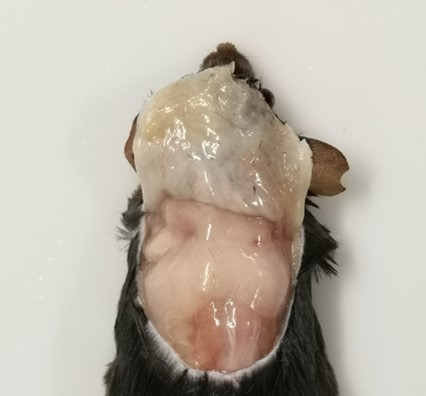

Supplement: Supplementary file 5 — Source data Fig. 2 [file 44318_2025_460_MOESM5_ESM.zip › Figure2/2C/FF Appearance.tif]

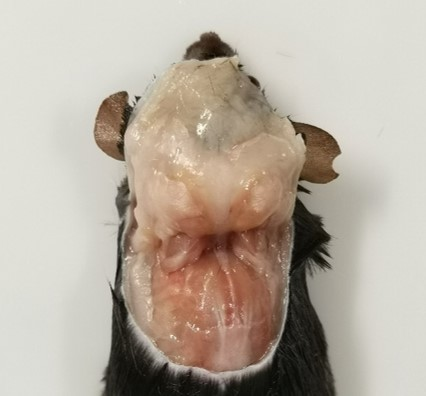

Supplement: Supplementary file 5 — Source data Fig. 2 [file 44318_2025_460_MOESM5_ESM.zip › Figure2/2C/FF Appearance flip.tif]

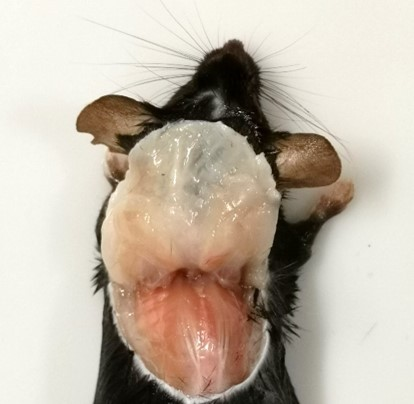

Supplement: Supplementary file 5 — Source data Fig. 2 [file 44318_2025_460_MOESM5_ESM.zip › Figure2/2C/BKO Appearance flip.tif]

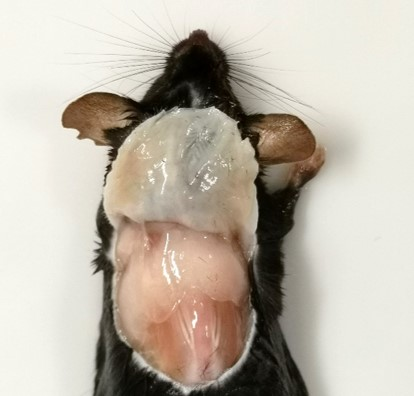

Supplement: Supplementary file 5 — Source data Fig. 2 [file 44318_2025_460_MOESM5_ESM.zip › Figure2/2C/BKO Appearance.tif]

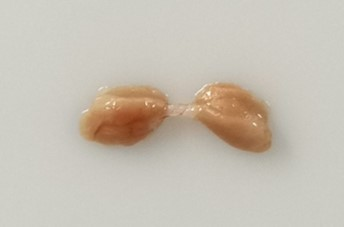

Supplement: Supplementary file 5 — Source data Fig. 2 [file 44318_2025_460_MOESM5_ESM.zip › Figure2/2D/FF iBAT.tif]

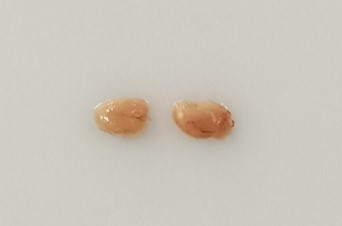

Supplement: Supplementary file 5 — Source data Fig. 2 [file 44318_2025_460_MOESM5_ESM.zip › Figure2/2D/BKO iBAT.tif]

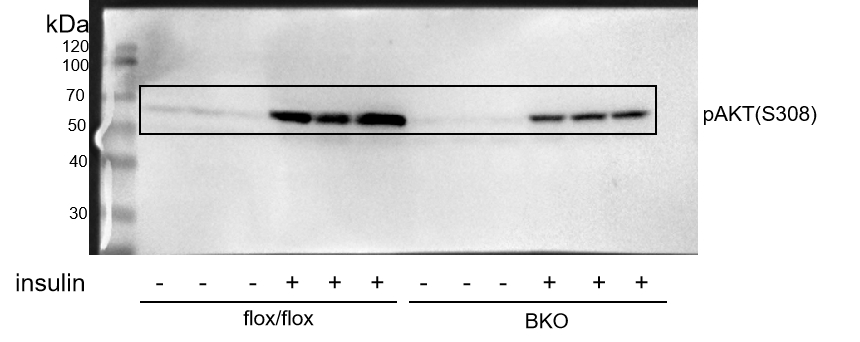

Supplement: Supplementary file 5 — Source data Fig. 2 [file 44318_2025_460_MOESM5_ESM.zip › Figure2/2K/western pAKT(S308).tif]

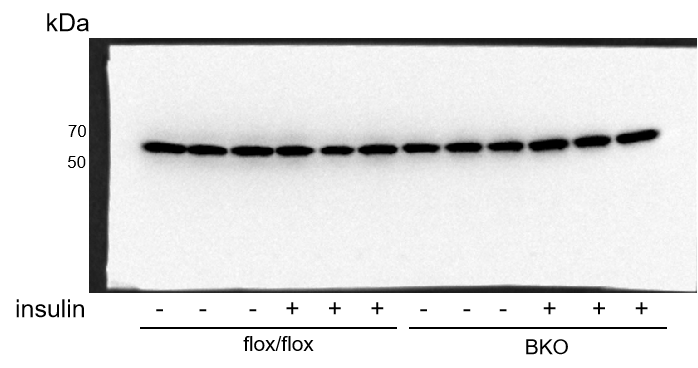

Supplement: Supplementary file 5 — Source data Fig. 2 [file 44318_2025_460_MOESM5_ESM.zip › Figure2/2K/western AKT.tif]

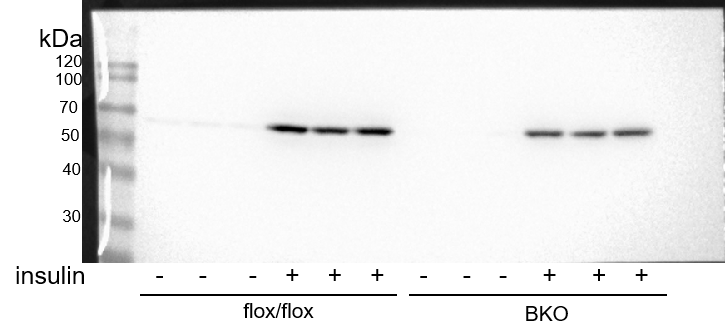

Supplement: Supplementary file 5 — Source data Fig. 2 [file 44318_2025_460_MOESM5_ESM.zip › Figure2/2K/western pAKT(S473).tif]

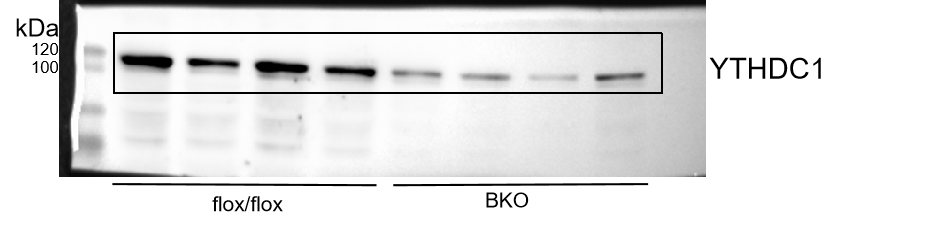

Supplement: Supplementary file 6 — Source data Fig. 3 [file 44318_2025_460_MOESM6_ESM.zip › Figure3/3F/western YTHDC1.tif]

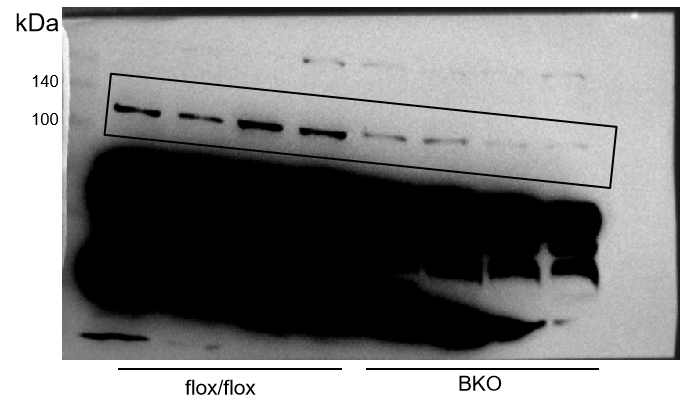

Supplement: Supplementary file 6 — Source data Fig. 3 [file 44318_2025_460_MOESM6_ESM.zip › Figure3/3F/western PRDM16.tif]

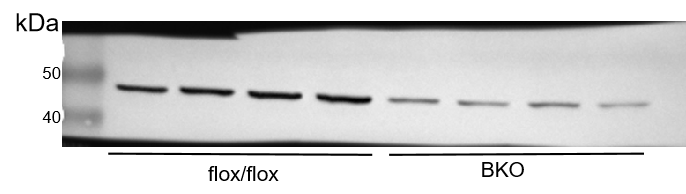

Supplement: Supplementary file 6 — Source data Fig. 3 [file 44318_2025_460_MOESM6_ESM.zip › Figure3/3F/western PPAR╬│.tif]

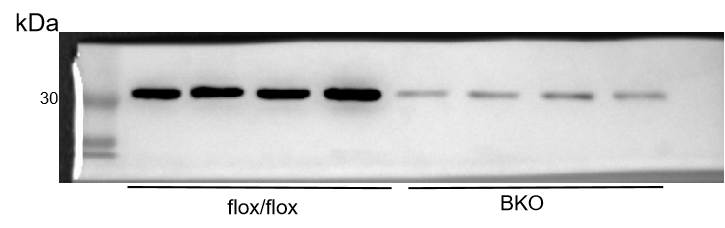

Supplement: Supplementary file 6 — Source data Fig. 3 [file 44318_2025_460_MOESM6_ESM.zip › Figure3/3F/western UCP1.tif]

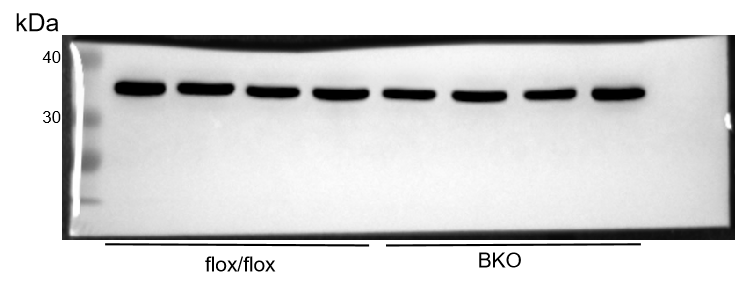

Supplement: Supplementary file 6 — Source data Fig. 3 [file 44318_2025_460_MOESM6_ESM.zip › Figure3/3F/western GAPDH.tif]

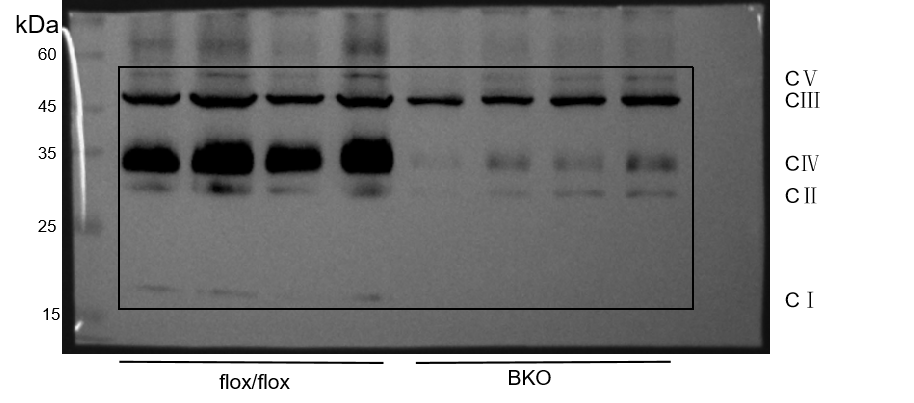

Supplement: Supplementary file 6 — Source data Fig. 3 [file 44318_2025_460_MOESM6_ESM.zip › Figure3/3G/western complexus.tif]

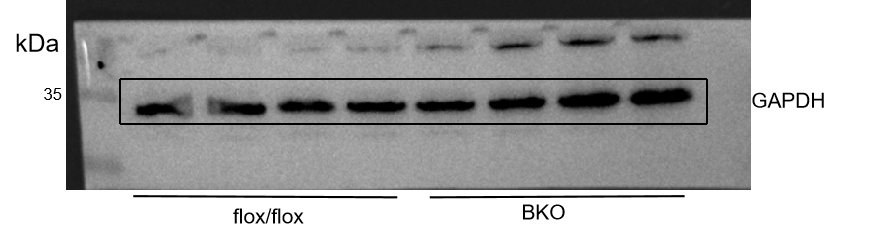

Supplement: Supplementary file 6 — Source data Fig. 3 [file 44318_2025_460_MOESM6_ESM.zip › Figure3/3G/western GAPDH.tif]

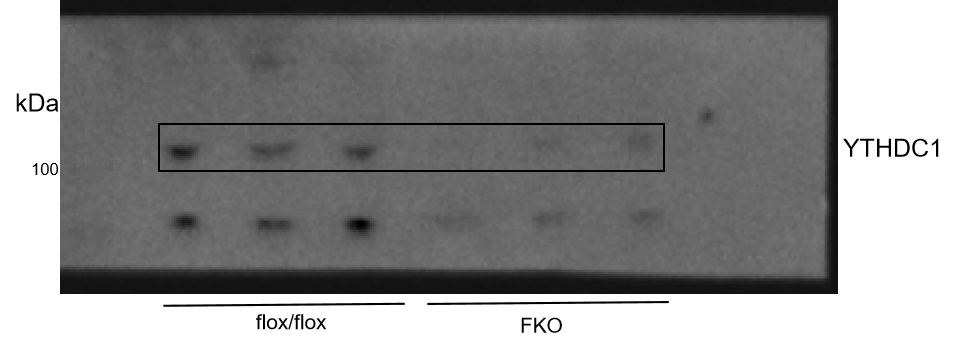

Supplement: Supplementary file 7 — Source data Fig. 4 [file 44318_2025_460_MOESM7_ESM.zip › Figure4/4E/western YTHDC1.tif]

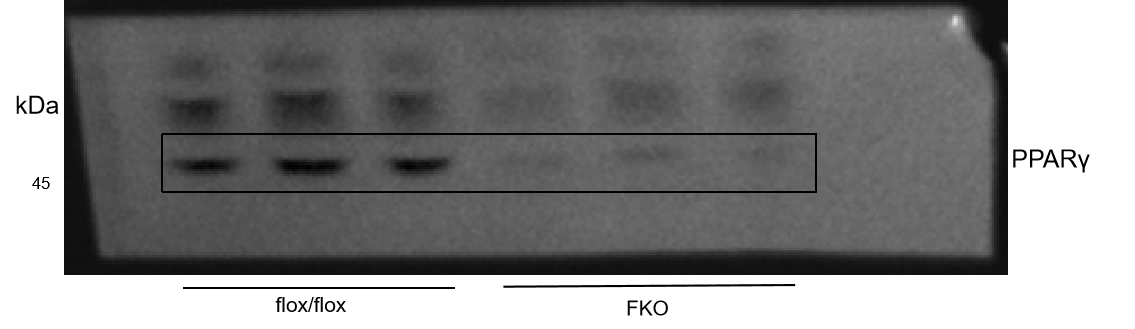

Supplement: Supplementary file 7 — Source data Fig. 4 [file 44318_2025_460_MOESM7_ESM.zip › Figure4/4E/western PPAR╬│.tif]

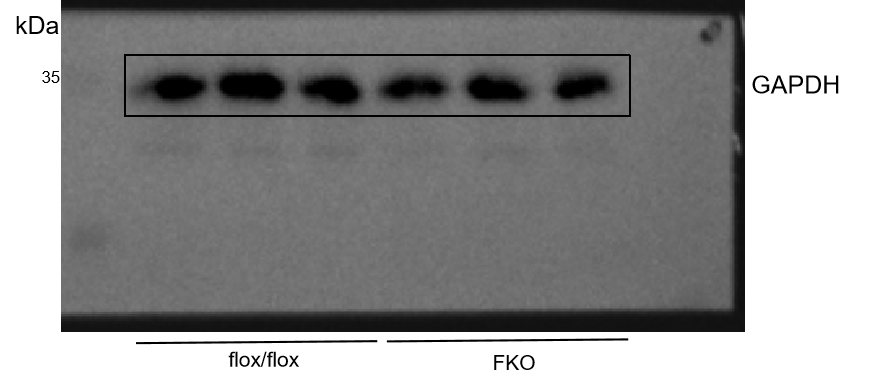

Supplement: Supplementary file 7 — Source data Fig. 4 [file 44318_2025_460_MOESM7_ESM.zip › Figure4/4E/western GAPDH.tif]

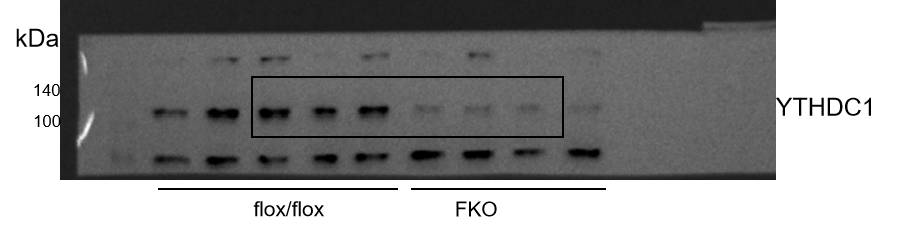

Supplement: Supplementary file 7 — Source data Fig. 4 [file 44318_2025_460_MOESM7_ESM.zip › Figure4/4C/western YTHDC1.tif]

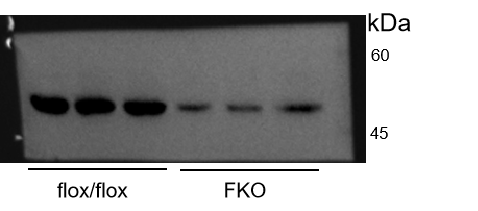

Supplement: Supplementary file 7 — Source data Fig. 4 [file 44318_2025_460_MOESM7_ESM.zip › Figure4/4C/western PPAR╬│.tif]

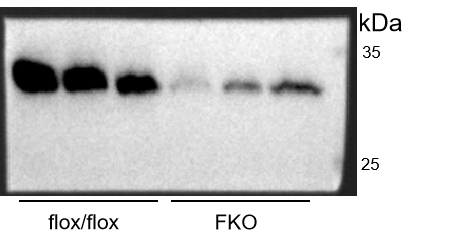

Supplement: Supplementary file 7 — Source data Fig. 4 [file 44318_2025_460_MOESM7_ESM.zip › Figure4/4C/western UCP1.tif]

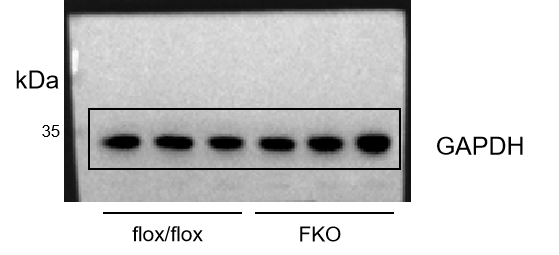

Supplement: Supplementary file 7 — Source data Fig. 4 [file 44318_2025_460_MOESM7_ESM.zip › Figure4/4C/western GAPDH.tif]

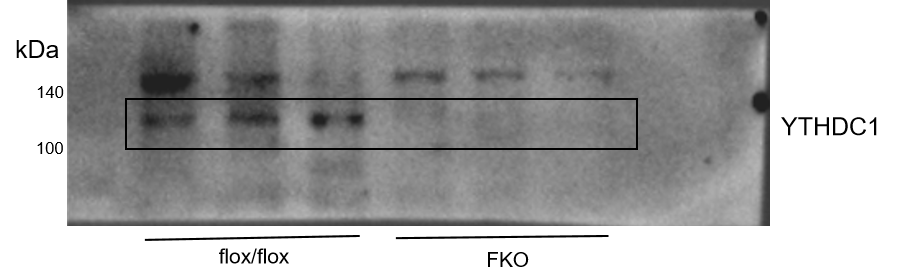

Supplement: Supplementary file 7 — Source data Fig. 4 [file 44318_2025_460_MOESM7_ESM.zip › Figure4/4D/western YTHDC1.tif]

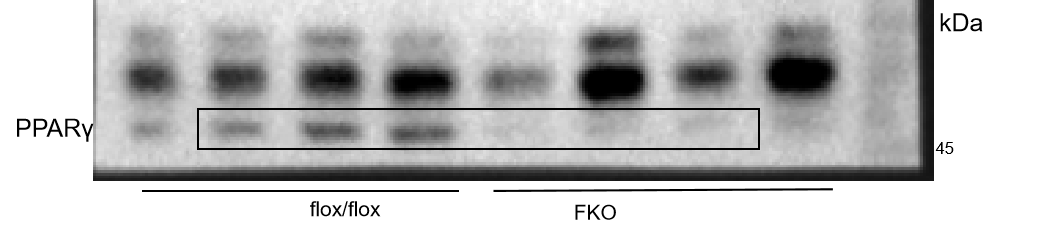

Supplement: Supplementary file 7 — Source data Fig. 4 [file 44318_2025_460_MOESM7_ESM.zip › Figure4/4D/western PPAR╬│.tif]

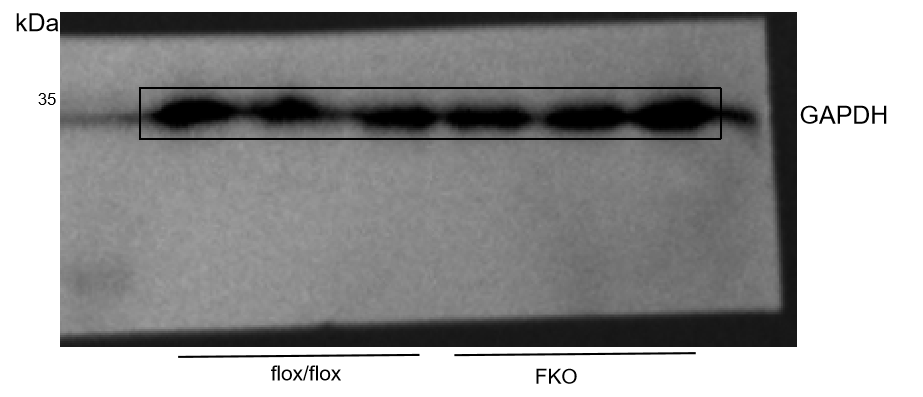

Supplement: Supplementary file 7 — Source data Fig. 4 [file 44318_2025_460_MOESM7_ESM.zip › Figure4/4D/western GAPDH.tif]

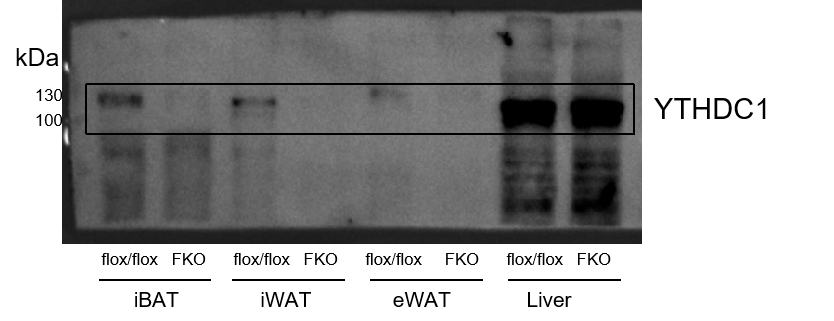

Supplement: Supplementary file 7 — Source data Fig. 4 [file 44318_2025_460_MOESM7_ESM.zip › Figure4/4A/western YTHDC1.tif]

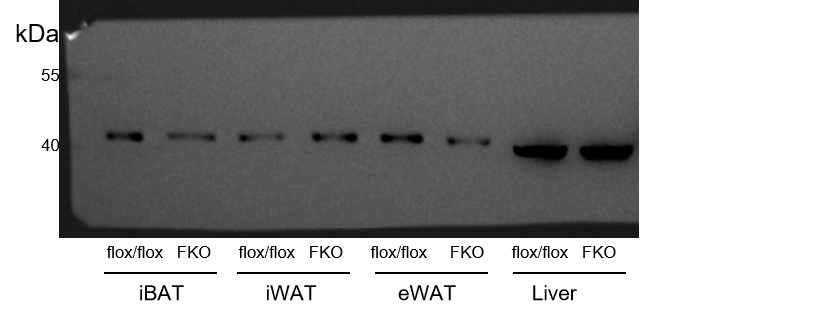

Supplement: Supplementary file 7 — Source data Fig. 4 [file 44318_2025_460_MOESM7_ESM.zip › Figure4/4A/western ╬▓-actin.tif]

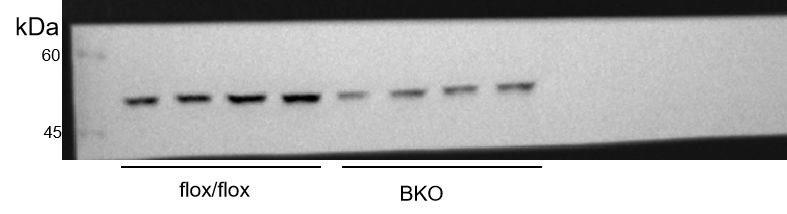

Supplement: Supplementary file 8 — Source data Fig. 5 [file 44318_2025_460_MOESM8_ESM.zip › Figure5/5A/western PPAR╬│.tif]

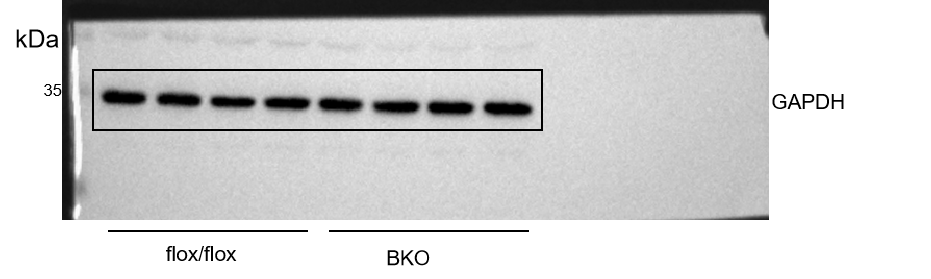

Supplement: Supplementary file 8 — Source data Fig. 5 [file 44318_2025_460_MOESM8_ESM.zip › Figure5/5A/western GAPDH.tif]

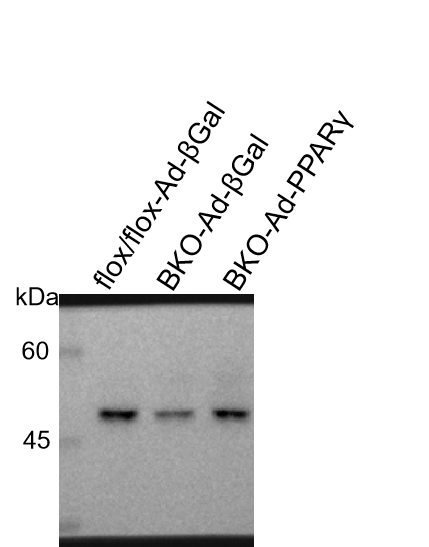

Supplement: Supplementary file 8 — Source data Fig. 5 [file 44318_2025_460_MOESM8_ESM.zip › Figure5/5E/western PPAR╬│.tif]

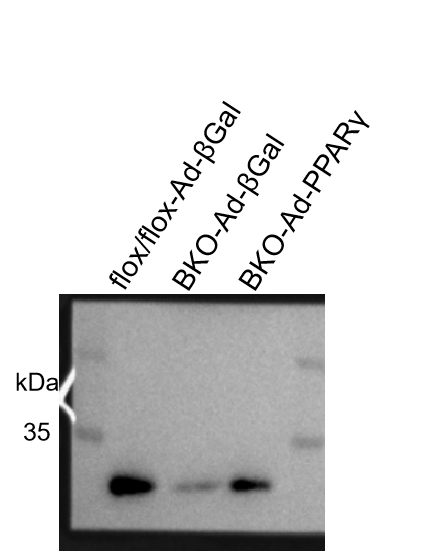

Supplement: Supplementary file 8 — Source data Fig. 5 [file 44318_2025_460_MOESM8_ESM.zip › Figure5/5E/western UCP1.tif]

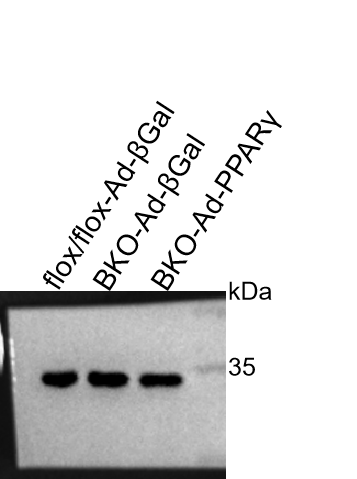

Supplement: Supplementary file 8 — Source data Fig. 5 [file 44318_2025_460_MOESM8_ESM.zip › Figure5/5E/western GAPDH.tif]

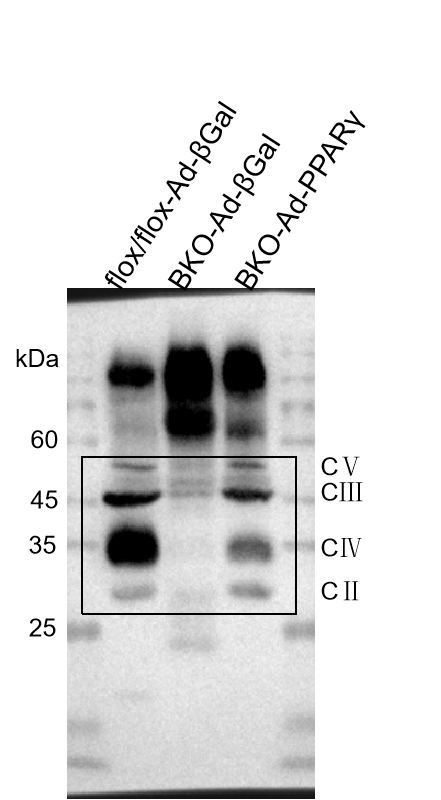

Supplement: Supplementary file 8 — Source data Fig. 5 [file 44318_2025_460_MOESM8_ESM.zip › Figure5/5E/western OXPHOS.tif]

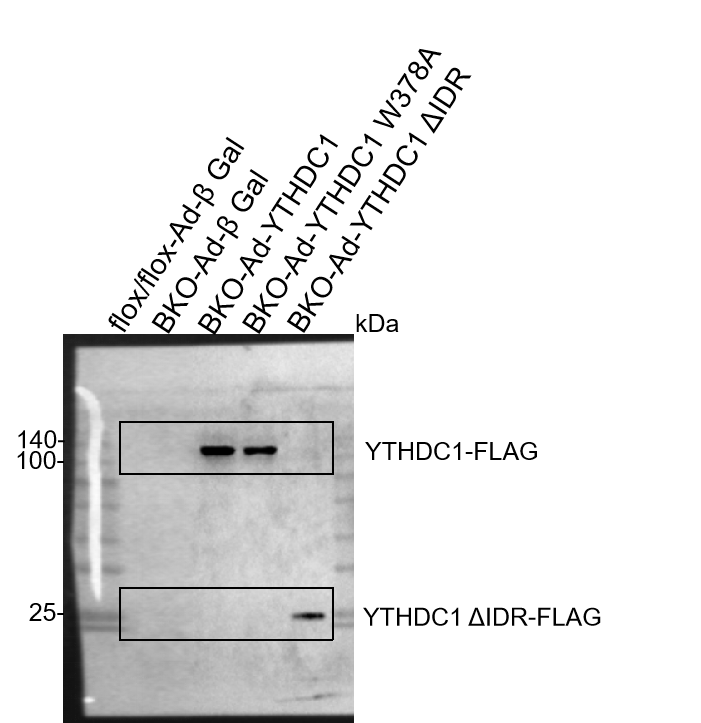

Supplement: Supplementary file 9 — Source data Fig. 6 [file 44318_2025_460_MOESM9_ESM.zip › Figure6/6D/western FLAG.tif]

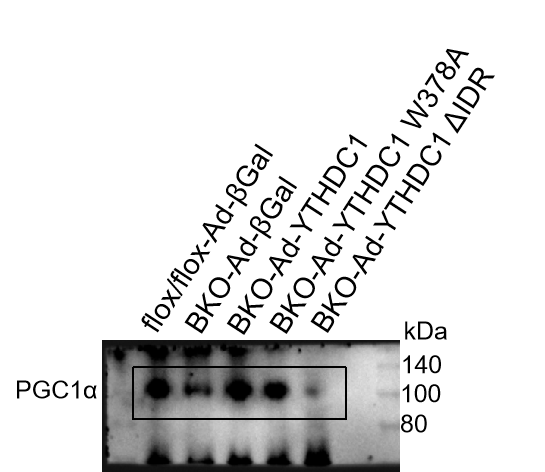

Supplement: Supplementary file 9 — Source data Fig. 6 [file 44318_2025_460_MOESM9_ESM.zip › Figure6/6D/western PGC1╬▒.tif]

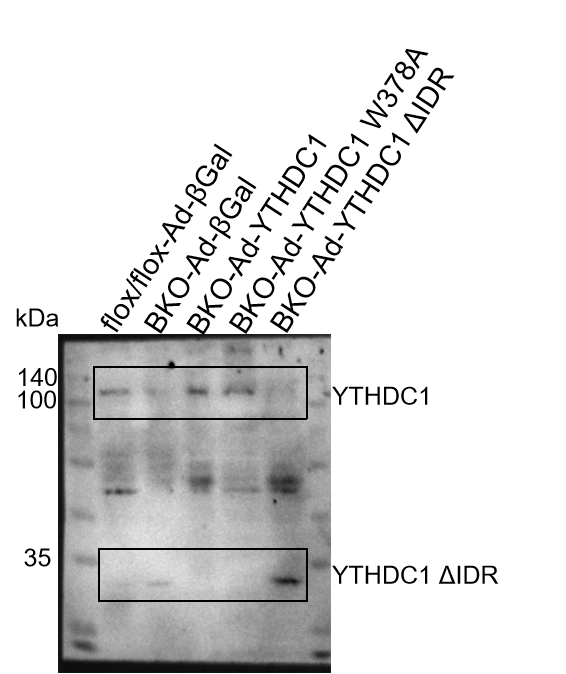

Supplement: Supplementary file 9 — Source data Fig. 6 [file 44318_2025_460_MOESM9_ESM.zip › Figure6/6D/western YTHDC1.tif]

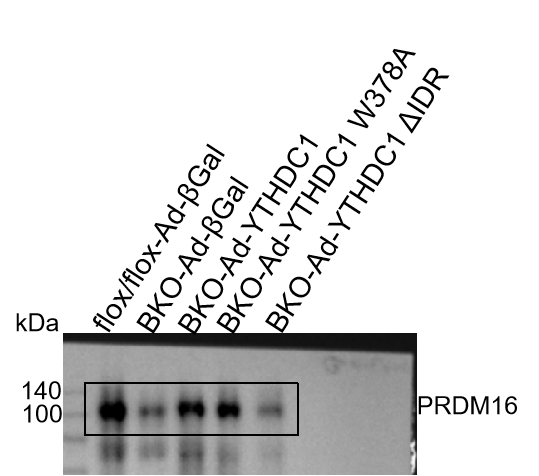

Supplement: Supplementary file 9 — Source data Fig. 6 [file 44318_2025_460_MOESM9_ESM.zip › Figure6/6D/western PRDM16.tif]

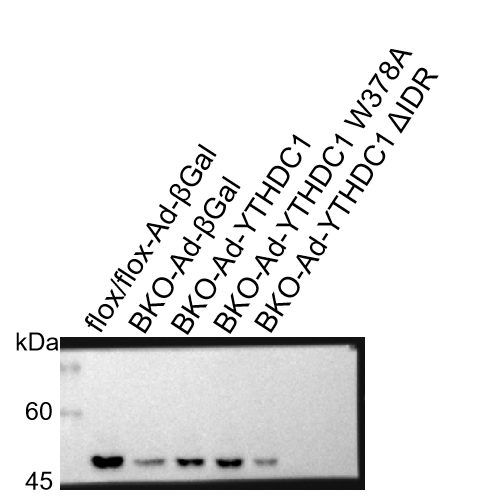

Supplement: Supplementary file 9 — Source data Fig. 6 [file 44318_2025_460_MOESM9_ESM.zip › Figure6/6D/western PPAR╬│.tif]

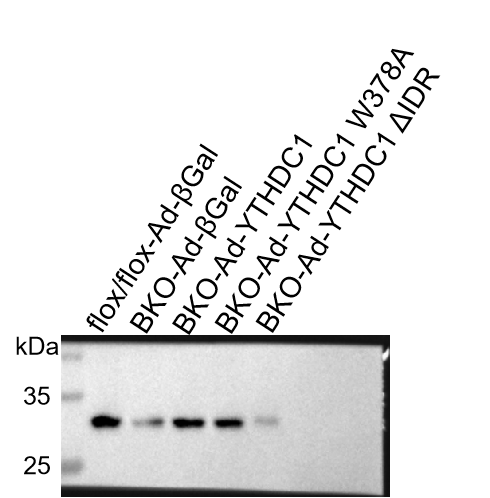

Supplement: Supplementary file 9 — Source data Fig. 6 [file 44318_2025_460_MOESM9_ESM.zip › Figure6/6D/western UCP1.tif]

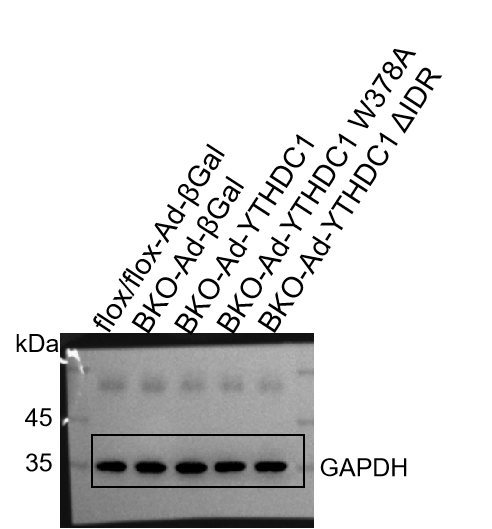

Supplement: Supplementary file 9 — Source data Fig. 6 [file 44318_2025_460_MOESM9_ESM.zip › Figure6/6D/western GAPDH.tif]

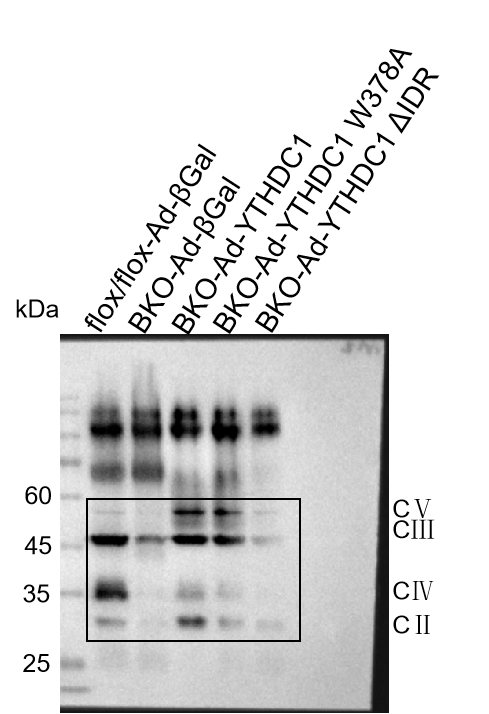

Supplement: Supplementary file 9 — Source data Fig. 6 [file 44318_2025_460_MOESM9_ESM.zip › Figure6/6D/western OXPHOS.tif]

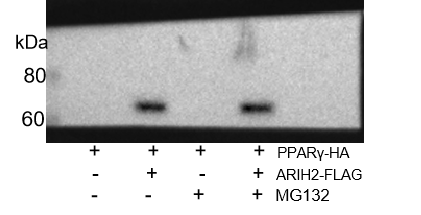

Supplement: Supplementary file 10 — Source data Fig. 7 [file 44318_2025_460_MOESM10_ESM.zip › Figure7/7J/western IP FLAG.tif]

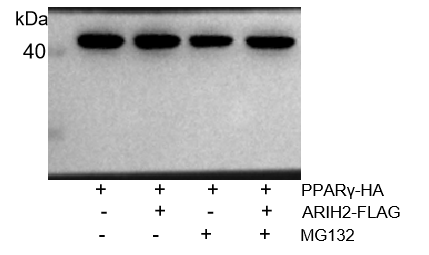

Supplement: Supplementary file 10 — Source data Fig. 7 [file 44318_2025_460_MOESM10_ESM.zip › Figure7/7J/western actin.tif]

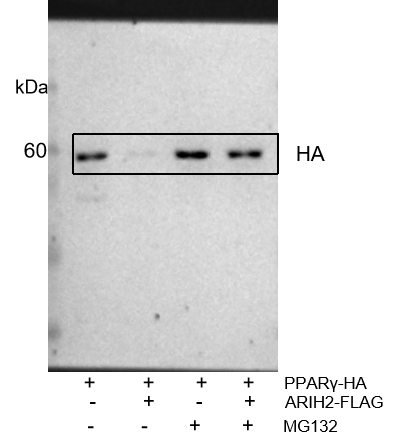

Supplement: Supplementary file 10 — Source data Fig. 7 [file 44318_2025_460_MOESM10_ESM.zip › Figure7/7J/western input HA.tif]

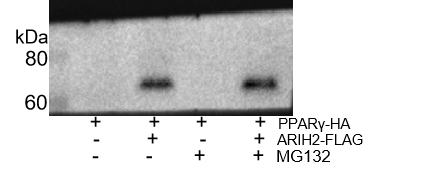

Supplement: Supplementary file 10 — Source data Fig. 7 [file 44318_2025_460_MOESM10_ESM.zip › Figure7/7J/western input FLAG.tif]

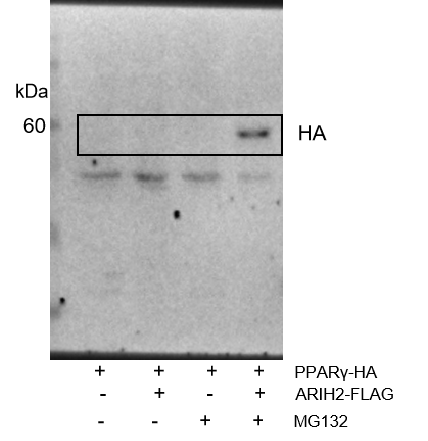

Supplement: Supplementary file 10 — Source data Fig. 7 [file 44318_2025_460_MOESM10_ESM.zip › Figure7/7J/western IP HA.tif]

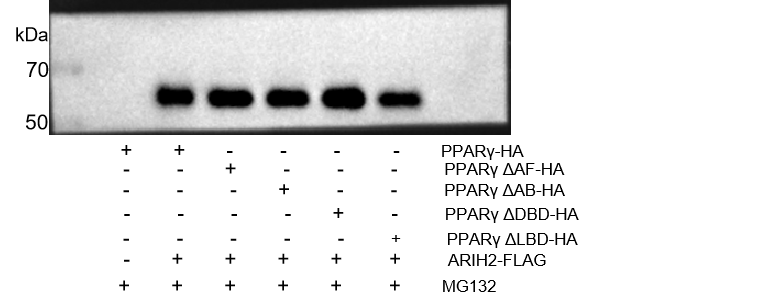

Supplement: Supplementary file 10 — Source data Fig. 7 [file 44318_2025_460_MOESM10_ESM.zip › Figure7/7M/western IP FLAG.tif]

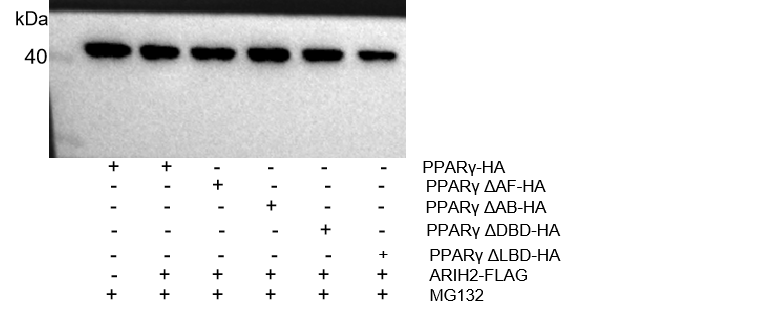

Supplement: Supplementary file 10 — Source data Fig. 7 [file 44318_2025_460_MOESM10_ESM.zip › Figure7/7M/western actin.tif]

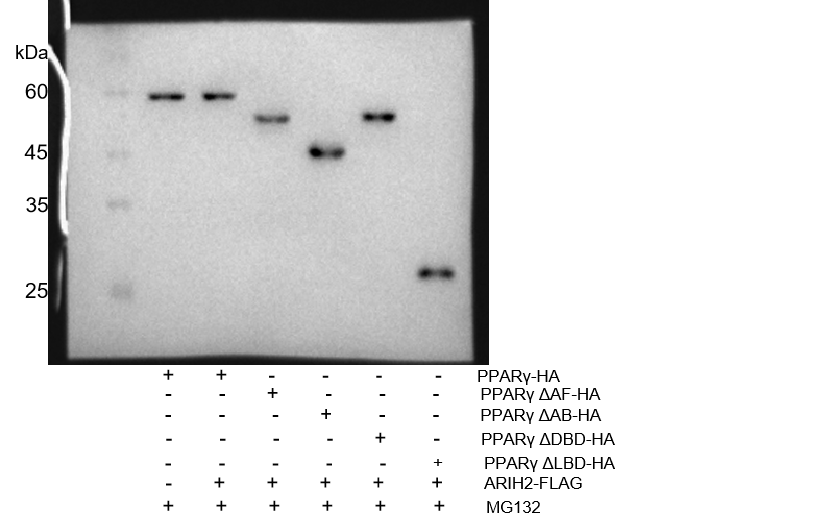

Supplement: Supplementary file 10 — Source data Fig. 7 [file 44318_2025_460_MOESM10_ESM.zip › Figure7/7M/western input HA.tif]

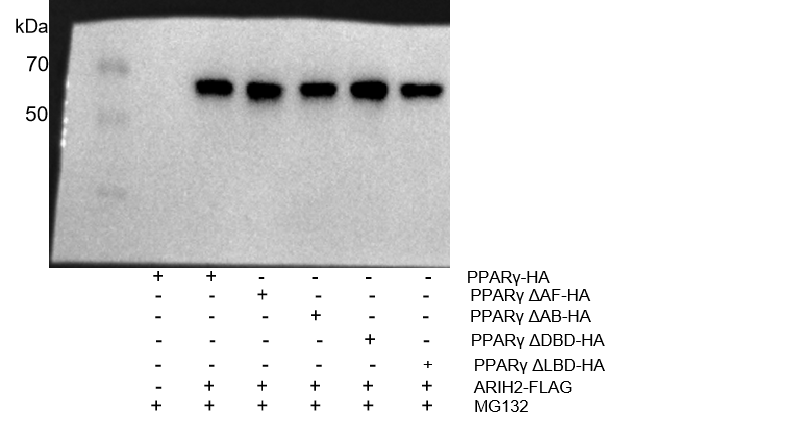

Supplement: Supplementary file 10 — Source data Fig. 7 [file 44318_2025_460_MOESM10_ESM.zip › Figure7/7M/western input FLAG.tif]

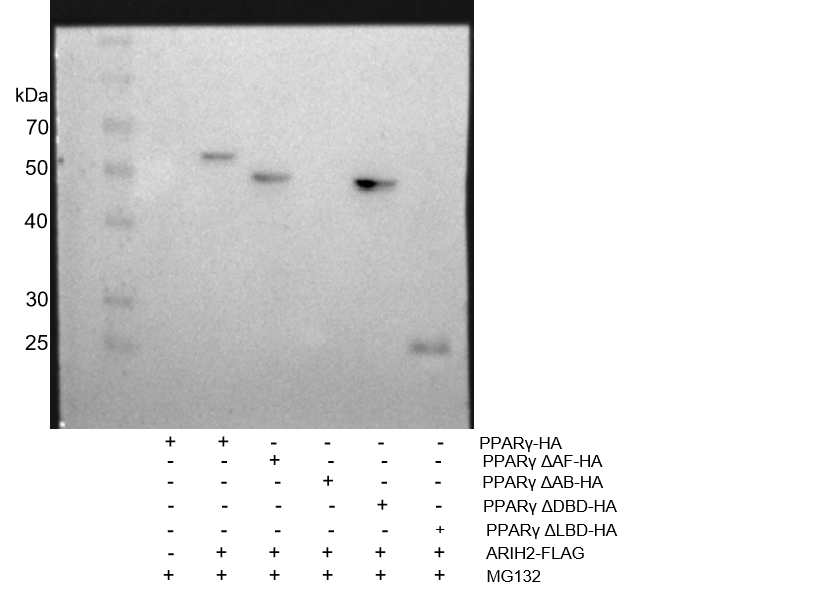

Supplement: Supplementary file 10 — Source data Fig. 7 [file 44318_2025_460_MOESM10_ESM.zip › Figure7/7M/western IP HA.tif]

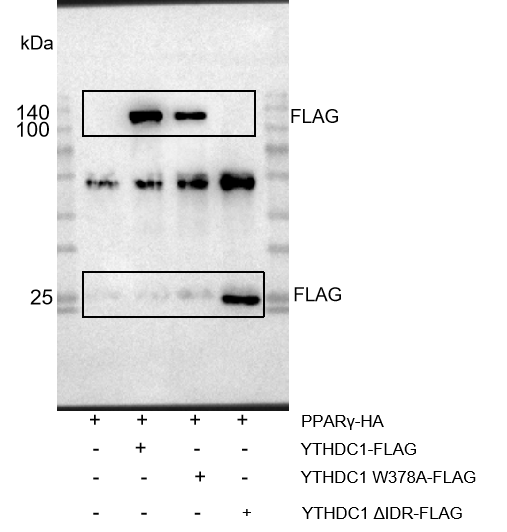

Supplement: Supplementary file 10 — Source data Fig. 7 [file 44318_2025_460_MOESM10_ESM.zip › Figure7/7D/western IP FLAG.tif]

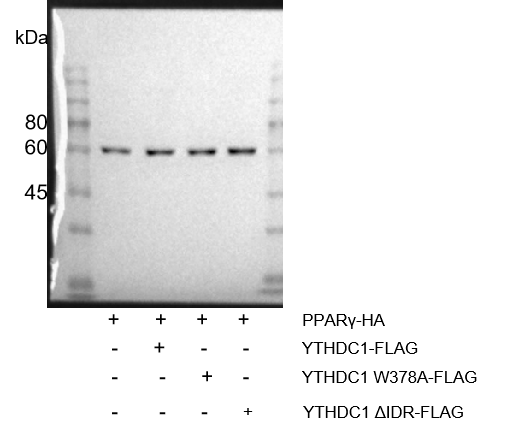

Supplement: Supplementary file 10 — Source data Fig. 7 [file 44318_2025_460_MOESM10_ESM.zip › Figure7/7D/western Input HA.tif]

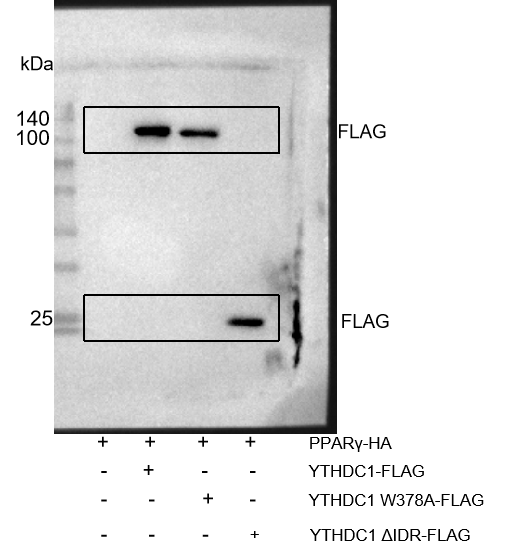

Supplement: Supplementary file 10 — Source data Fig. 7 [file 44318_2025_460_MOESM10_ESM.zip › Figure7/7D/western Input FLAG.tif]

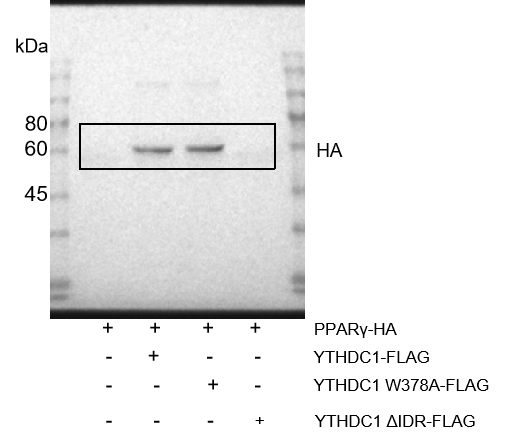

Supplement: Supplementary file 10 — Source data Fig. 7 [file 44318_2025_460_MOESM10_ESM.zip › Figure7/7D/western IP HA.tif]

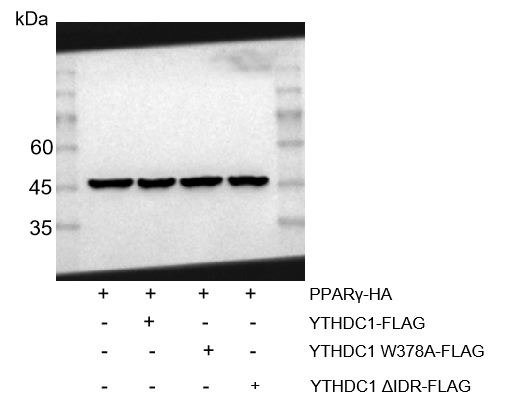

Supplement: Supplementary file 10 — Source data Fig. 7 [file 44318_2025_460_MOESM10_ESM.zip › Figure7/7D/western Input actin.tif]

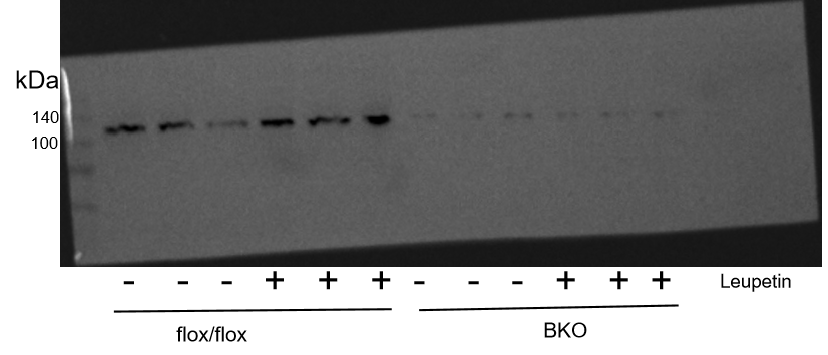

Supplement: Supplementary file 10 — Source data Fig. 7 [file 44318_2025_460_MOESM10_ESM.zip › Figure7/7C/western YTHDC1.tif]

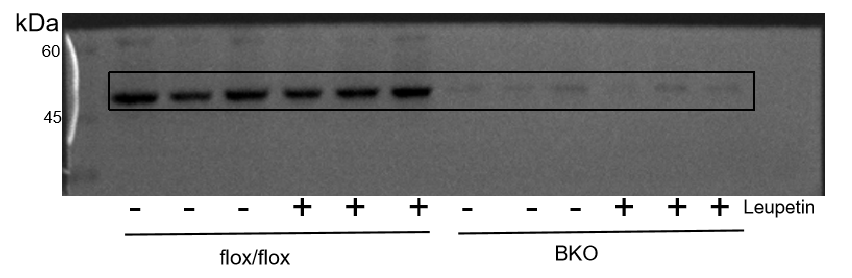

Supplement: Supplementary file 10 — Source data Fig. 7 [file 44318_2025_460_MOESM10_ESM.zip › Figure7/7C/western PPAR╬│.tif]

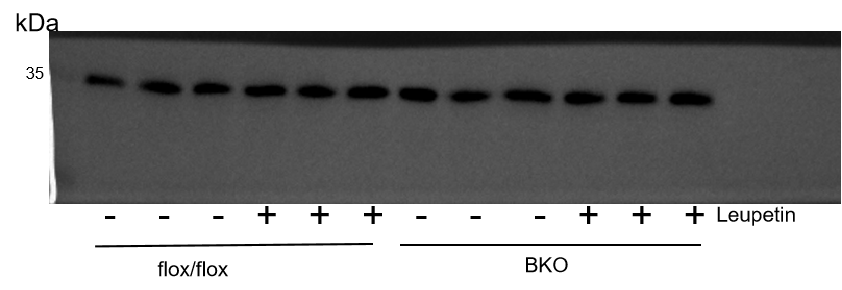

Supplement: Supplementary file 10 — Source data Fig. 7 [file 44318_2025_460_MOESM10_ESM.zip › Figure7/7C/western GAPDH.tif]

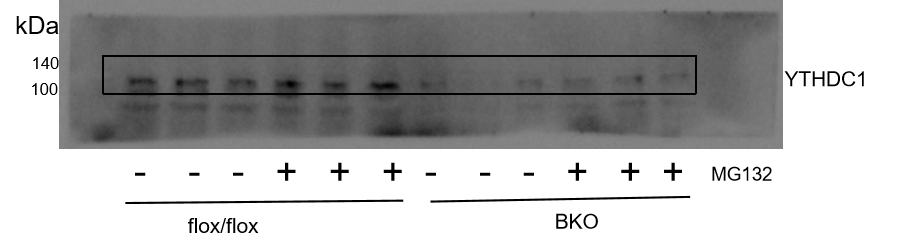

Supplement: Supplementary file 10 — Source data Fig. 7 [file 44318_2025_460_MOESM10_ESM.zip › Figure7/7B/western YTHDC1.tif]

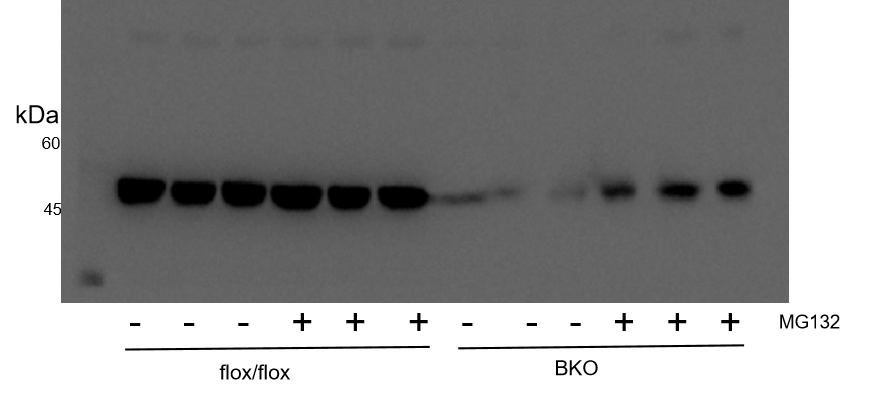

Supplement: Supplementary file 10 — Source data Fig. 7 [file 44318_2025_460_MOESM10_ESM.zip › Figure7/7B/western PPAR╬│.tif]

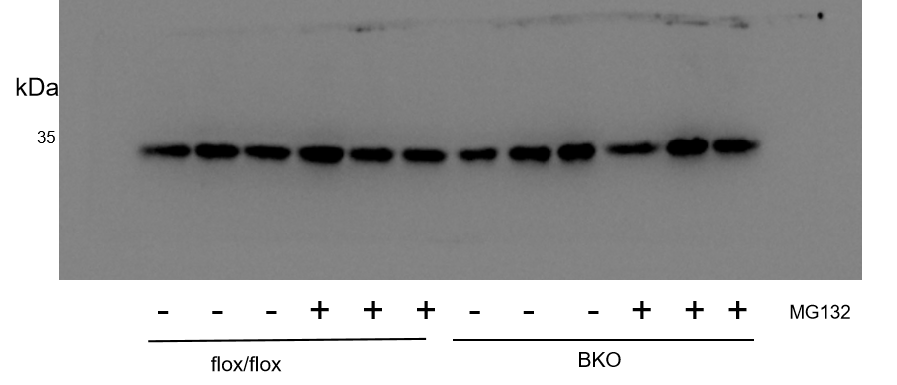

Supplement: Supplementary file 10 — Source data Fig. 7 [file 44318_2025_460_MOESM10_ESM.zip › Figure7/7B/western GAPDH.tif]

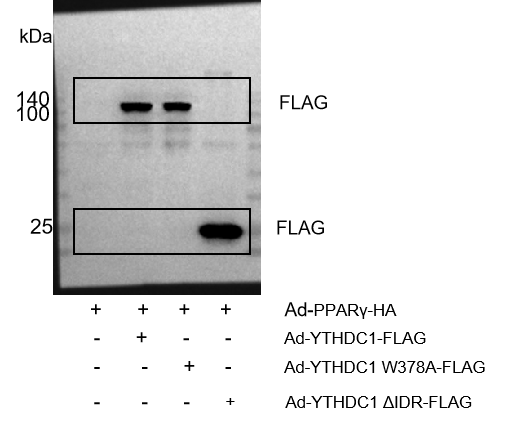

Supplement: Supplementary file 10 — Source data Fig. 7 [file 44318_2025_460_MOESM10_ESM.zip › Figure7/7E/western IP FLAG.tif]

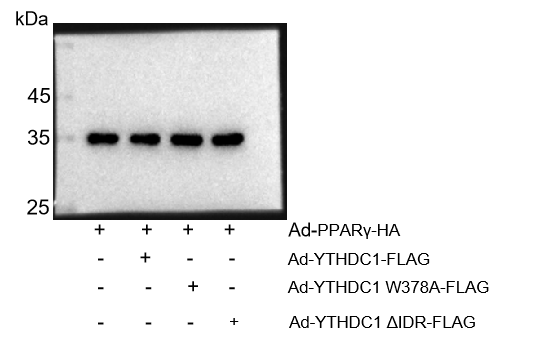

Supplement: Supplementary file 10 — Source data Fig. 7 [file 44318_2025_460_MOESM10_ESM.zip › Figure7/7E/western Input GAPDH.tif]

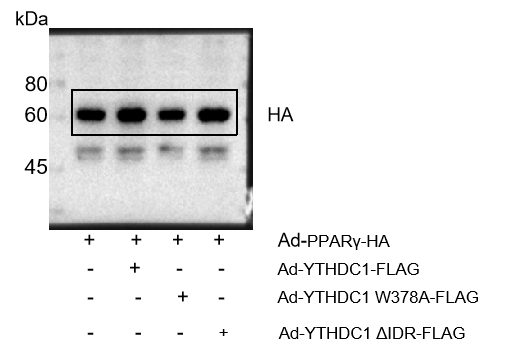

Supplement: Supplementary file 10 — Source data Fig. 7 [file 44318_2025_460_MOESM10_ESM.zip › Figure7/7E/western Input HA.tif]

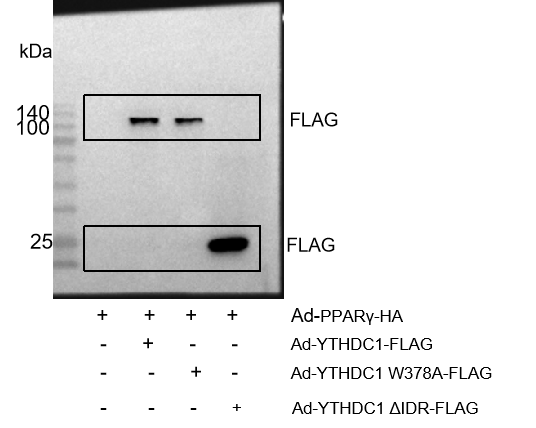

Supplement: Supplementary file 10 — Source data Fig. 7 [file 44318_2025_460_MOESM10_ESM.zip › Figure7/7E/western Input FLAG.tif]

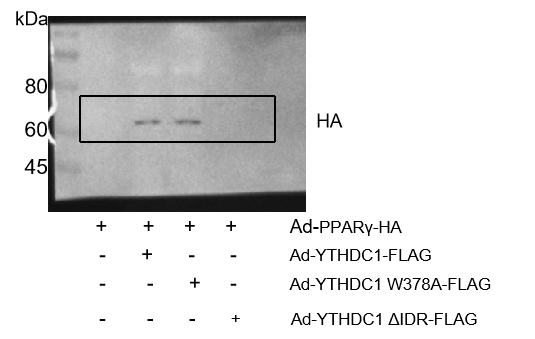

Supplement: Supplementary file 10 — Source data Fig. 7 [file 44318_2025_460_MOESM10_ESM.zip › Figure7/7E/western IP HA.tif]

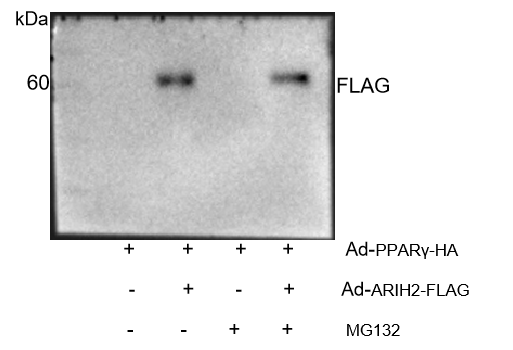

Supplement: Supplementary file 10 — Source data Fig. 7 [file 44318_2025_460_MOESM10_ESM.zip › Figure7/7K/western IP FLAG.tif]

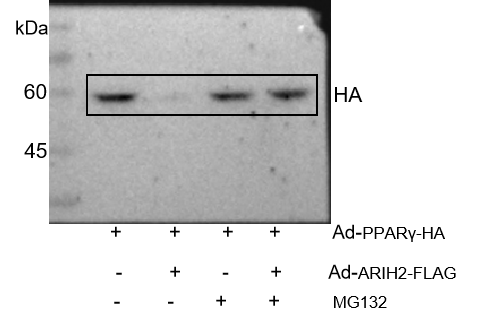

Supplement: Supplementary file 10 — Source data Fig. 7 [file 44318_2025_460_MOESM10_ESM.zip › Figure7/7K/western input HA.tif]

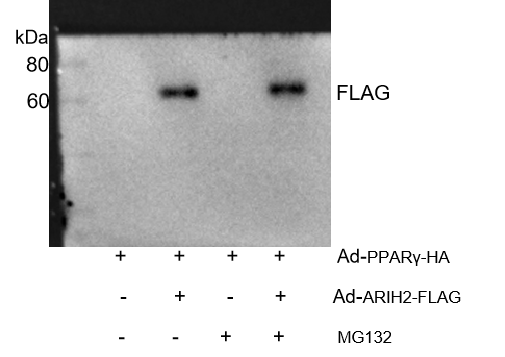

Supplement: Supplementary file 10 — Source data Fig. 7 [file 44318_2025_460_MOESM10_ESM.zip › Figure7/7K/western input FLAG.tif]

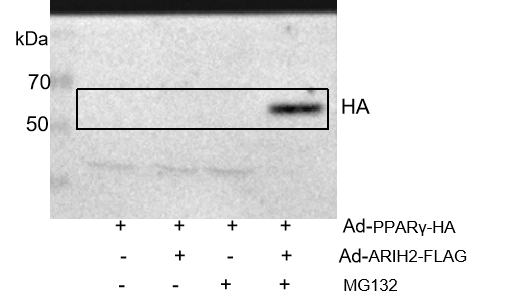

Supplement: Supplementary file 10 — Source data Fig. 7 [file 44318_2025_460_MOESM10_ESM.zip › Figure7/7K/western IP HA.tif]

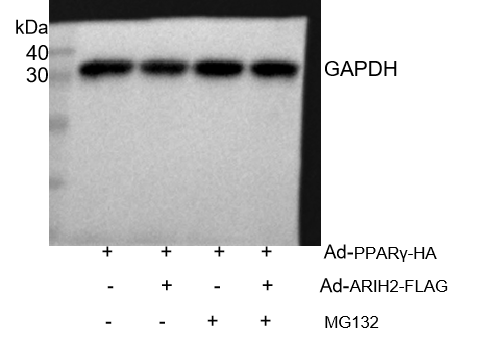

Supplement: Supplementary file 10 — Source data Fig. 7 [file 44318_2025_460_MOESM10_ESM.zip › Figure7/7K/western GAPDH.tif]

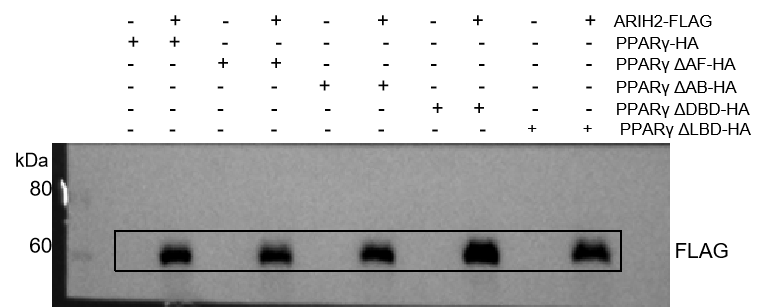

Supplement: Supplementary file 10 — Source data Fig. 7 [file 44318_2025_460_MOESM10_ESM.zip › Figure7/7N/western FLAG.tif]

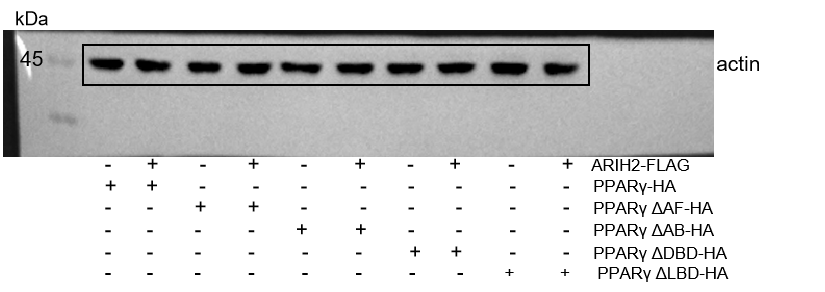

Supplement: Supplementary file 10 — Source data Fig. 7 [file 44318_2025_460_MOESM10_ESM.zip › Figure7/7N/western actin.tif]

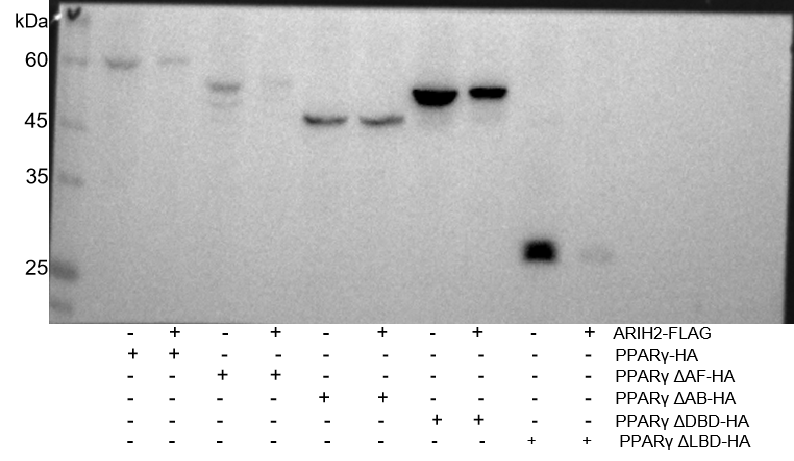

Supplement: Supplementary file 10 — Source data Fig. 7 [file 44318_2025_460_MOESM10_ESM.zip › Figure7/7N/western HA.tif]

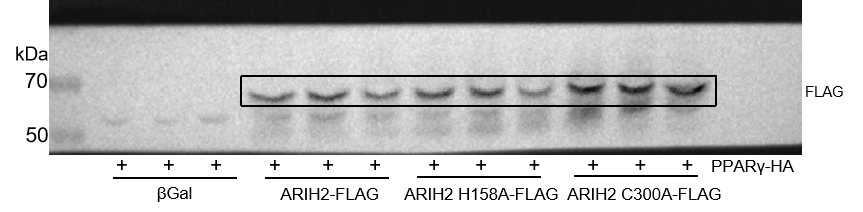

Supplement: Supplementary file 10 — Source data Fig. 7 [file 44318_2025_460_MOESM10_ESM.zip › Figure7/7I/western FLAG.tif]

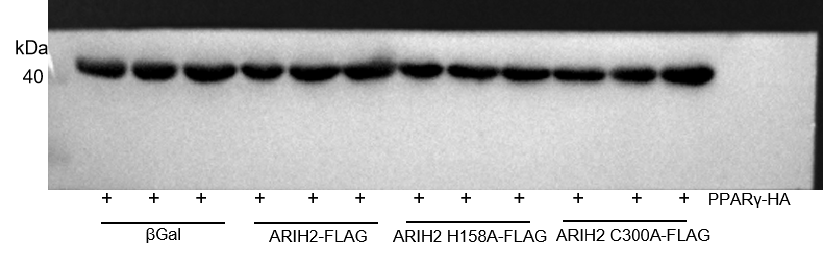

Supplement: Supplementary file 10 — Source data Fig. 7 [file 44318_2025_460_MOESM10_ESM.zip › Figure7/7I/western actin.tif]
