# Supplementary figures and images for: YTHDC1 promotes postnatal brown adipose tissue development and thermogenesis by stabilizing PPARγ (part 2 of 3)
Source: EMBO J. 2025 May 12;44(12):3360–80. doi: 10.1038/s44318-025-00460-x (PMC12170836; doi:10.1038/s44318-025-00460-x)

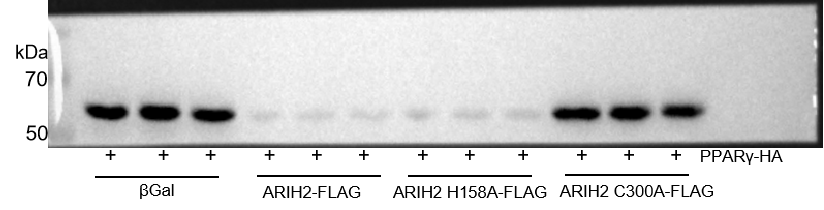

Supplement: Supplementary file 10 — Source data Fig. 7 [file 44318_2025_460_MOESM10_ESM.zip › Figure7/7I/western HA.tif]

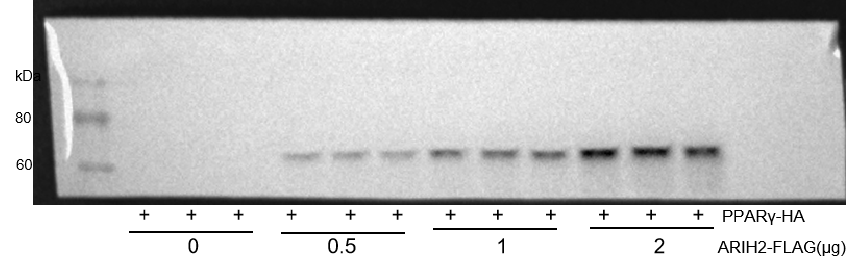

Supplement: Supplementary file 10 — Source data Fig. 7 [file 44318_2025_460_MOESM10_ESM.zip › Figure7/7G/western FLAG.tif]

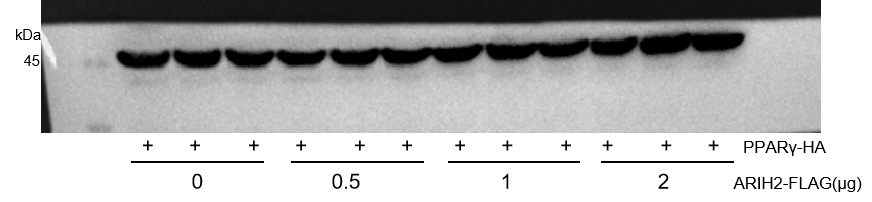

Supplement: Supplementary file 10 — Source data Fig. 7 [file 44318_2025_460_MOESM10_ESM.zip › Figure7/7G/western actin.tif]

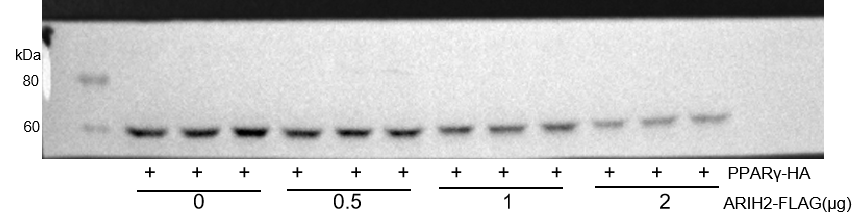

Supplement: Supplementary file 10 — Source data Fig. 7 [file 44318_2025_460_MOESM10_ESM.zip › Figure7/7G/western HA.tif]

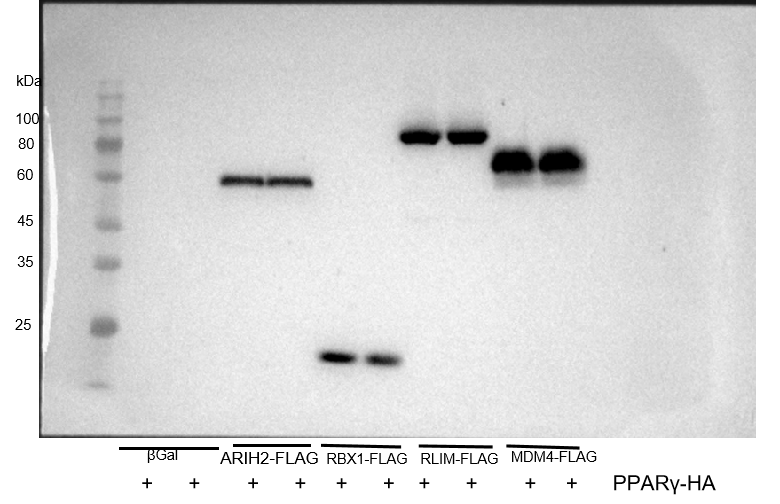

Supplement: Supplementary file 10 — Source data Fig. 7 [file 44318_2025_460_MOESM10_ESM.zip › Figure7/7F/western FLAG.tif]

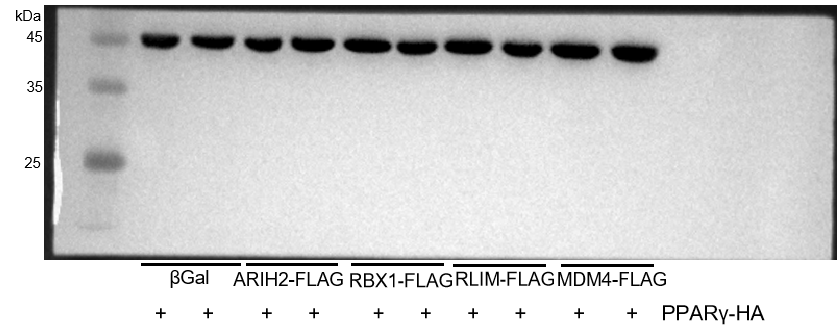

Supplement: Supplementary file 10 — Source data Fig. 7 [file 44318_2025_460_MOESM10_ESM.zip › Figure7/7F/western actin.tif]

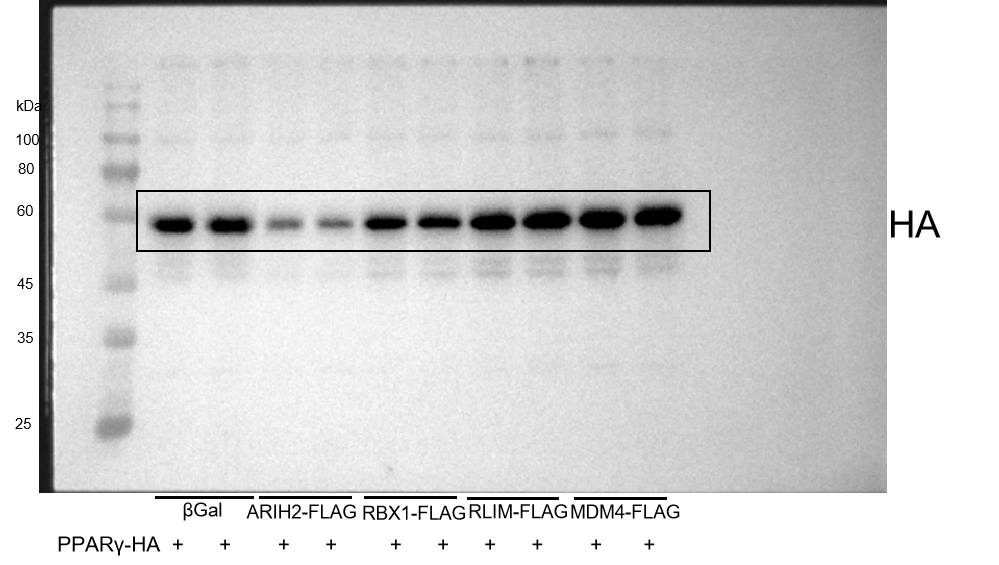

Supplement: Supplementary file 10 — Source data Fig. 7 [file 44318_2025_460_MOESM10_ESM.zip › Figure7/7F/western HA.tif]

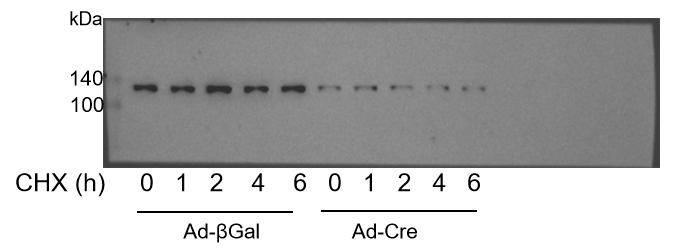

Supplement: Supplementary file 10 — Source data Fig. 7 [file 44318_2025_460_MOESM10_ESM.zip › Figure7/7A/western YTHDC1.tif]

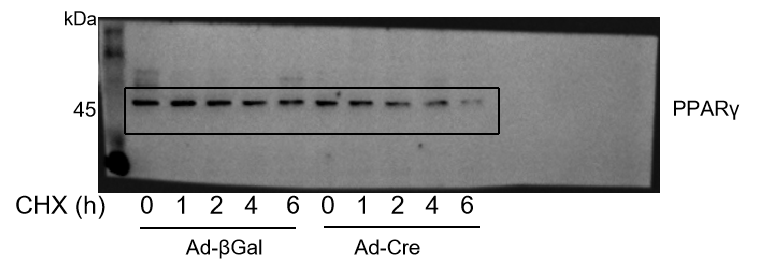

Supplement: Supplementary file 10 — Source data Fig. 7 [file 44318_2025_460_MOESM10_ESM.zip › Figure7/7A/western PPAR╬│.tif]

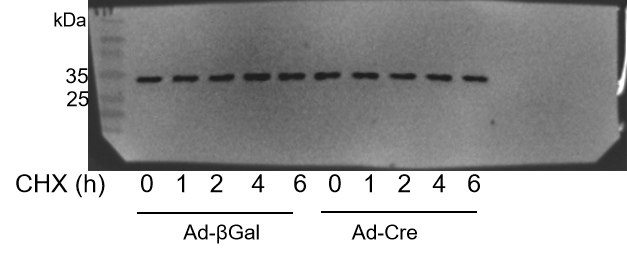

Supplement: Supplementary file 10 — Source data Fig. 7 [file 44318_2025_460_MOESM10_ESM.zip › Figure7/7A/western GAPDH.tif]

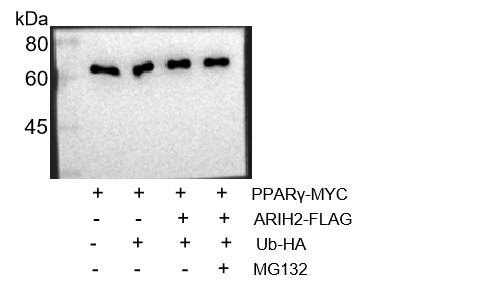

Supplement: Supplementary file 10 — Source data Fig. 7 [file 44318_2025_460_MOESM10_ESM.zip › Figure7/7O/western IP MYC.tif]

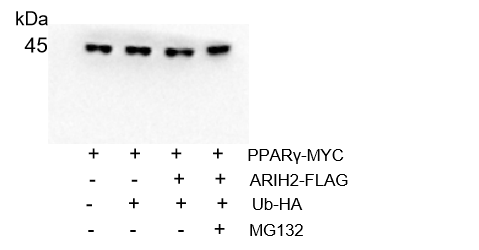

Supplement: Supplementary file 10 — Source data Fig. 7 [file 44318_2025_460_MOESM10_ESM.zip › Figure7/7O/western actin.tif]

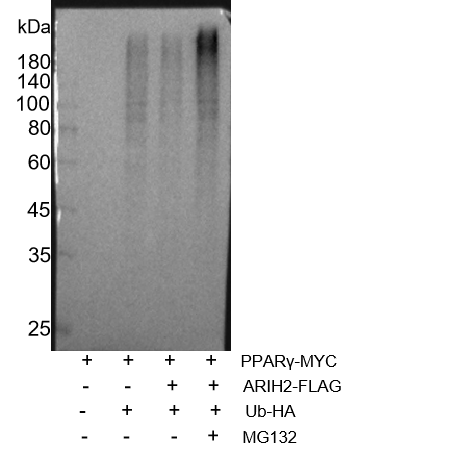

Supplement: Supplementary file 10 — Source data Fig. 7 [file 44318_2025_460_MOESM10_ESM.zip › Figure7/7O/western input HA.tif]

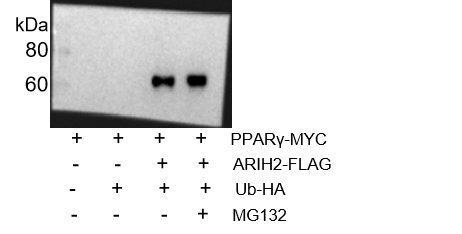

Supplement: Supplementary file 10 — Source data Fig. 7 [file 44318_2025_460_MOESM10_ESM.zip › Figure7/7O/western input FLAG.tif]

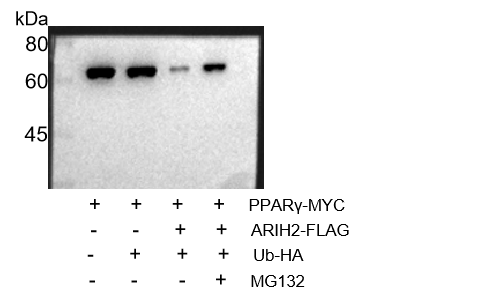

Supplement: Supplementary file 10 — Source data Fig. 7 [file 44318_2025_460_MOESM10_ESM.zip › Figure7/7O/western input MYC.tif]

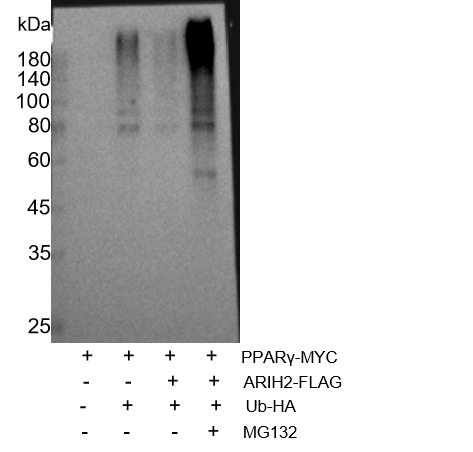

Supplement: Supplementary file 10 — Source data Fig. 7 [file 44318_2025_460_MOESM10_ESM.zip › Figure7/7O/western IP HA.tif]

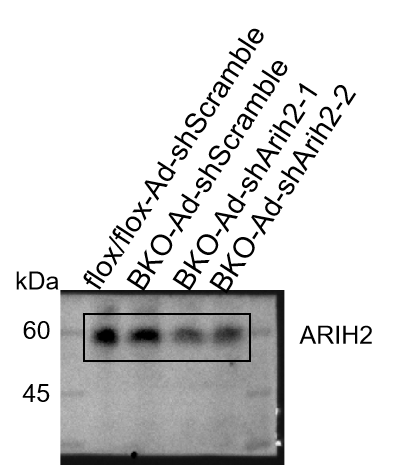

Supplement: Supplementary file 11 — Source data Fig. 8 [file 44318_2025_460_MOESM11_ESM.zip › Figure8/8G/western ARIH2.tif]

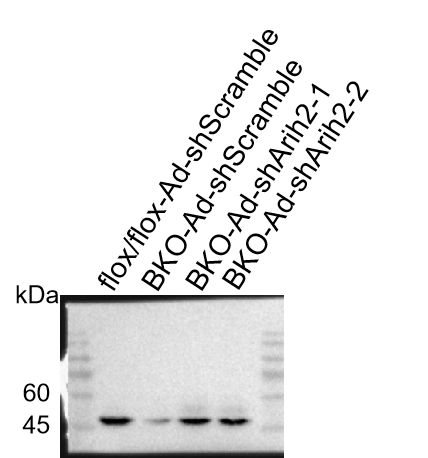

Supplement: Supplementary file 11 — Source data Fig. 8 [file 44318_2025_460_MOESM11_ESM.zip › Figure8/8G/western PPAR╬│.tif]

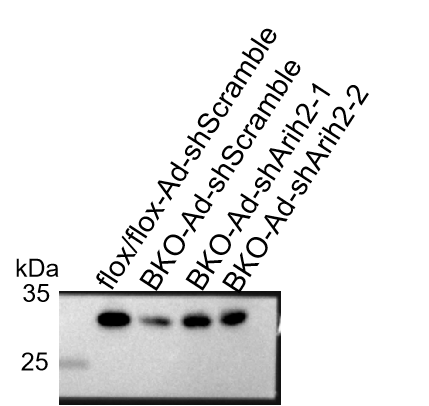

Supplement: Supplementary file 11 — Source data Fig. 8 [file 44318_2025_460_MOESM11_ESM.zip › Figure8/8G/western UCP1.tif]

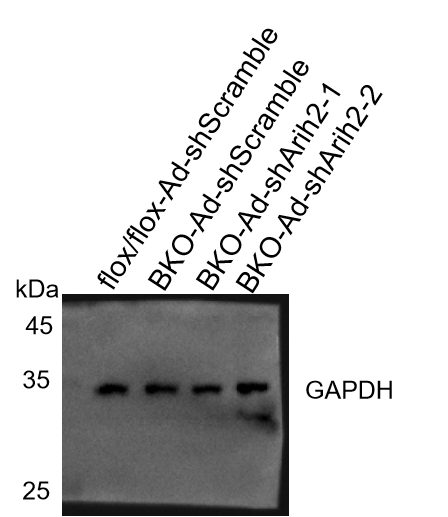

Supplement: Supplementary file 11 — Source data Fig. 8 [file 44318_2025_460_MOESM11_ESM.zip › Figure8/8G/western GAPDH.tif]

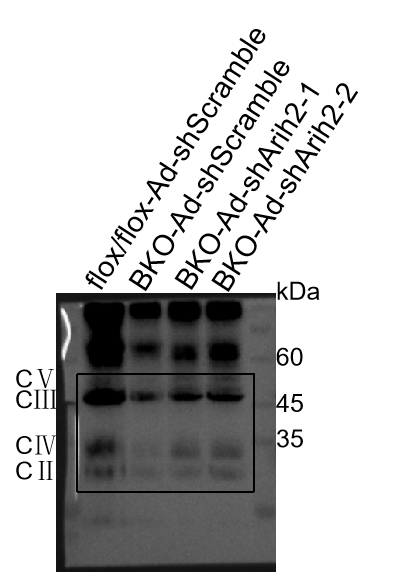

Supplement: Supplementary file 11 — Source data Fig. 8 [file 44318_2025_460_MOESM11_ESM.zip › Figure8/8G/western OXPHOS.tif]

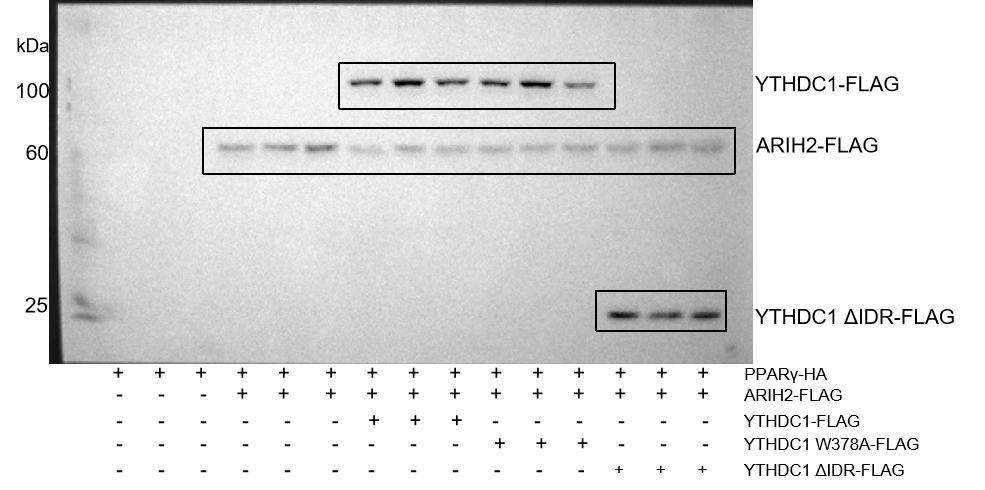

Supplement: Supplementary file 11 — Source data Fig. 8 [file 44318_2025_460_MOESM11_ESM.zip › Figure8/8A/western FLAG.tif]

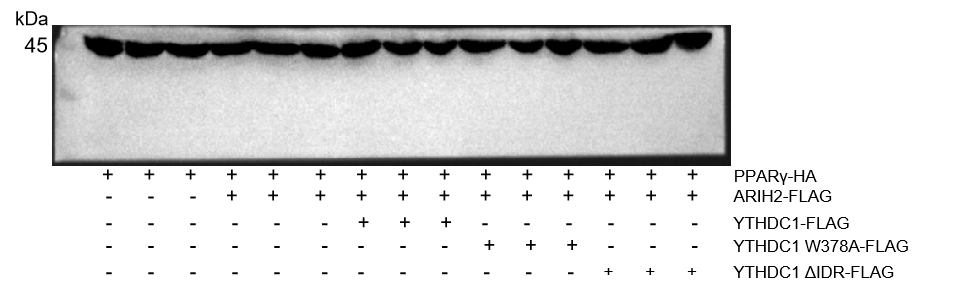

Supplement: Supplementary file 11 — Source data Fig. 8 [file 44318_2025_460_MOESM11_ESM.zip › Figure8/8A/western actin.tif]

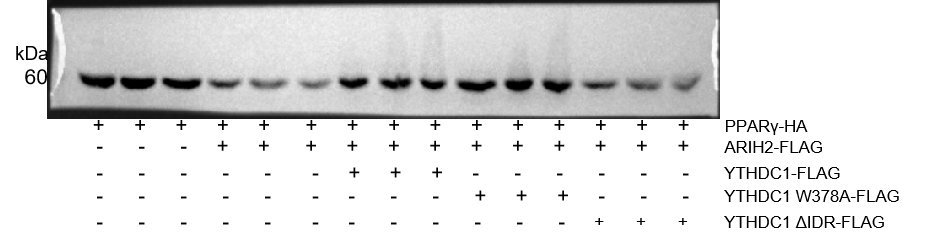

Supplement: Supplementary file 11 — Source data Fig. 8 [file 44318_2025_460_MOESM11_ESM.zip › Figure8/8A/western HA.tif]

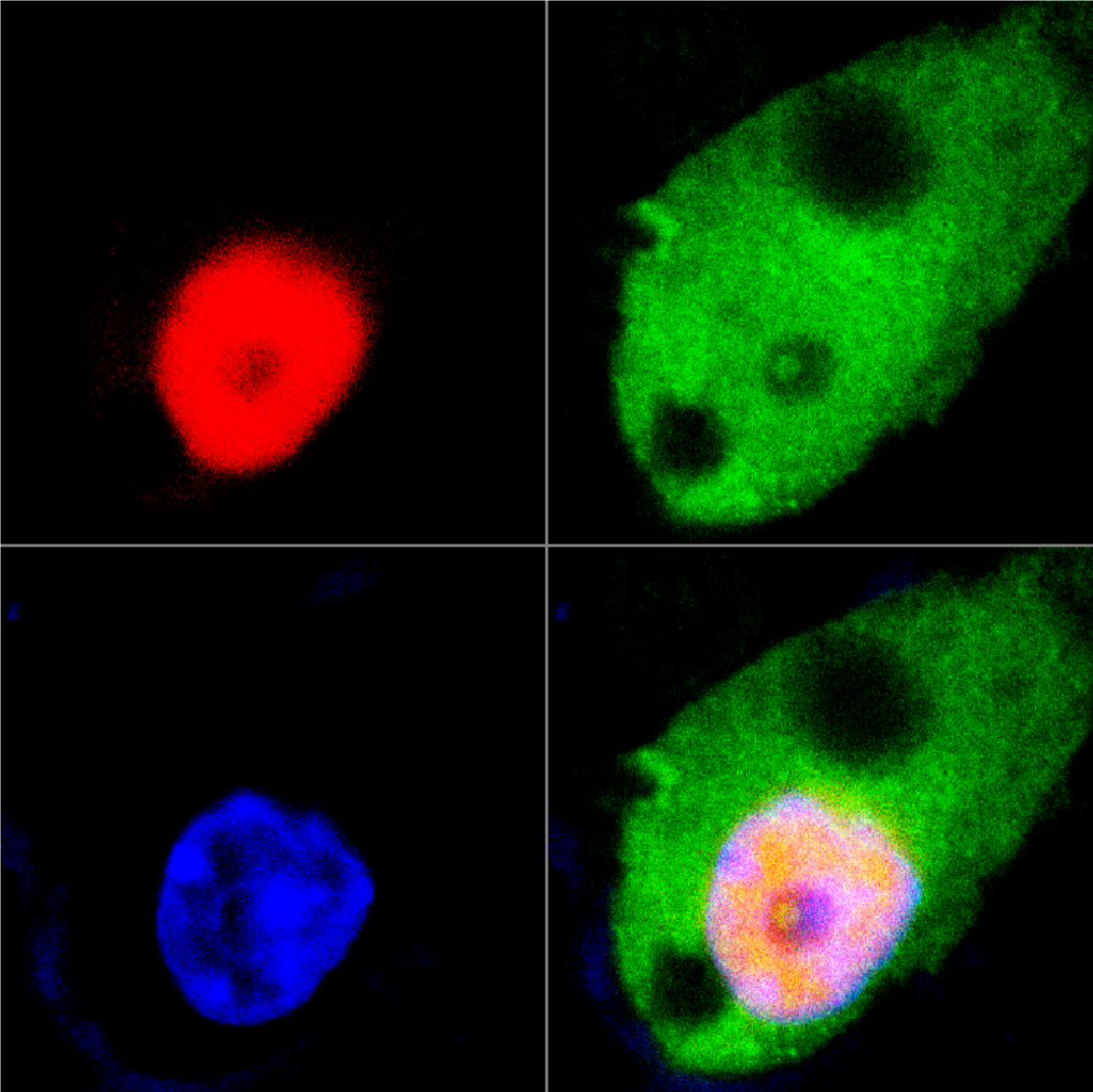

Supplement: Supplementary file 11 — Source data Fig. 8 [file 44318_2025_460_MOESM11_ESM.zip › Figure8/8D/confocal Ad-PPAR╬│-HA+Ad-YTHDC1 ╬öIDR-GFP.tif]

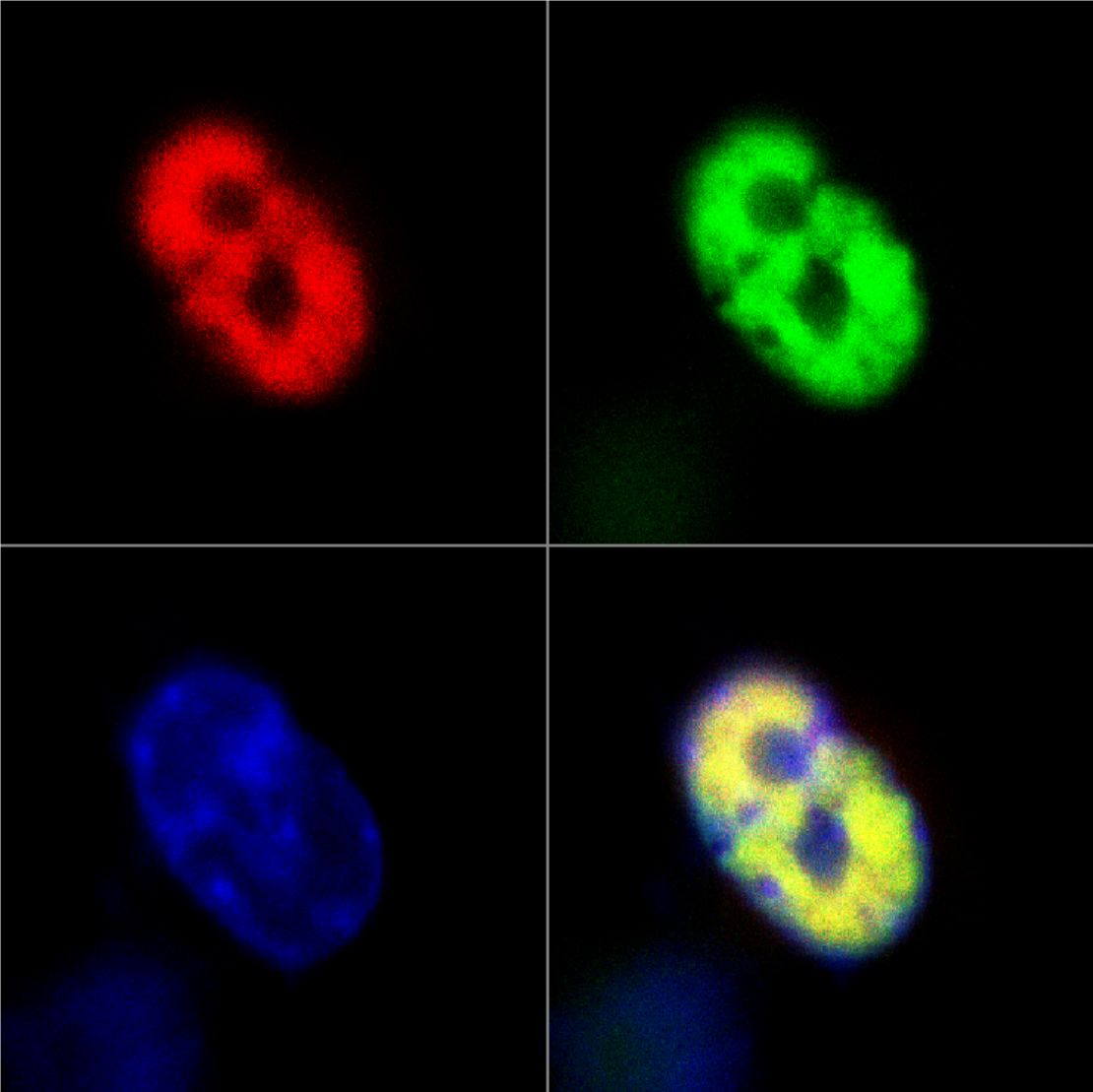

Supplement: Supplementary file 11 — Source data Fig. 8 [file 44318_2025_460_MOESM11_ESM.zip › Figure8/8D/confocal Ad-PPAR╬│-HA+Ad-YTHDC1 W378A-GFP.tif]

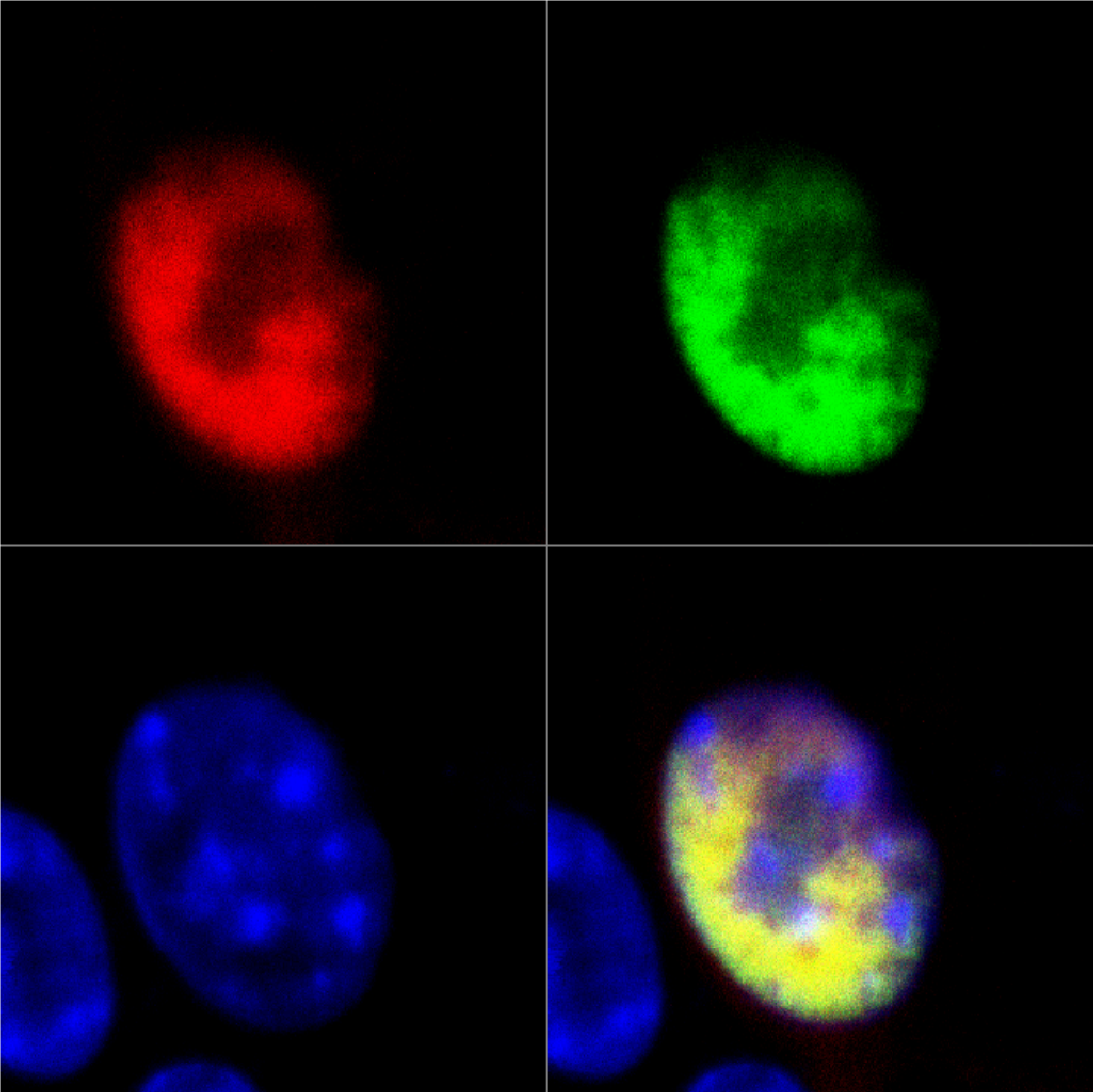

Supplement: Supplementary file 11 — Source data Fig. 8 [file 44318_2025_460_MOESM11_ESM.zip › Figure8/8D/confocal Ad-PPAR╬│-HA+Ad-YTHDC1-GFP.tif]

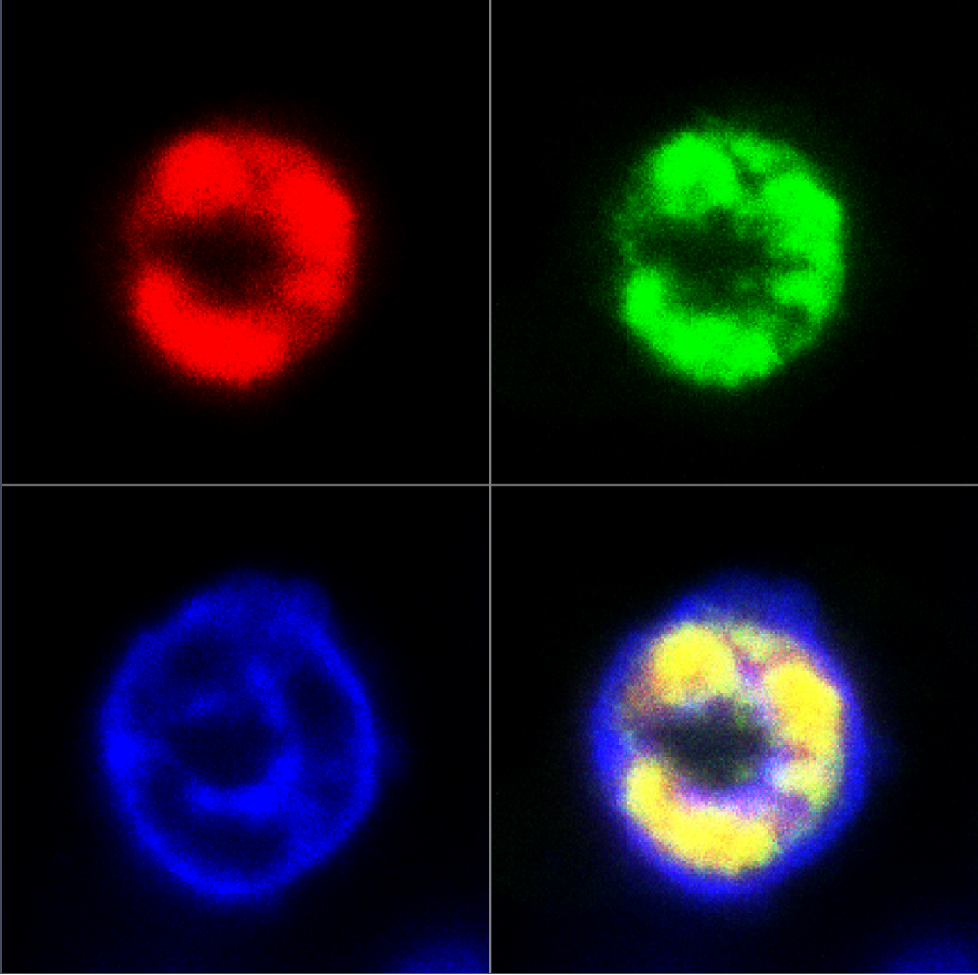

Supplement: Supplementary file 11 — Source data Fig. 8 [file 44318_2025_460_MOESM11_ESM.zip › Figure8/8C/confocal PPAR╬│-HA+YTHDC1 W378A-GFP.tif]

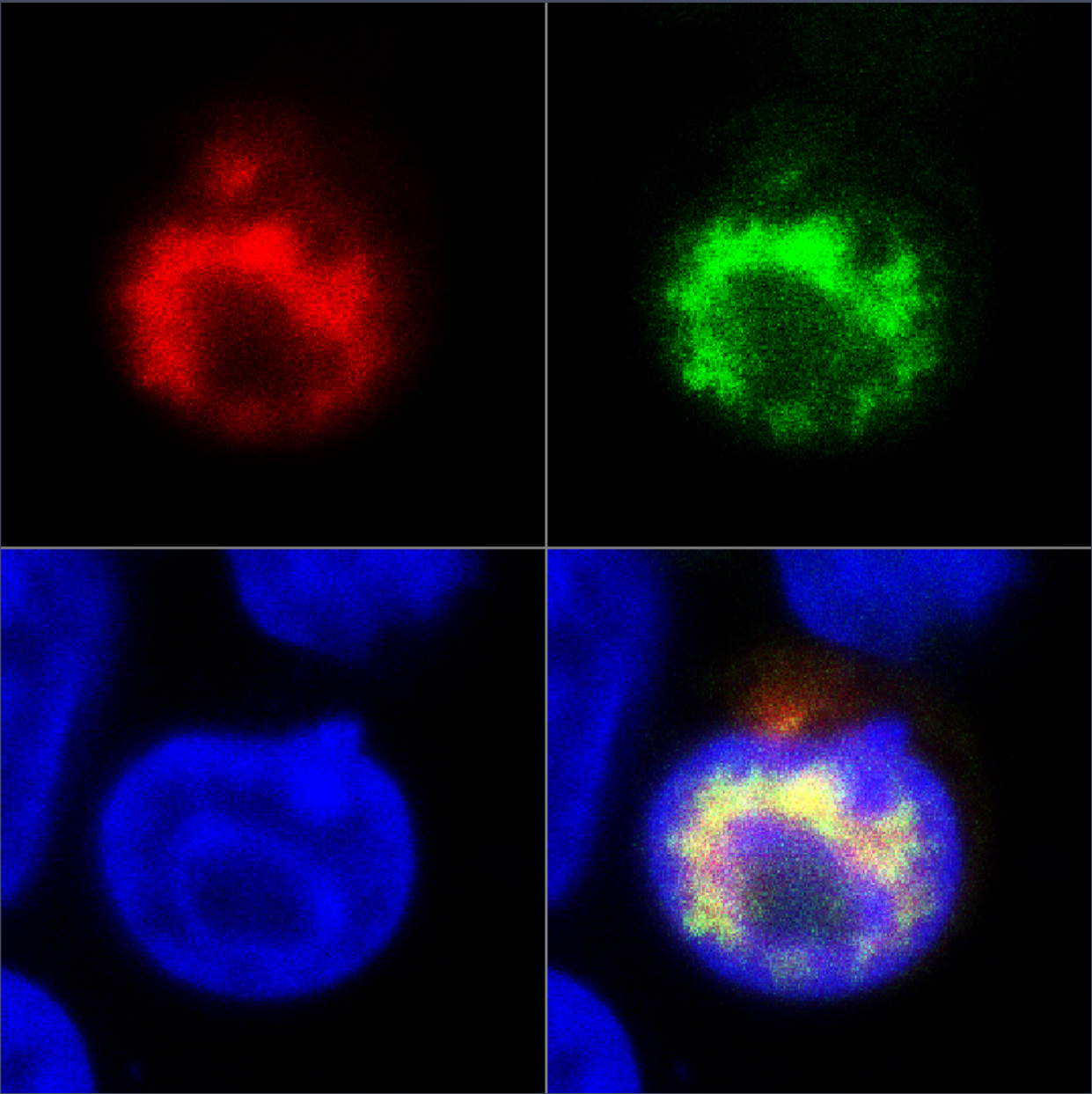

Supplement: Supplementary file 11 — Source data Fig. 8 [file 44318_2025_460_MOESM11_ESM.zip › Figure8/8C/confocal PPAR╬│-HA+YTHDC1-GFP.tif]

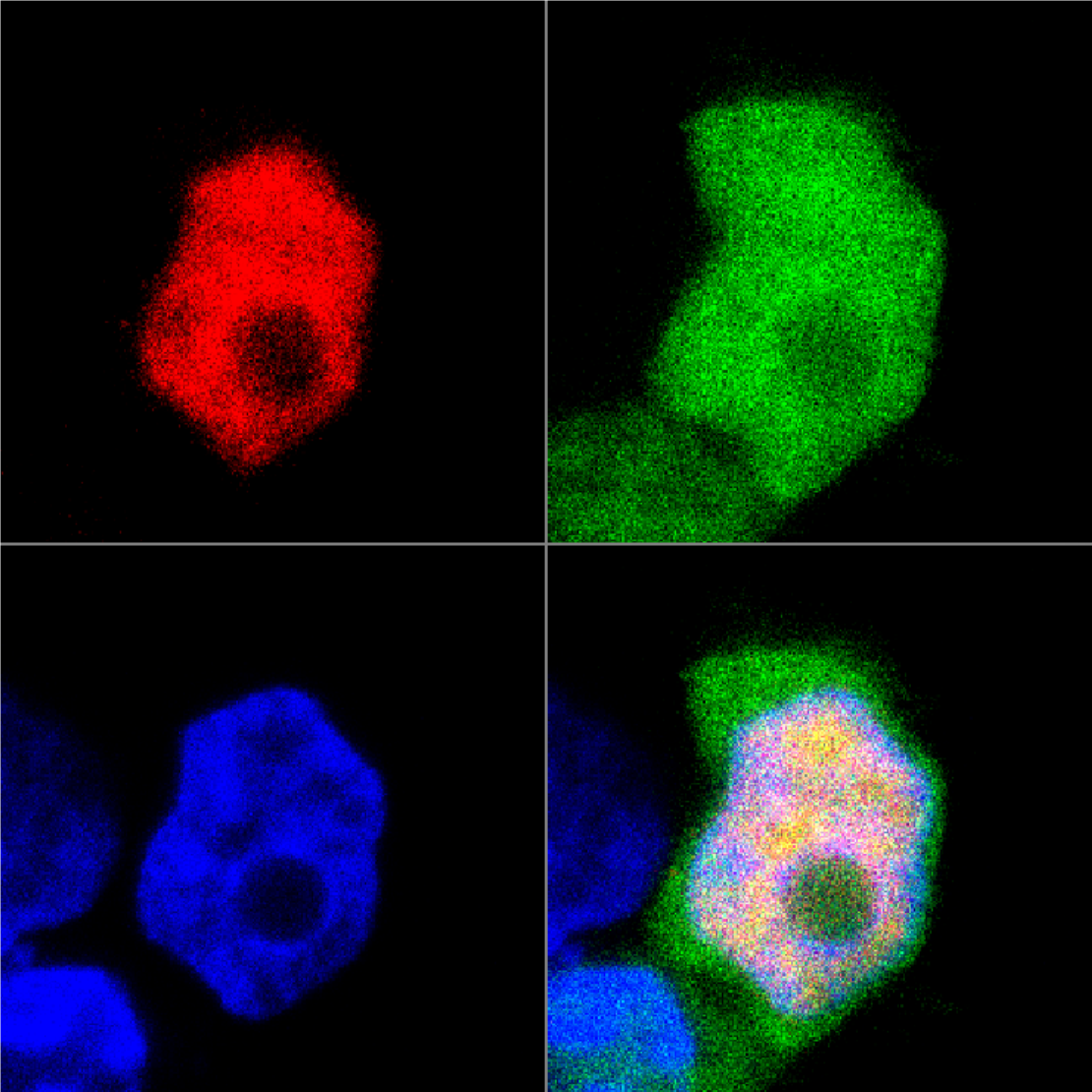

Supplement: Supplementary file 11 — Source data Fig. 8 [file 44318_2025_460_MOESM11_ESM.zip › Figure8/8C/confocal PPAR╬│-HA+YTHDC1╬öIDR-GFP.tif]

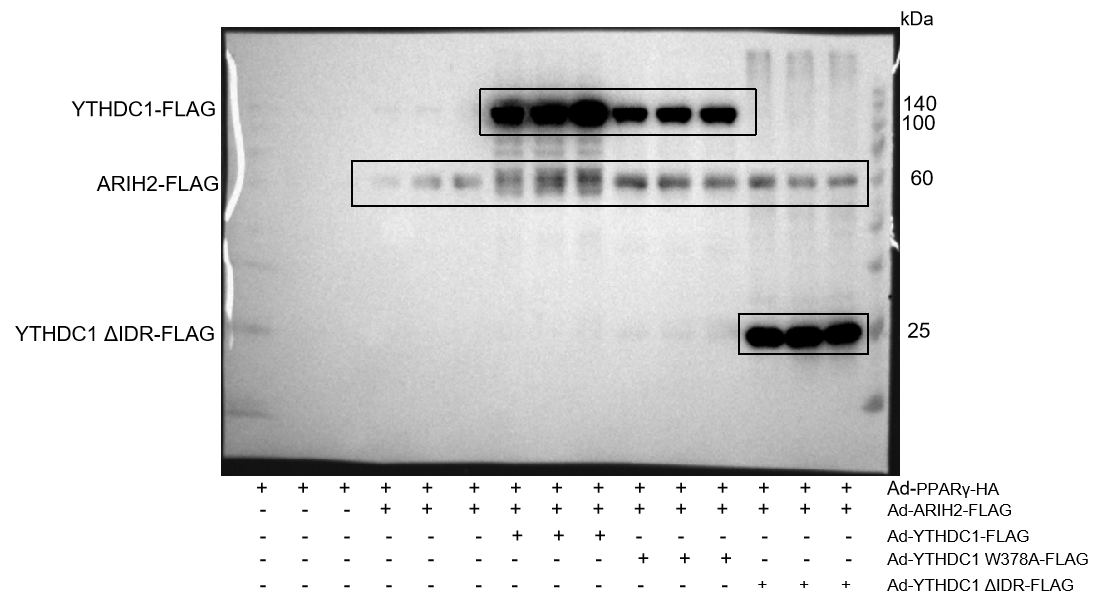

Supplement: Supplementary file 11 — Source data Fig. 8 [file 44318_2025_460_MOESM11_ESM.zip › Figure8/8B/western FLAG.tif]

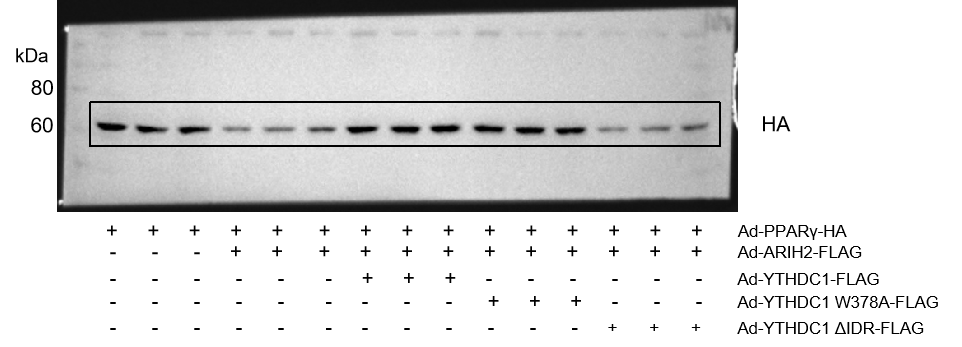

Supplement: Supplementary file 11 — Source data Fig. 8 [file 44318_2025_460_MOESM11_ESM.zip › Figure8/8B/western HA.tif]

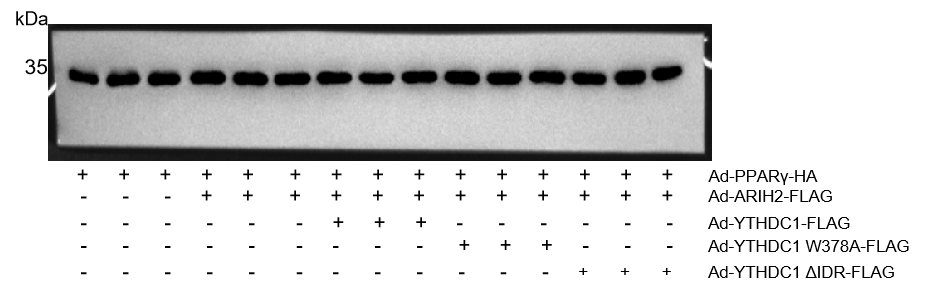

Supplement: Supplementary file 11 — Source data Fig. 8 [file 44318_2025_460_MOESM11_ESM.zip › Figure8/8B/western GAPDH.tif]

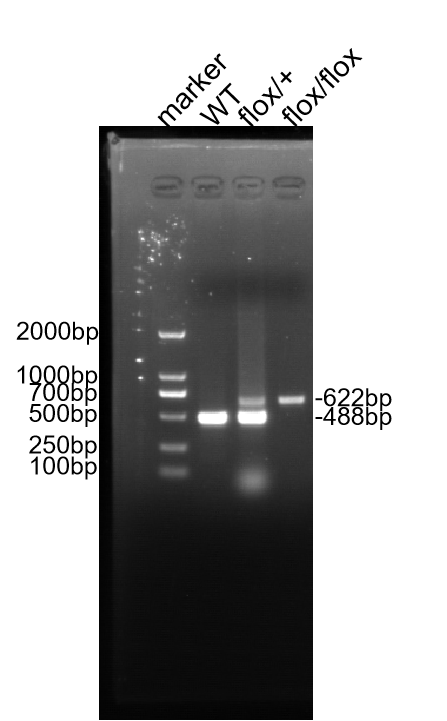

Supplement: Supplementary file 12 — Appendix Figure Source Data [file 44318_2025_460_MOESM12_ESM.zip › Figure S2/S2B/σƒ║σ¢áΘë┤σ«Ü.tif]

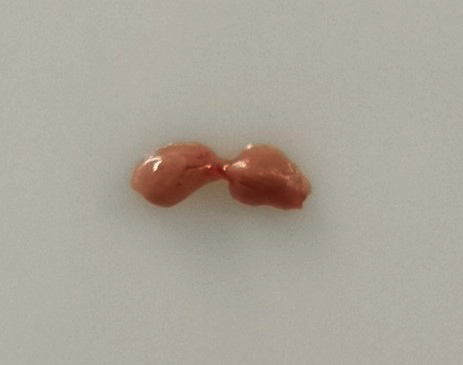

Supplement: Supplementary file 12 — Appendix Figure Source Data [file 44318_2025_460_MOESM12_ESM.zip › Figure S2/S2D/Ucp1-iCre iBAT.tif]

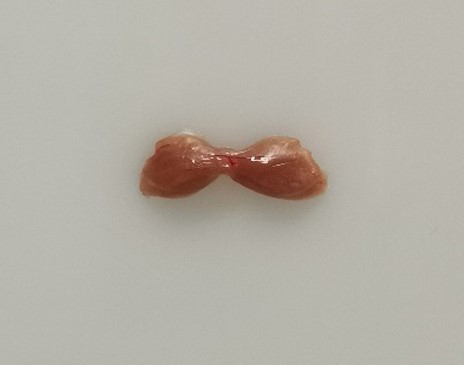

Supplement: Supplementary file 12 — Appendix Figure Source Data [file 44318_2025_460_MOESM12_ESM.zip › Figure S2/S2D/floxflox iBAT.tif]

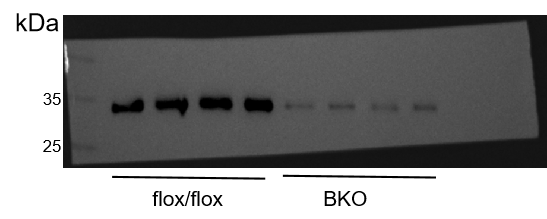

Supplement: Supplementary file 12 — Appendix Figure Source Data [file 44318_2025_460_MOESM12_ESM.zip › Figure S3/S3J/western UCP1.tif]

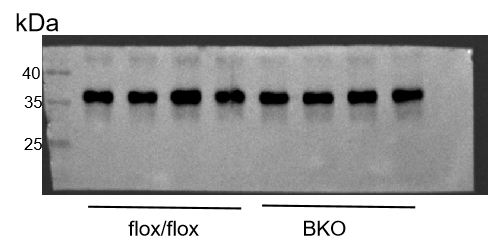

Supplement: Supplementary file 12 — Appendix Figure Source Data [file 44318_2025_460_MOESM12_ESM.zip › Figure S3/S3J/western GAPDH.tif]

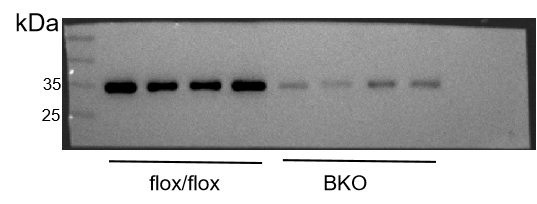

Supplement: Supplementary file 12 — Appendix Figure Source Data [file 44318_2025_460_MOESM12_ESM.zip › Figure S3/S3C/western UCP1.tif]

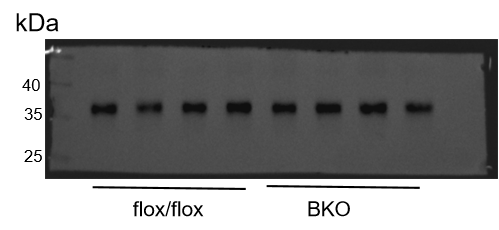

Supplement: Supplementary file 12 — Appendix Figure Source Data [file 44318_2025_460_MOESM12_ESM.zip › Figure S3/S3C/western GAPDH.tif]

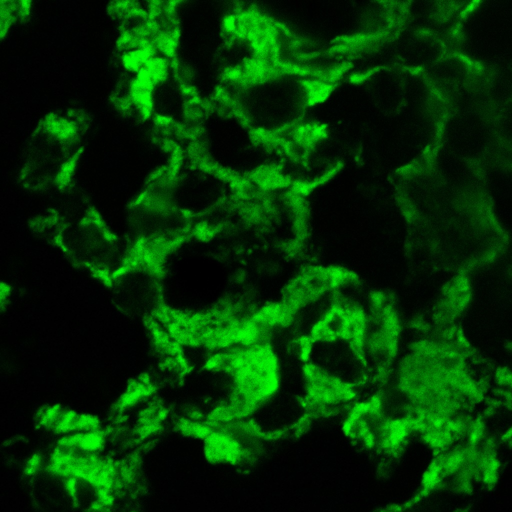

Supplement: Supplementary file 12 — Appendix Figure Source Data [file 44318_2025_460_MOESM12_ESM.zip › Figure S3/S3D/ff confocal UCP1.tif]

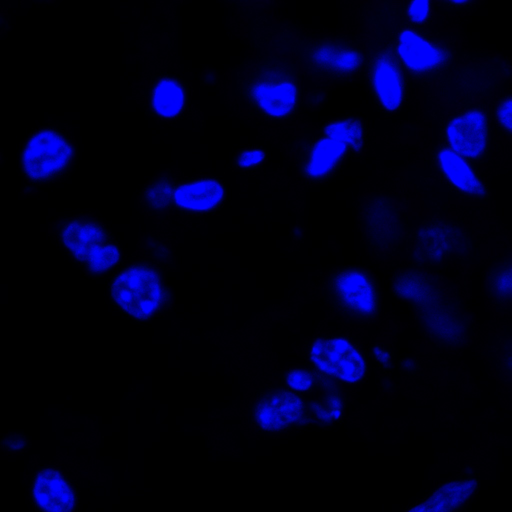

Supplement: Supplementary file 12 — Appendix Figure Source Data [file 44318_2025_460_MOESM12_ESM.zip › Figure S3/S3D/ff confocal DAPI.tif]

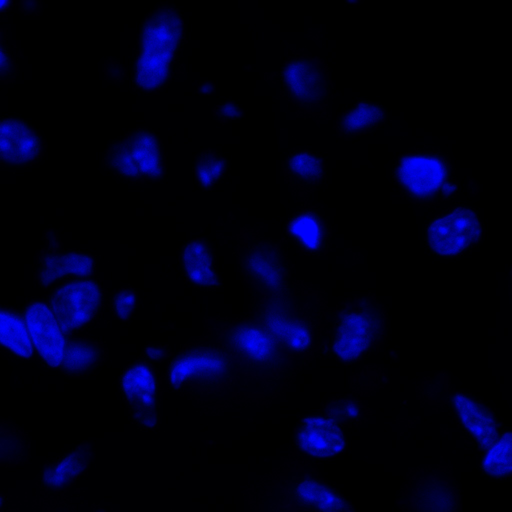

Supplement: Supplementary file 12 — Appendix Figure Source Data [file 44318_2025_460_MOESM12_ESM.zip › Figure S3/S3D/BKO confocal DAPI.tif]

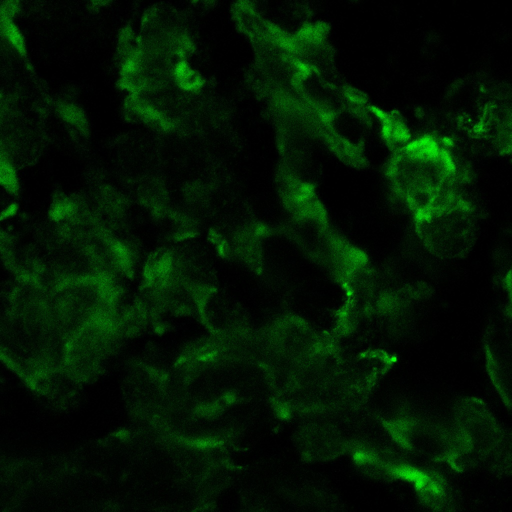

Supplement: Supplementary file 12 — Appendix Figure Source Data [file 44318_2025_460_MOESM12_ESM.zip › Figure S3/S3D/BKO confocal UCP1.tif]

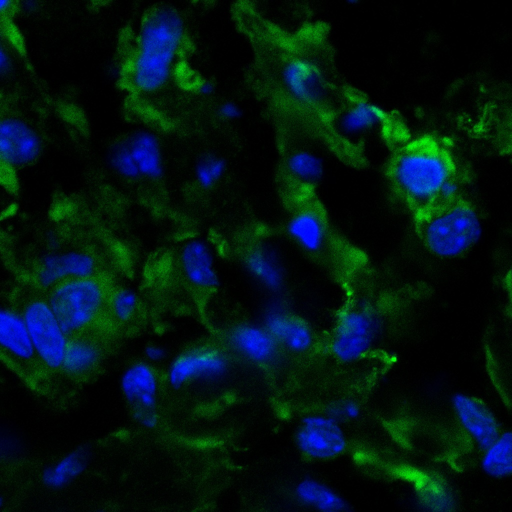

Supplement: Supplementary file 12 — Appendix Figure Source Data [file 44318_2025_460_MOESM12_ESM.zip › Figure S3/S3D/BKO confocal Merge.tif]

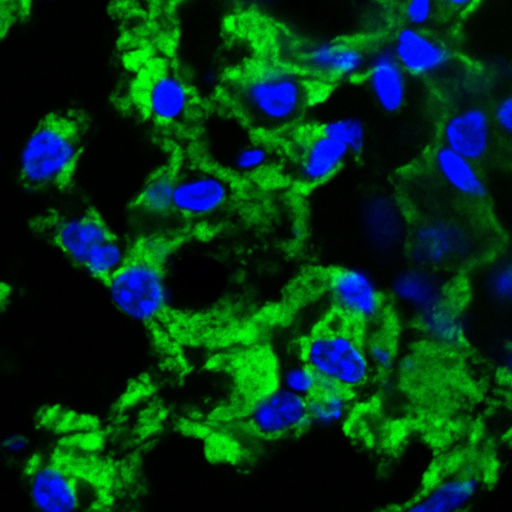

Supplement: Supplementary file 12 — Appendix Figure Source Data [file 44318_2025_460_MOESM12_ESM.zip › Figure S3/S3D/ff confocal Merge.tif]

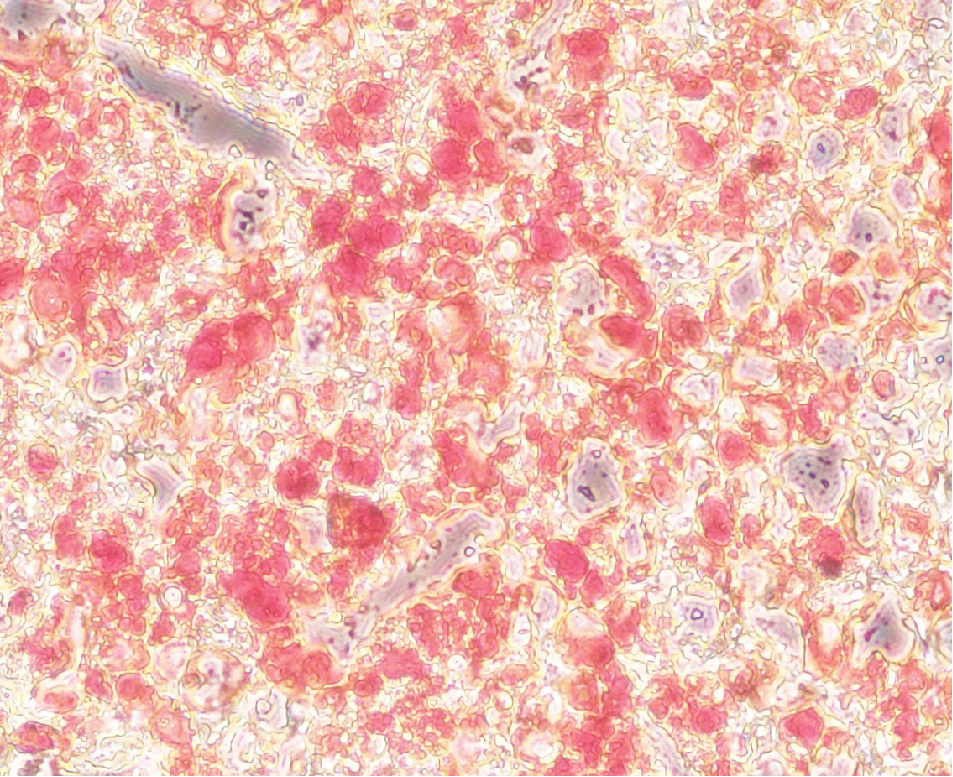

Supplement: Supplementary file 12 — Appendix Figure Source Data [file 44318_2025_460_MOESM12_ESM.zip › Figure S3/S3B/BKO iBAT oil red.tif]

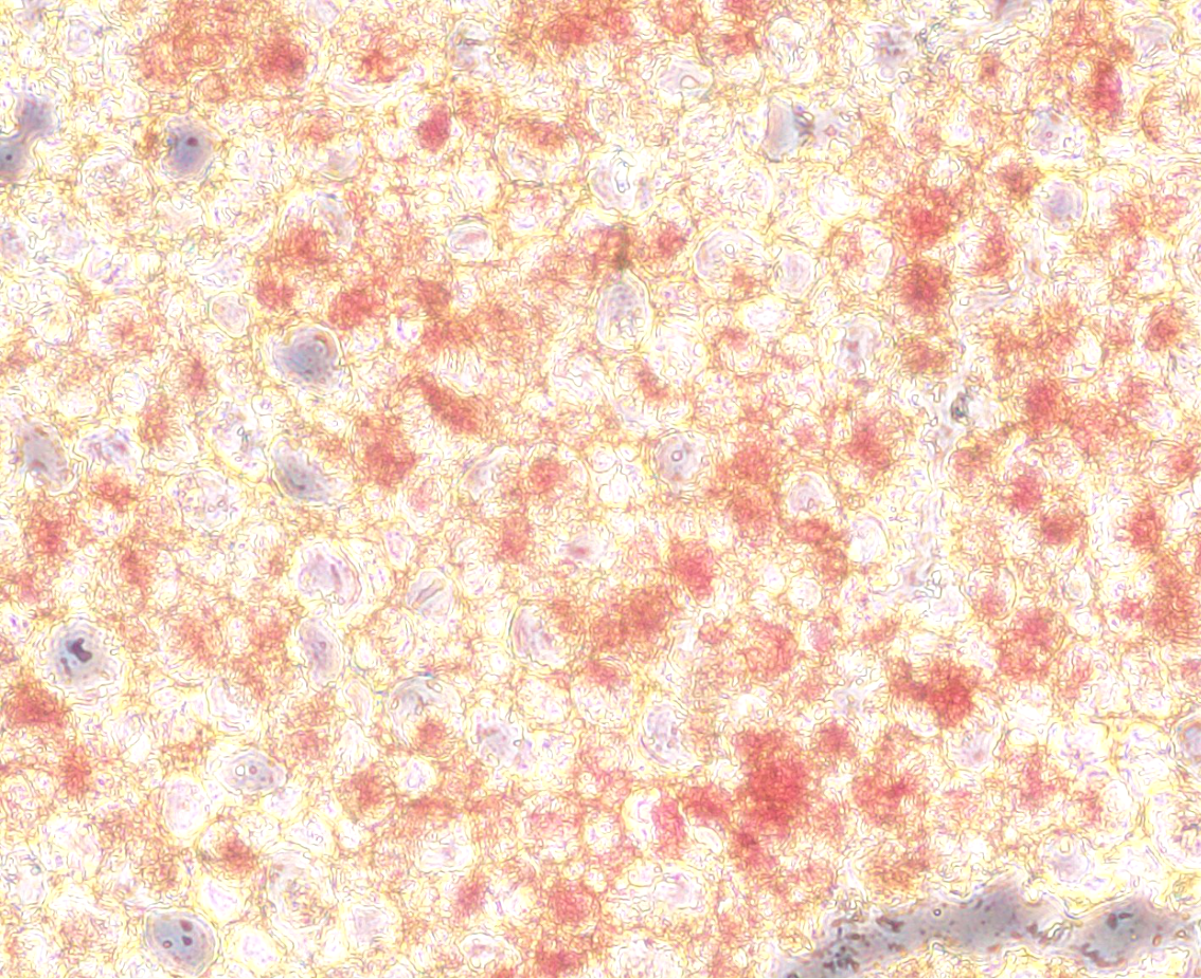

Supplement: Supplementary file 12 — Appendix Figure Source Data [file 44318_2025_460_MOESM12_ESM.zip › Figure S3/S3B/ff iBAT oil red.tif]

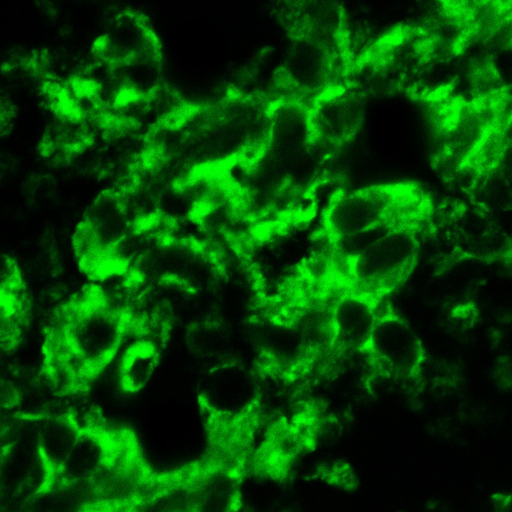

Supplement: Supplementary file 12 — Appendix Figure Source Data [file 44318_2025_460_MOESM12_ESM.zip › Figure S3/S3K/ff confocal UCP1.tif]

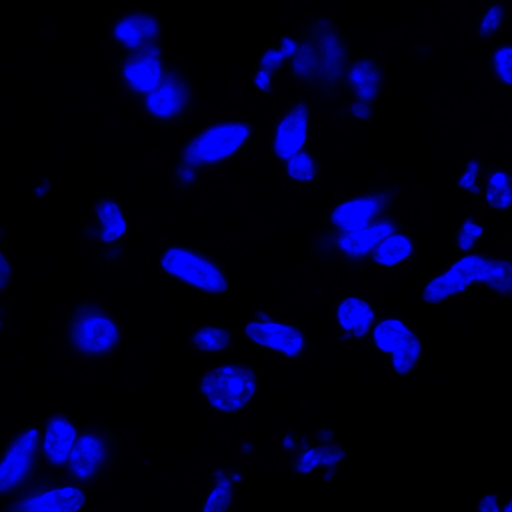

Supplement: Supplementary file 12 — Appendix Figure Source Data [file 44318_2025_460_MOESM12_ESM.zip › Figure S3/S3K/ff confocal DAPI.tif]

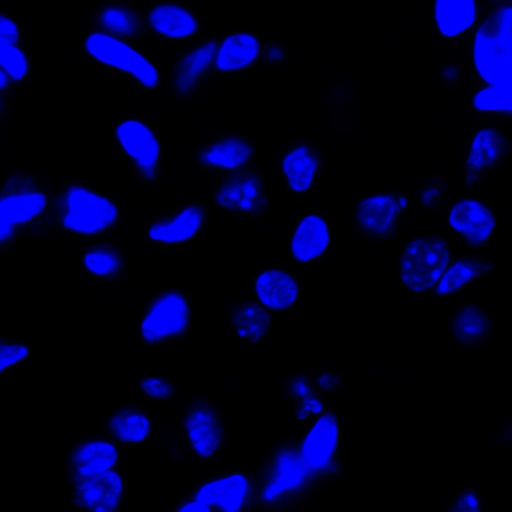

Supplement: Supplementary file 12 — Appendix Figure Source Data [file 44318_2025_460_MOESM12_ESM.zip › Figure S3/S3K/BKO confocal DAPI.tif]

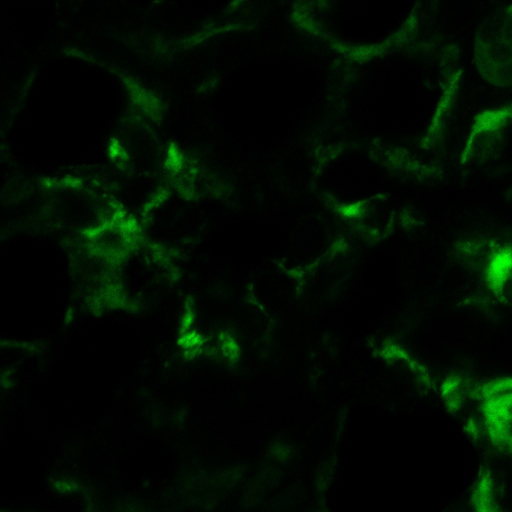

Supplement: Supplementary file 12 — Appendix Figure Source Data [file 44318_2025_460_MOESM12_ESM.zip › Figure S3/S3K/BKO confocal UCP1.tif]

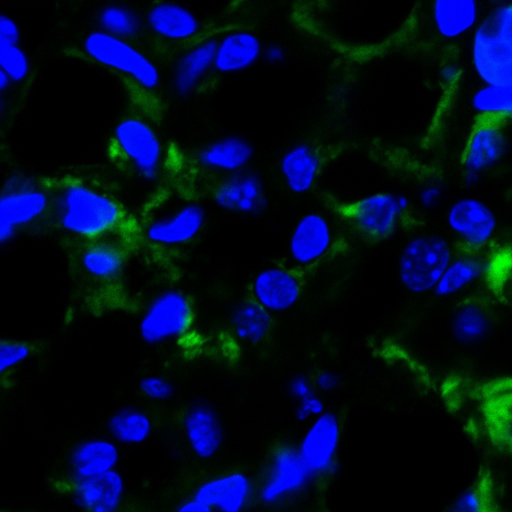

Supplement: Supplementary file 12 — Appendix Figure Source Data [file 44318_2025_460_MOESM12_ESM.zip › Figure S3/S3K/BKO confocal Merge.tif]

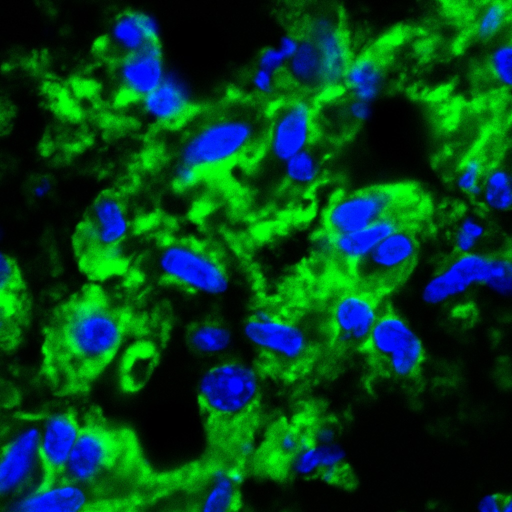

Supplement: Supplementary file 12 — Appendix Figure Source Data [file 44318_2025_460_MOESM12_ESM.zip › Figure S3/S3K/ff confocal Merge.tif]

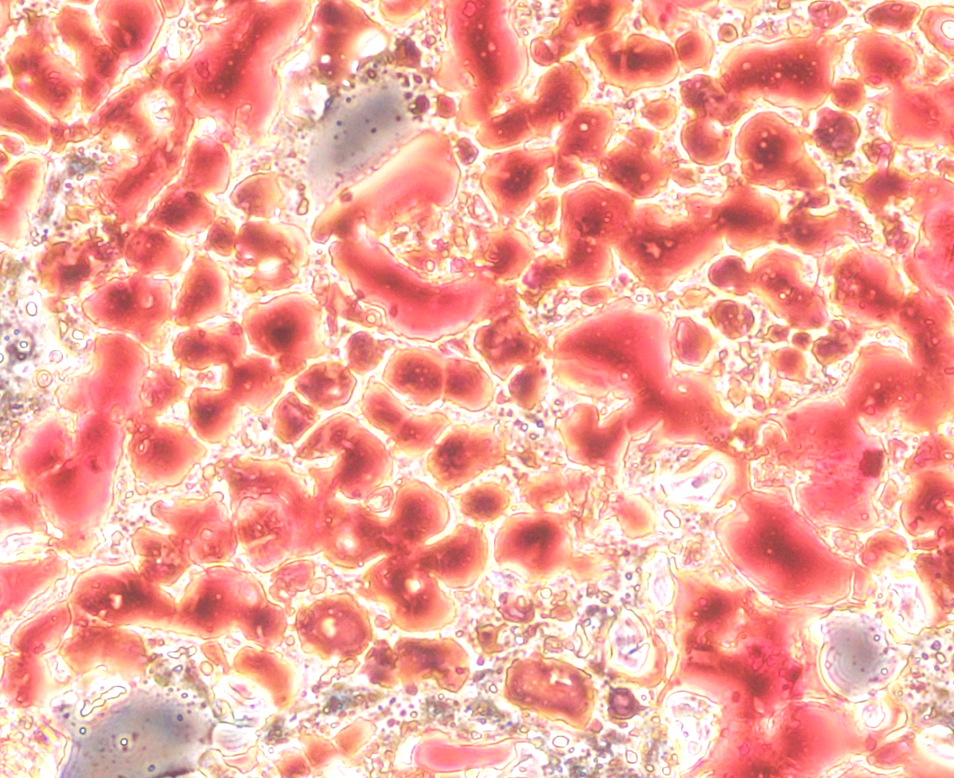

Supplement: Supplementary file 12 — Appendix Figure Source Data [file 44318_2025_460_MOESM12_ESM.zip › Figure S3/S3I/BKO oil red.tif]

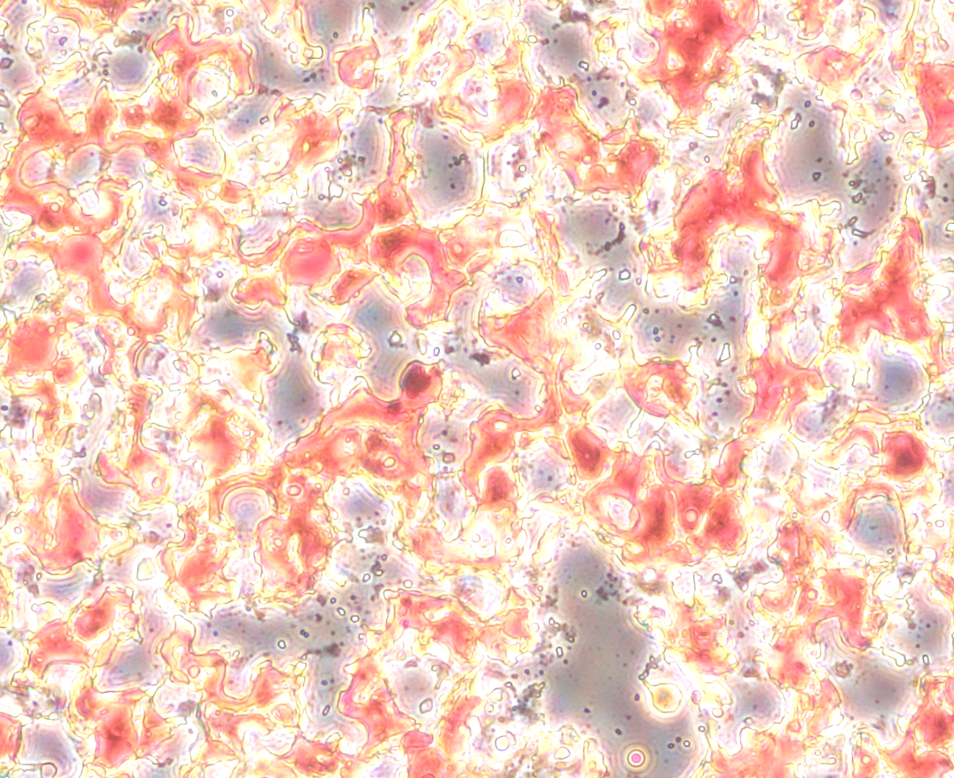

Supplement: Supplementary file 12 — Appendix Figure Source Data [file 44318_2025_460_MOESM12_ESM.zip › Figure S3/S3I/ff oil red.tif]

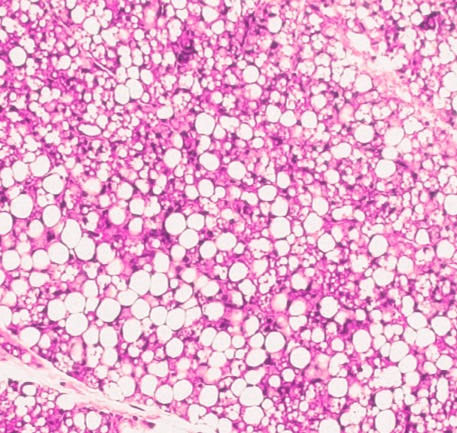

Supplement: Supplementary file 12 — Appendix Figure Source Data [file 44318_2025_460_MOESM12_ESM.zip › Figure S3/S3A/ff iBAT HE.tif]

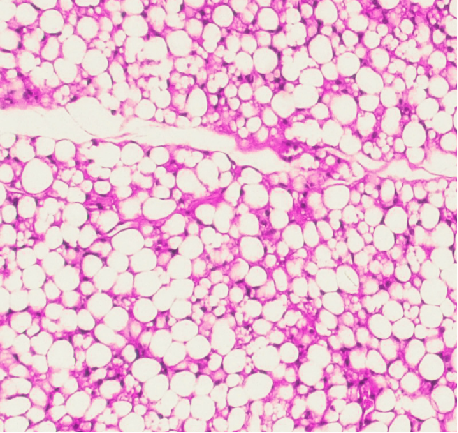

Supplement: Supplementary file 12 — Appendix Figure Source Data [file 44318_2025_460_MOESM12_ESM.zip › Figure S3/S3A/BKO iBAT HE.tif]

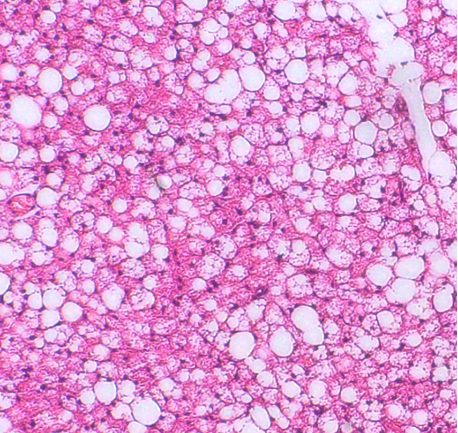

Supplement: Supplementary file 12 — Appendix Figure Source Data [file 44318_2025_460_MOESM12_ESM.zip › Figure S3/S3H/ff iBAT HE.tif]

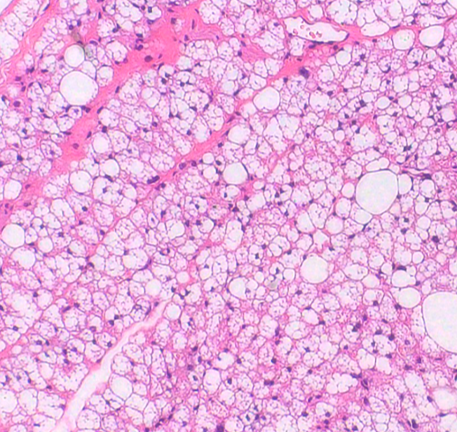

Supplement: Supplementary file 12 — Appendix Figure Source Data [file 44318_2025_460_MOESM12_ESM.zip › Figure S3/S3H/BKO iBAT HE.tif]

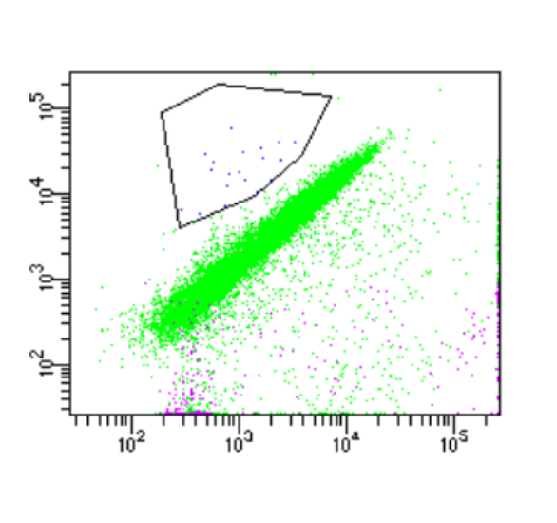

Supplement: Supplementary file 12 — Appendix Figure Source Data [file 44318_2025_460_MOESM12_ESM.zip › Figure S4/S4C/FKO-2.tif]

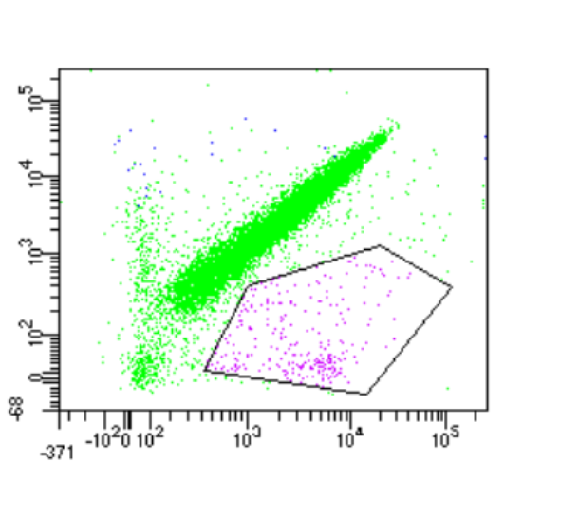

Supplement: Supplementary file 12 — Appendix Figure Source Data [file 44318_2025_460_MOESM12_ESM.zip › Figure S4/S4C/FKO-3.tif]

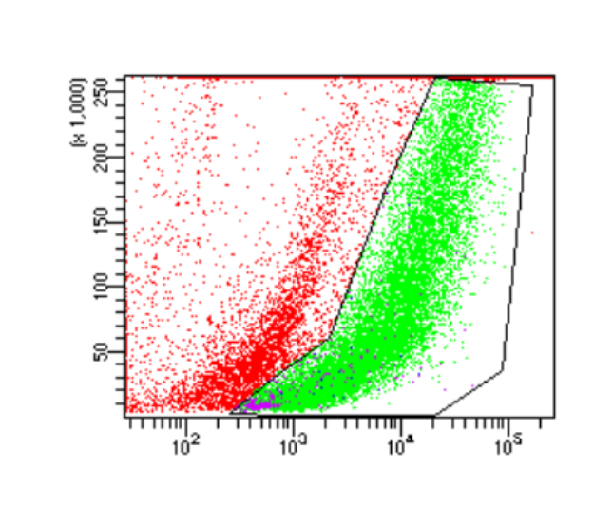

Supplement: Supplementary file 12 — Appendix Figure Source Data [file 44318_2025_460_MOESM12_ESM.zip › Figure S4/S4C/FKO-1.tif]

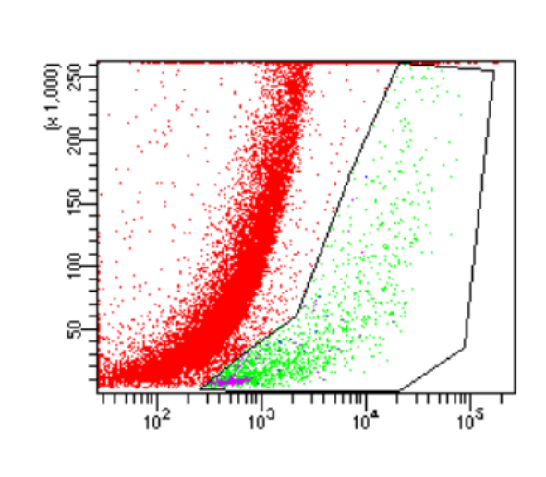

Supplement: Supplementary file 12 — Appendix Figure Source Data [file 44318_2025_460_MOESM12_ESM.zip › Figure S4/S4C/ff-1.tif]

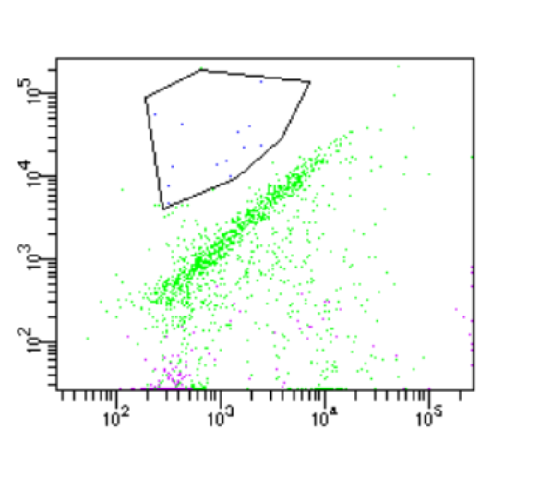

Supplement: Supplementary file 12 — Appendix Figure Source Data [file 44318_2025_460_MOESM12_ESM.zip › Figure S4/S4C/ff-2.tif]

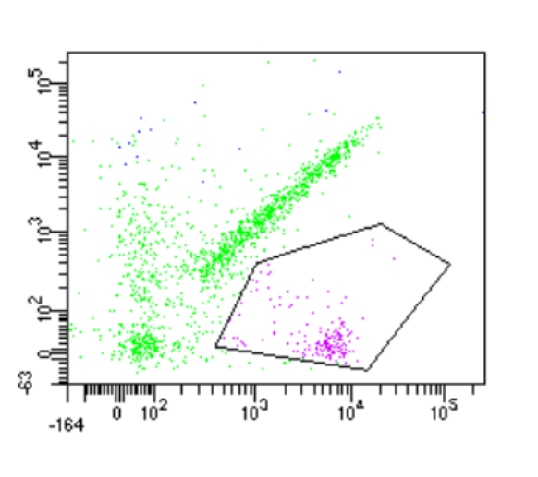

Supplement: Supplementary file 12 — Appendix Figure Source Data [file 44318_2025_460_MOESM12_ESM.zip › Figure S4/S4C/ff-3.tif]

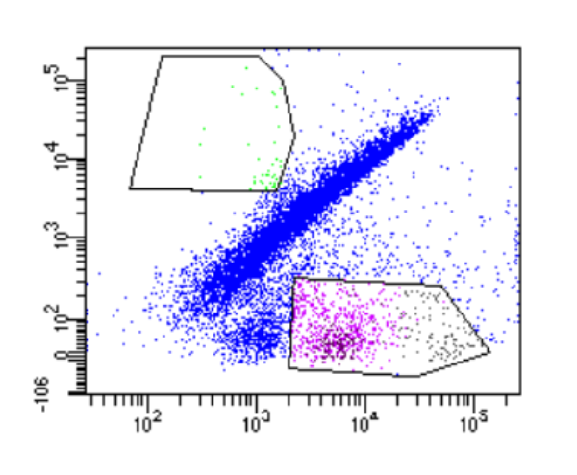

Supplement: Supplementary file 12 — Appendix Figure Source Data [file 44318_2025_460_MOESM12_ESM.zip › Figure S4/S4B/FKO-2.tif]

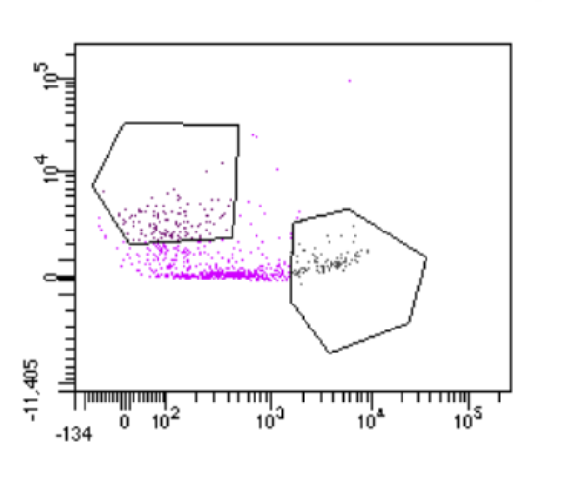

Supplement: Supplementary file 12 — Appendix Figure Source Data [file 44318_2025_460_MOESM12_ESM.zip › Figure S4/S4B/FKO-3.tif]

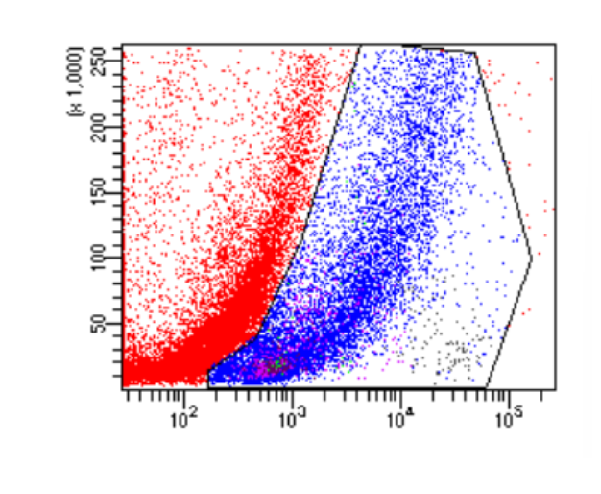

Supplement: Supplementary file 12 — Appendix Figure Source Data [file 44318_2025_460_MOESM12_ESM.zip › Figure S4/S4B/FKO-1.tif]

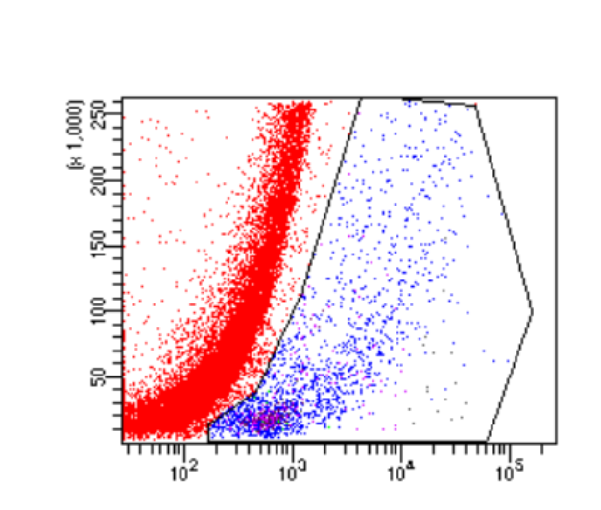

Supplement: Supplementary file 12 — Appendix Figure Source Data [file 44318_2025_460_MOESM12_ESM.zip › Figure S4/S4B/ff-1.tif]

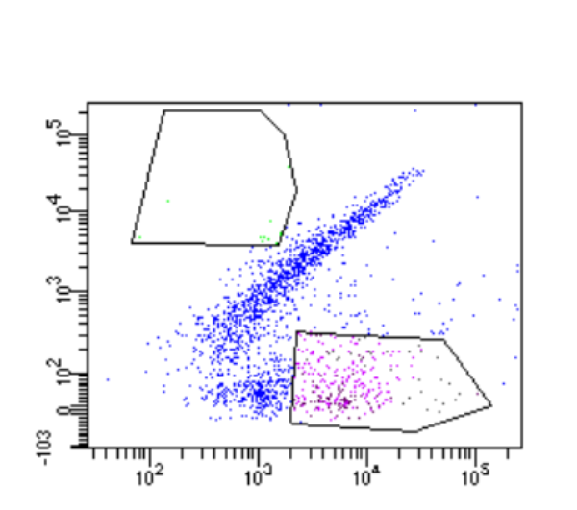

Supplement: Supplementary file 12 — Appendix Figure Source Data [file 44318_2025_460_MOESM12_ESM.zip › Figure S4/S4B/ff-2.tif]

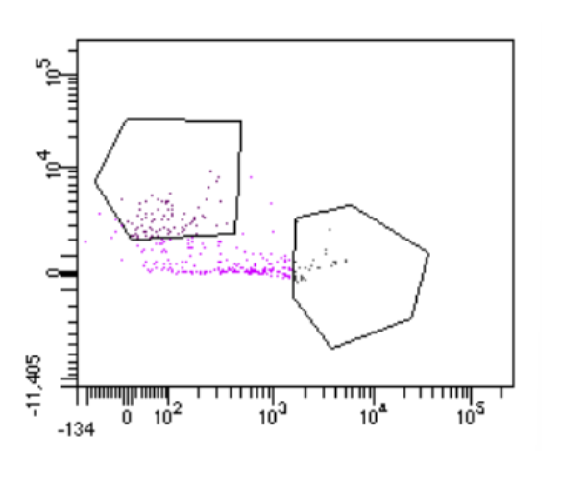

Supplement: Supplementary file 12 — Appendix Figure Source Data [file 44318_2025_460_MOESM12_ESM.zip › Figure S4/S4B/ff-3.tif]

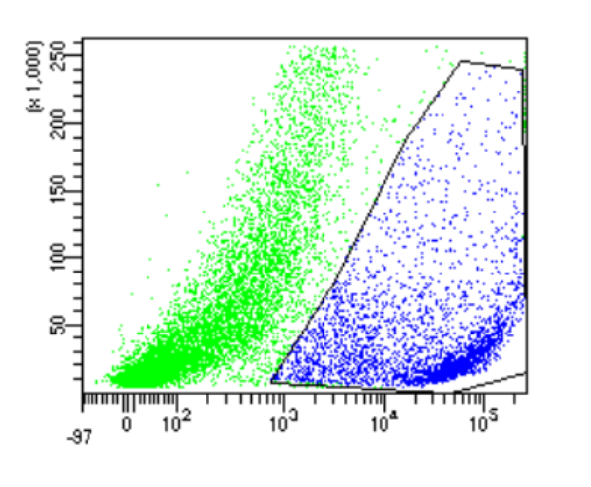

Supplement: Supplementary file 12 — Appendix Figure Source Data [file 44318_2025_460_MOESM12_ESM.zip › Figure S4/S4A/FKO-2.tif]

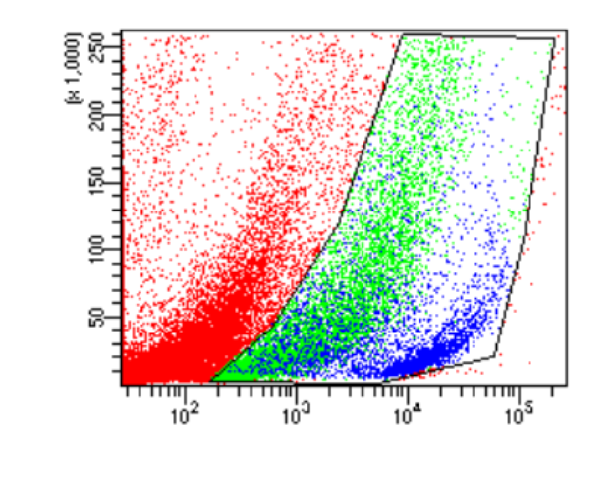

Supplement: Supplementary file 12 — Appendix Figure Source Data [file 44318_2025_460_MOESM12_ESM.zip › Figure S4/S4A/FKO-1.tif]

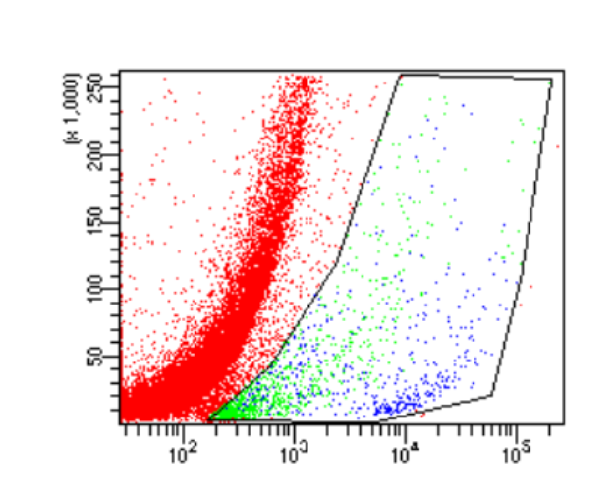

Supplement: Supplementary file 12 — Appendix Figure Source Data [file 44318_2025_460_MOESM12_ESM.zip › Figure S4/S4A/ff-1.tif]

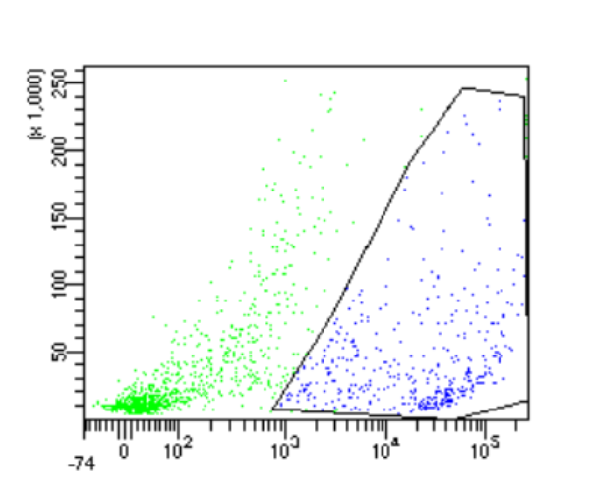

Supplement: Supplementary file 12 — Appendix Figure Source Data [file 44318_2025_460_MOESM12_ESM.zip › Figure S4/S4A/ff-2.tif]

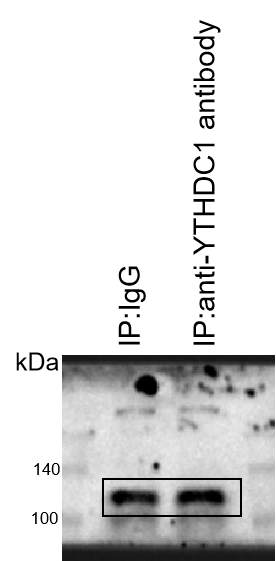

Supplement: Supplementary file 12 — Appendix Figure Source Data [file 44318_2025_460_MOESM12_ESM.zip › Figure S6/S6A/western Input YTHDC1.tif]

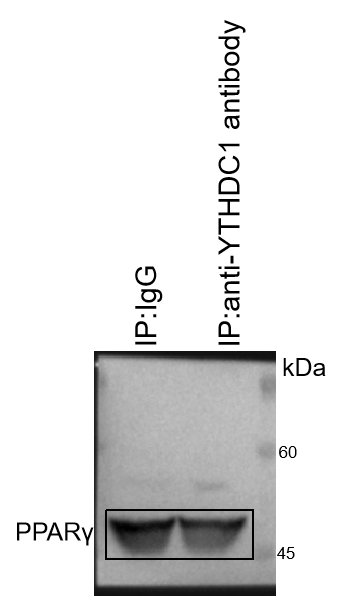

Supplement: Supplementary file 12 — Appendix Figure Source Data [file 44318_2025_460_MOESM12_ESM.zip › Figure S6/S6A/western Input PPAR╬│.tif]

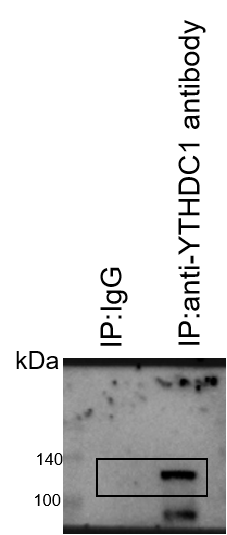

Supplement: Supplementary file 12 — Appendix Figure Source Data [file 44318_2025_460_MOESM12_ESM.zip › Figure S6/S6A/western IB YTHDC1.tif]

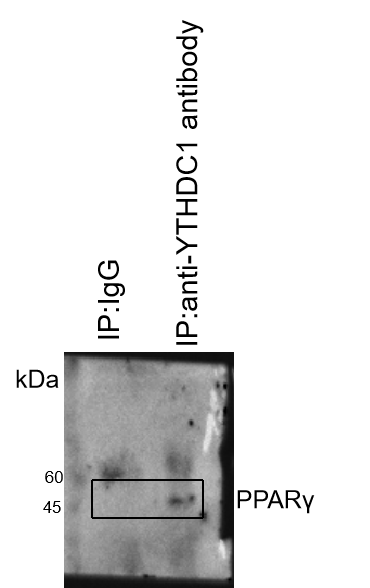

Supplement: Supplementary file 12 — Appendix Figure Source Data [file 44318_2025_460_MOESM12_ESM.zip › Figure S6/S6A/western IB PPAR╬│.tif]

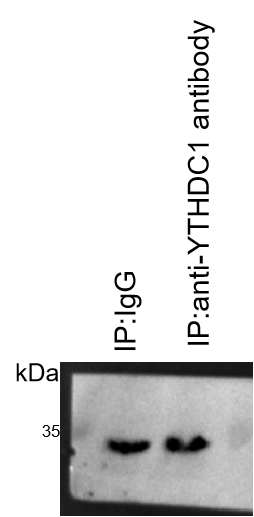

Supplement: Supplementary file 12 — Appendix Figure Source Data [file 44318_2025_460_MOESM12_ESM.zip › Figure S6/S6A/western GAPDH.tif]

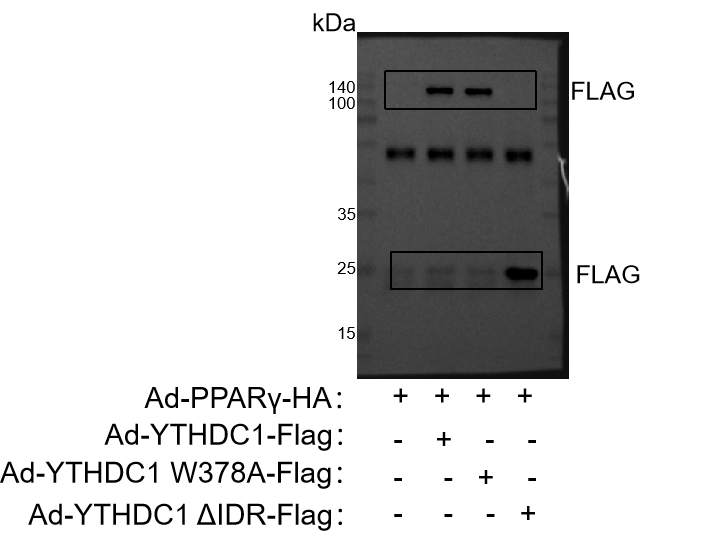

Supplement: Supplementary file 12 — Appendix Figure Source Data [file 44318_2025_460_MOESM12_ESM.zip › Figure S6/S6B/western IP FLAG.tif]

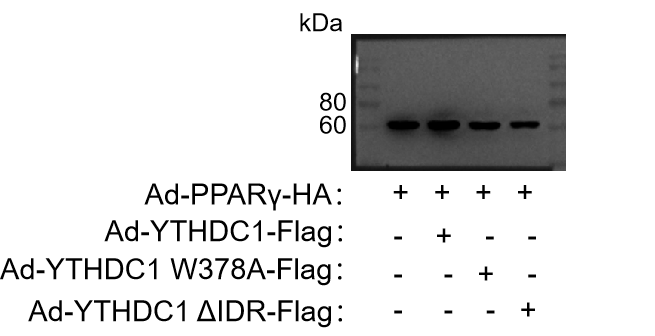

Supplement: Supplementary file 12 — Appendix Figure Source Data [file 44318_2025_460_MOESM12_ESM.zip › Figure S6/S6B/western Input HA.tif]

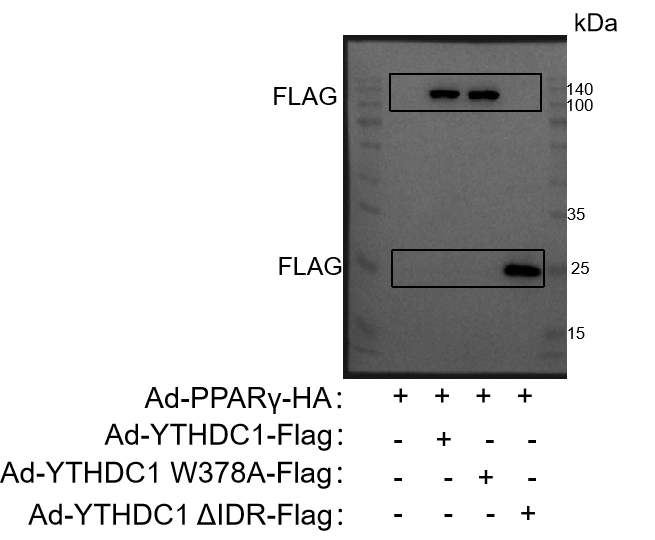

Supplement: Supplementary file 12 — Appendix Figure Source Data [file 44318_2025_460_MOESM12_ESM.zip › Figure S6/S6B/western Input FLAG.tif]

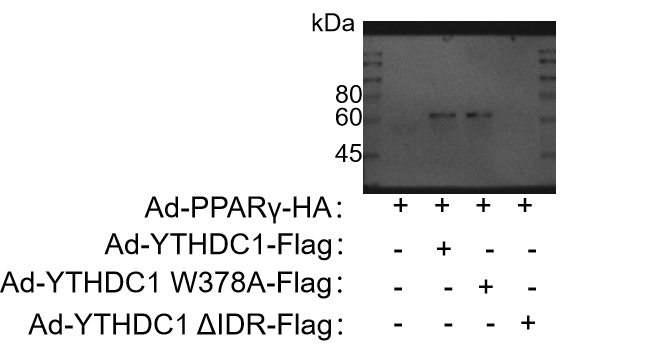

Supplement: Supplementary file 12 — Appendix Figure Source Data [file 44318_2025_460_MOESM12_ESM.zip › Figure S6/S6B/western IP HA.tif]

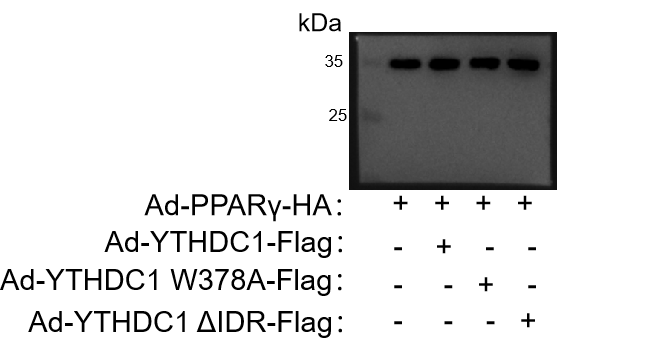

Supplement: Supplementary file 12 — Appendix Figure Source Data [file 44318_2025_460_MOESM12_ESM.zip › Figure S6/S6B/western GAPDH.tif]

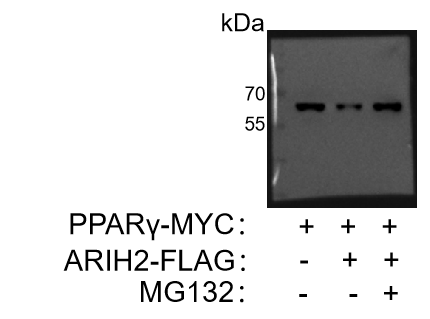

Supplement: Supplementary file 12 — Appendix Figure Source Data [file 44318_2025_460_MOESM12_ESM.zip › Figure S7/S7C/western Input HA.tif]

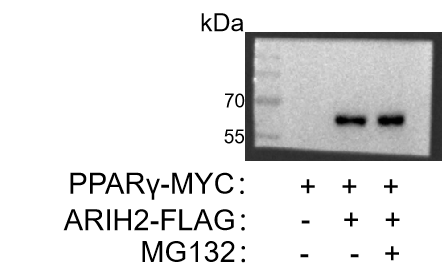

Supplement: Supplementary file 12 — Appendix Figure Source Data [file 44318_2025_460_MOESM12_ESM.zip › Figure S7/S7C/western Input FLAG.tif]

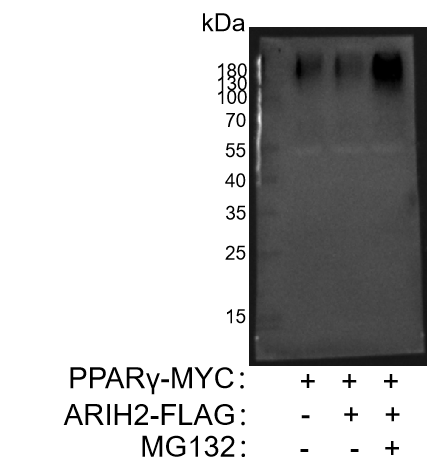

Supplement: Supplementary file 12 — Appendix Figure Source Data [file 44318_2025_460_MOESM12_ESM.zip › Figure S7/S7C/western IP Ub.tif]

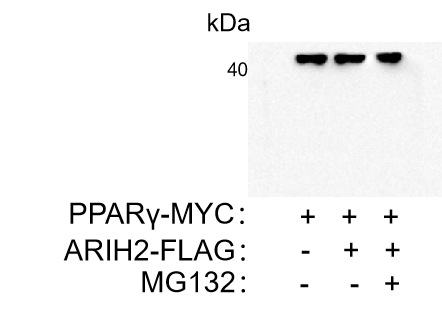

Supplement: Supplementary file 12 — Appendix Figure Source Data [file 44318_2025_460_MOESM12_ESM.zip › Figure S7/S7C/western ╬▓-actin.tif]

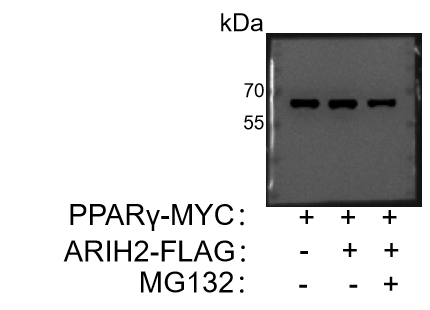

Supplement: Supplementary file 12 — Appendix Figure Source Data [file 44318_2025_460_MOESM12_ESM.zip › Figure S7/S7C/western IP HA.tif]

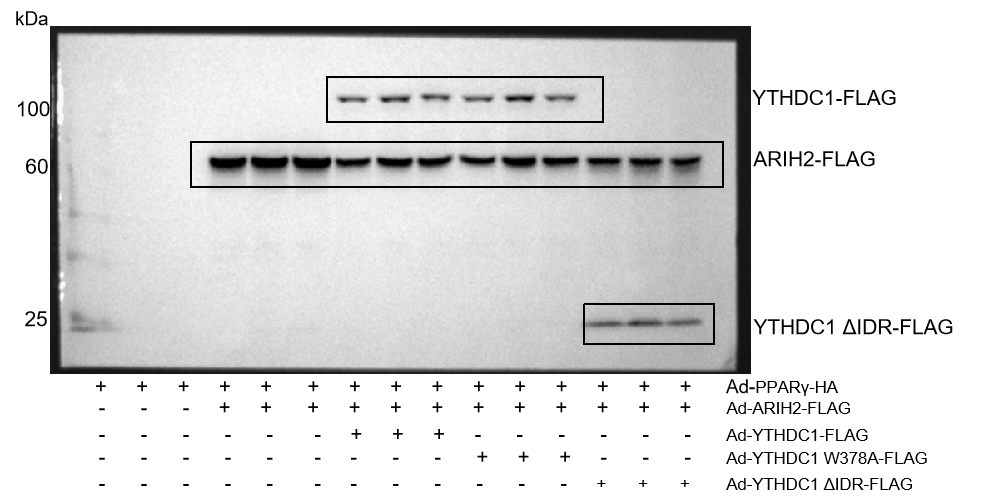

Supplement: Supplementary file 12 — Appendix Figure Source Data [file 44318_2025_460_MOESM12_ESM.zip › Figure S8/S8A/western FLAG.tif]

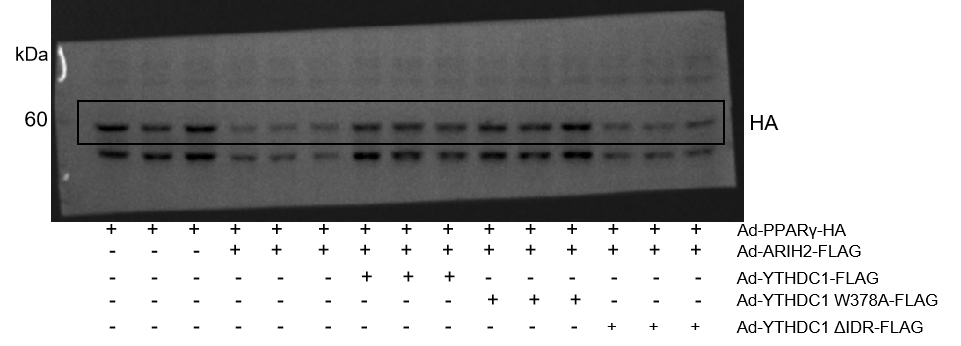

Supplement: Supplementary file 12 — Appendix Figure Source Data [file 44318_2025_460_MOESM12_ESM.zip › Figure S8/S8A/western HA.tif]

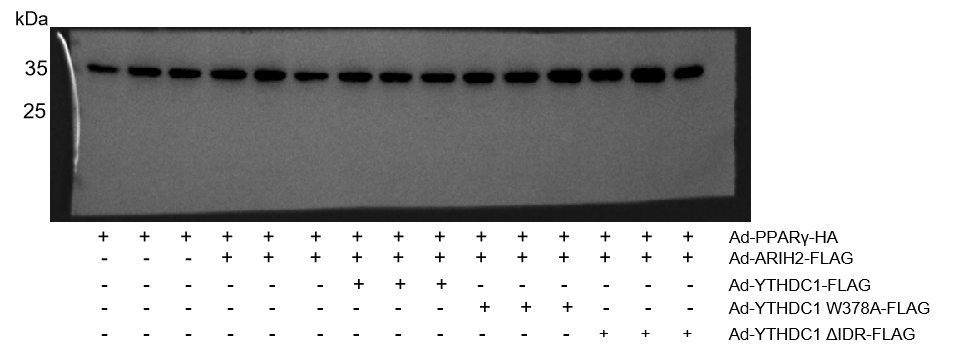

Supplement: Supplementary file 12 — Appendix Figure Source Data [file 44318_2025_460_MOESM12_ESM.zip › Figure S8/S8A/western GAPDH.tif]

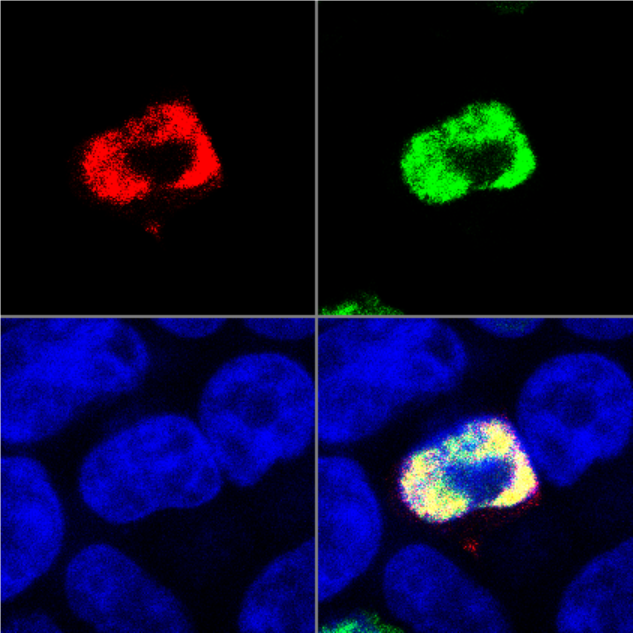

Supplement: Supplementary file 12 — Appendix Figure Source Data [file 44318_2025_460_MOESM12_ESM.zip › Figure S8/S8B/confocal PPAR╬│-HA+YTHDC1 W378A-FLAG.tif]

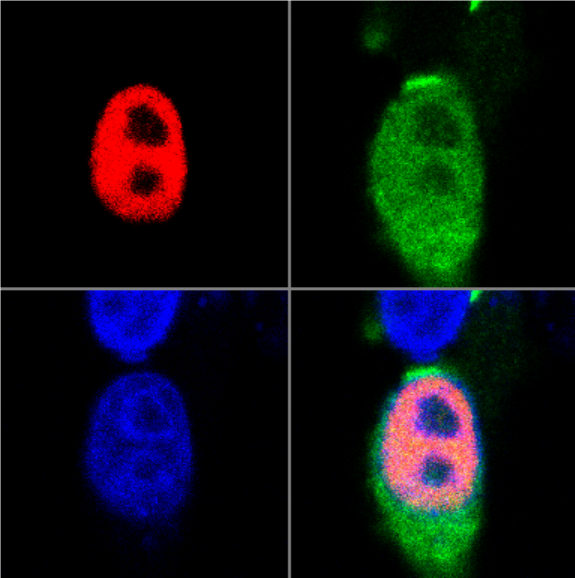

Supplement: Supplementary file 12 — Appendix Figure Source Data [file 44318_2025_460_MOESM12_ESM.zip › Figure S8/S8B/confocal PPAR╬│-HA+YTHDC1 ╬öIDR-FLAG.tif]

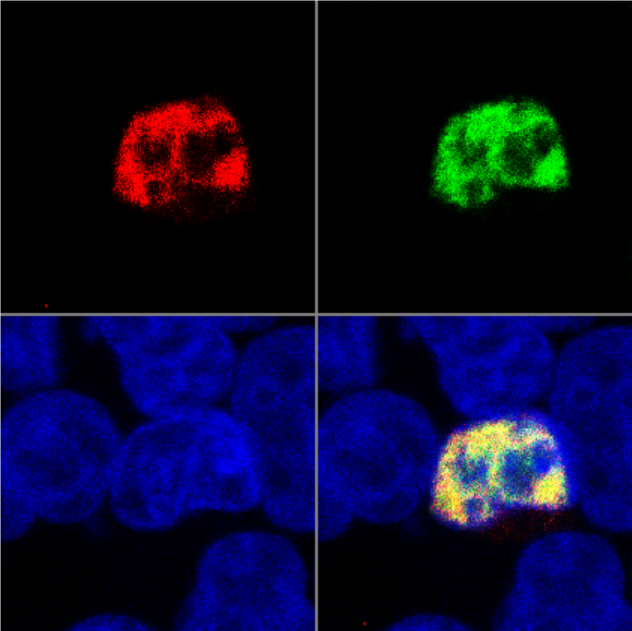

Supplement: Supplementary file 12 — Appendix Figure Source Data [file 44318_2025_460_MOESM12_ESM.zip › Figure S8/S8B/confocal PPAR╬│-HA+YTHDC1-FLAG.tif]

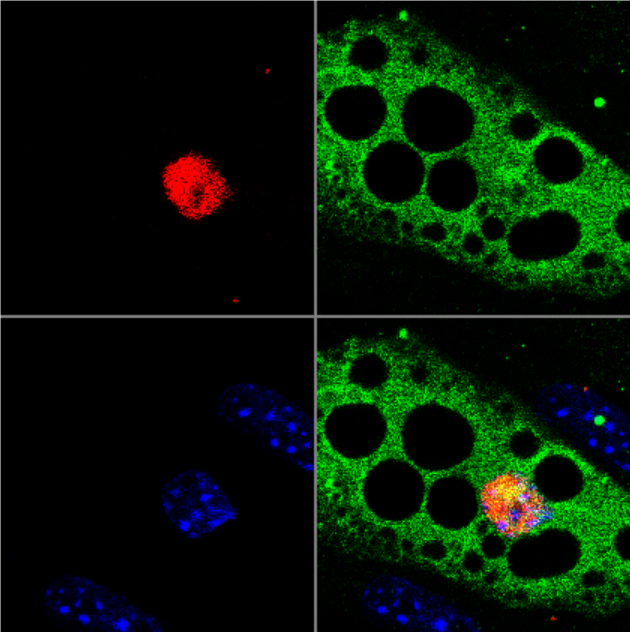

Supplement: Supplementary file 12 — Appendix Figure Source Data [file 44318_2025_460_MOESM12_ESM.zip › Figure S8/S8E/confocal Ad-PPAR╬│-HA+Ad-YTHDC1 ╬öIDR-GFP.tif]

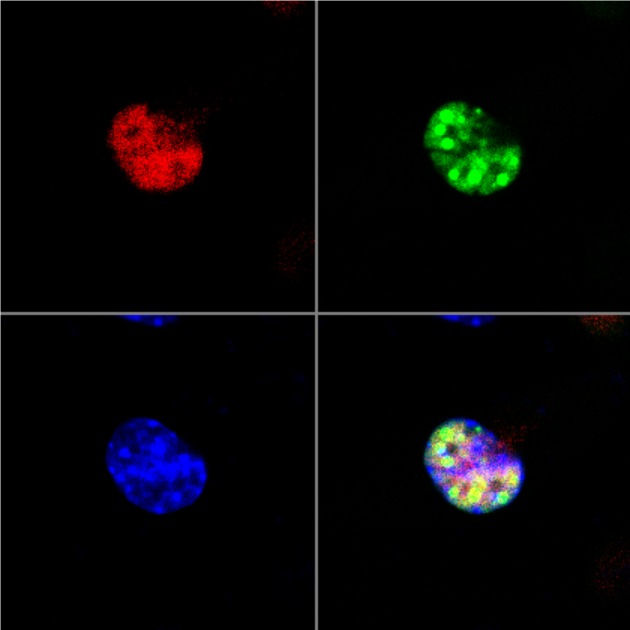

Supplement: Supplementary file 12 — Appendix Figure Source Data [file 44318_2025_460_MOESM12_ESM.zip › Figure S8/S8E/confocal Ad-PPAR╬│-HA+Ad-YTHDC1-GFP.tif]

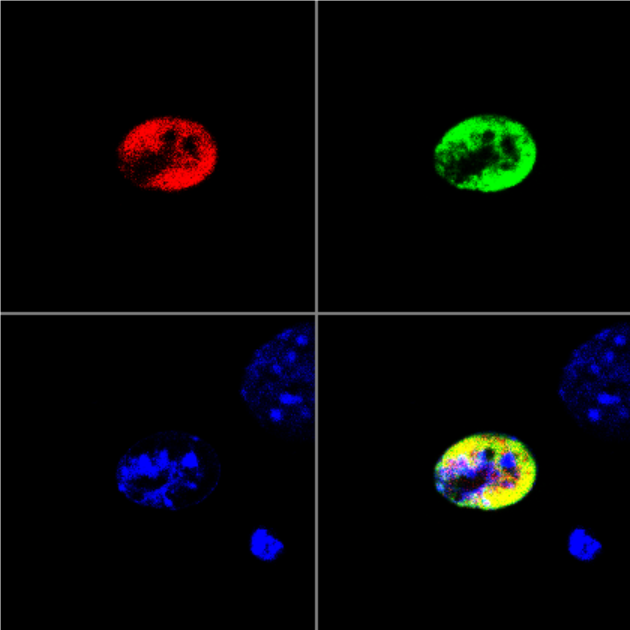

Supplement: Supplementary file 12 — Appendix Figure Source Data [file 44318_2025_460_MOESM12_ESM.zip › Figure S8/S8E/confocal Ad-PPAR╬│-HA+Ad-YTHDC1 W378A-GFP.tif]
