# Supplementary figures and images for: YTHDC1 promotes postnatal brown adipose tissue development and thermogenesis by stabilizing PPARγ (part 3 of 3)
Source: EMBO J. 2025 May 12;44(12):3360–80. doi: 10.1038/s44318-025-00460-x (PMC12170836; doi:10.1038/s44318-025-00460-x)

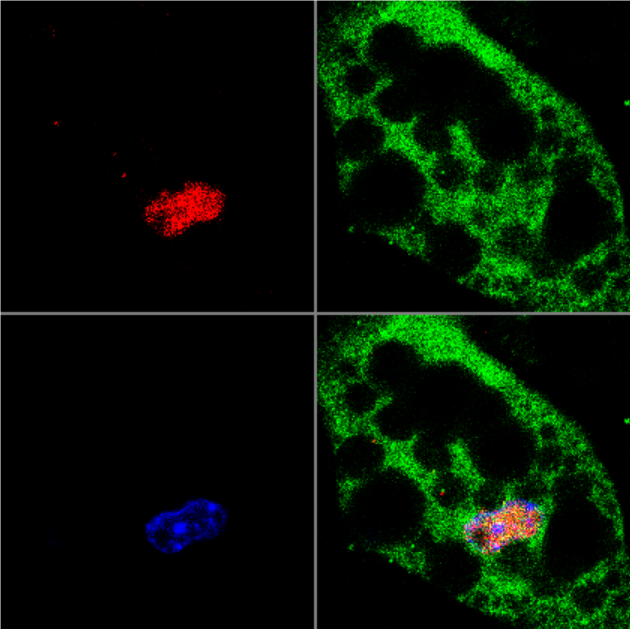

Supplement: Supplementary file 12 — Appendix Figure Source Data [file 44318_2025_460_MOESM12_ESM.zip › Figure S8/S8D/confocal Ad-PPAR╬│-HA+Ad-YTHDC1 ╬öIDR-FLAG.tif]

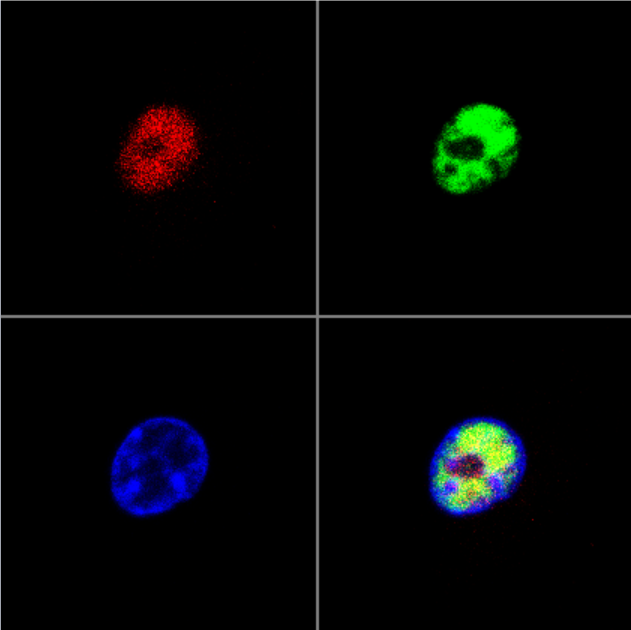

Supplement: Supplementary file 12 — Appendix Figure Source Data [file 44318_2025_460_MOESM12_ESM.zip › Figure S8/S8D/confocal Ad-PPAR╬│-HA+Ad-YTHDC1 W378A-FLAG.tif]

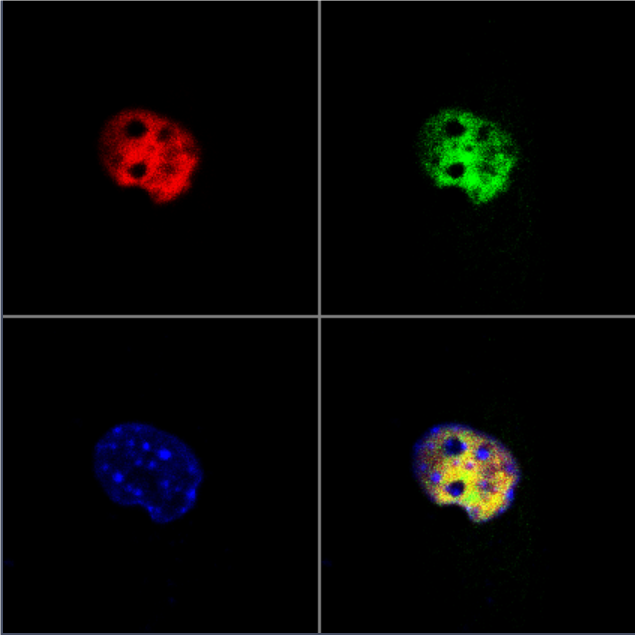

Supplement: Supplementary file 12 — Appendix Figure Source Data [file 44318_2025_460_MOESM12_ESM.zip › Figure S8/S8D/confocal Ad-PPAR╬│-HA+Ad-YTHDC1-FLAG.tif]

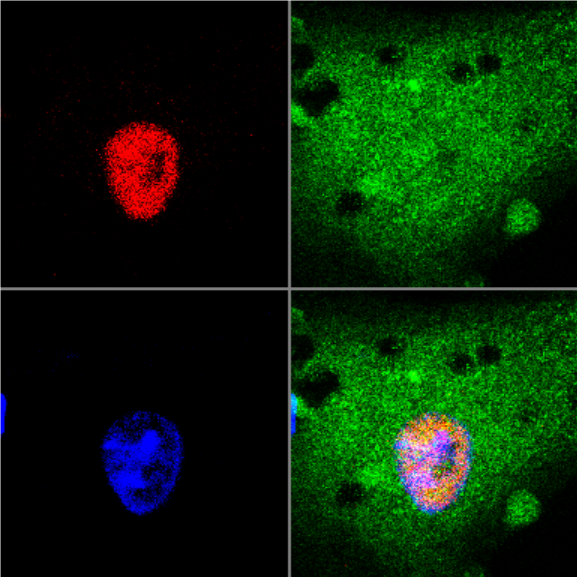

Supplement: Supplementary file 12 — Appendix Figure Source Data [file 44318_2025_460_MOESM12_ESM.zip › Figure S8/S8C/confocal Ad-PPAR╬│-HA+Ad-YTHDC1 ╬öIDR-FLAG.tif]

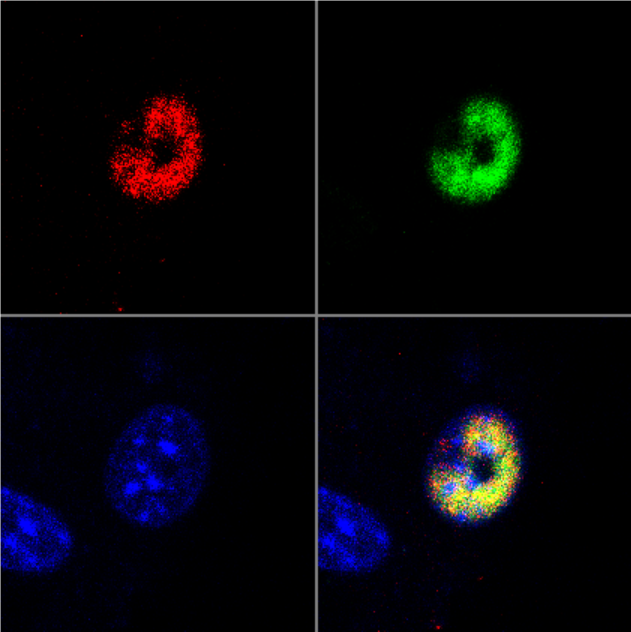

Supplement: Supplementary file 12 — Appendix Figure Source Data [file 44318_2025_460_MOESM12_ESM.zip › Figure S8/S8C/confocal Ad-PPAR╬│-HA+Ad-YTHDC1 W378A-FLAG.tif]

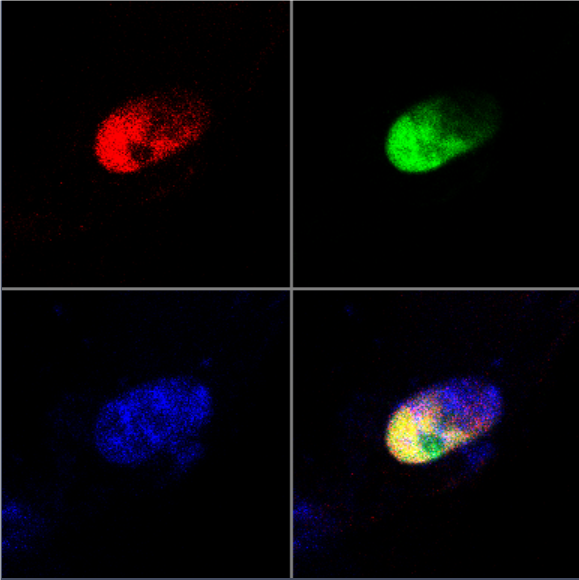

Supplement: Supplementary file 12 — Appendix Figure Source Data [file 44318_2025_460_MOESM12_ESM.zip › Figure S8/S8C/confocal Ad-PPAR╬│-HA+Ad-YTHDC1-FLAG.tif]
